# New records of a lost species and a geographic range expansion for sengis in the Horn of Africa

**DOI:** 10.7717/peerj.9652

**Published:** 2020-08-18

**Authors:** Steven Heritage, Houssein Rayaleh, Djama G. Awaleh, Galen B. Rathbun

**Affiliations:** 1 Division of Fossil Primates, Duke Lemur Center, Duke University, Durham, NC, USA; 2 Interdepartmental Doctoral Program in Anthropological Sciences, Stony Brook University, Stony Brook, NY, USA; 3 Association Djibouti Nature, Djibouti City, Republic of Djibouti; 4 Department of Environment and Sustainable Development, Ministry of Urban Affairs, Environment and Tourism, Djibouti City, Republic of Djibouti; 5 Institute of Biodiversity Science and Sustainability, California Academy of Sciences, San Francisco, CA, USA

**Keywords:** Somali Sengi, Sengis, Elephant-shrews, Macroscelidea, Djibouti, Somalia, Conservation, Phylogenetic Systematics, Taxonomy, Biogeography

## Abstract

The Somali Sengi or Somali Elephant-shrew (*‘Elephantulus’ revoilii*, Macroscelidea, Mammalia) has been considered a “lost species” and is primarily known from about 39 museum specimens, with no new vouchered occurrence records since the early 1970s. The scientific literature contains no data concerning living Somali Sengi individuals and the species’ current Data Deficient conservation status is attributable to an absence of modern information. Almost everything that has been published about the species is derived from anatomical examinations of historic specimens, gleaned from museum collection notes, or inferred from the known habits and ecology of other sengi taxa. Here we report new evidence that the Somali Sengi *is currently extant*. These data include voucher specimens, georeferenced occurrence localities, body measurements, habitat parameters, and DNA sequences. While the species is historically documented as endemic to Somalia, these new records are from the neighboring Republic of Djibouti and thus expand the Somali Sengi’s known range in the Horn of Africa. Furthermore, Djiboutian locality data near international borders suggests that the Somali Sengi is also a current inhabitant of both Somalia and Ethiopia. Criteria that inform conservation status assessments (e.g., suitable habitat contiguity and occurrence in wildlife protected areas) can be positively characterized in Djibouti and therefore bode well for the survival of the Somali Sengi species. New data also inform previously undocumented substrate and sheltering affiliations. DNA analyses indicate that the Somali Sengi is a descendant of the Macroscelidini lineage and therefore reveal that the species’ referral to the genus *Elephantulus* is incompatible with sengi phylogeny. This taxonomic issue is resolved by recognizing a new genus replacement and recombinant binomial that redesignates the Somali Sengi as *Galegeeska revoilii* (gen. nov., nov. comb). An analysis of ancestral biogeography suggests that the Somali Sengi has inhabited the Horn of Africa for more than 5.4 million years—and the recognition of the species’ phylogenetic ancestry appends the already remarkable biogeographic story of the Macroscelidini tribe.

## Introduction

The twenty extant species of elephant-shrews, more properly called sengis and more formally called macroscelideans, are African mammals that comprise the taxonomic order Macroscelidea. All sengi taxa are predominantly insectivorous feeders and all are small bodied with various species ranging from mouse-to-squirrel sizes (Heritage, 2018). Sengis are remarkably adapted runners, with some species able to bound at nearly 30 km/h and with hindlimb proportions closer to gazelles than other cursorial small mammals (Lovegrove & Mowoe, 2014). All sengis studied so far are socially monogamous with pairs that mate for life and that share relatively well-defined home ranges (Rathbun, 2009). Most of these taxa give birth to highly precocial neonates some of which are able to sprint within one hour of parturition (Rathbun, 1979; Rathbun & Rathbun, 2006). The sengi lineage is most closely related to tenrecs, golden-moles, and aardvarks (Meredith et al., 2011; Springer et al., 2003) and the fossil record of Macroscelidea documents a history of endemism on the African continent that extends back at least 45.6 million years (Hartenberger, 1986; Holroyd, 2010; Seiffert, 2010a).

Several sengi species, particularly in eastern and southern Africa, have been relatively well-studied with reported data concerning distribution, habitat, life history, and other attributes sufficient to inform reliable conservation status assessments. However, a few species have been nearly absent from the scientific literature with their geography known only from collection locality records associated with a limited number of historical museum specimens (Perrin & Rathbun, 2013). With little or no *modern* information regarding their distribution or basic biology, the IUCN Red List of Threatened Species has assigned Data Deficient statuses for these sengi taxa (Rathbun, 2015a, 2015b, 2015c). Among these least-known macroscelideans is the Somali Sengi (‘*Elephantulus*’ *revoilii*) which Global Wildlife Conservation has identified as a Top 25 Most Wanted taxon in their Search for Lost Species initiative (Global Wildlife Conservation, 2017; National Geographic, 2017). To our knowledge, it has been nearly 50 years since any institution has vouchered new Somali Sengi specimens (Rathbun, Agnelli & Innocenti, 2014) and there have never been scientific reports that document living individuals.

The Somali Sengi has been considered endemic to Somalia and is represented by about 39 accessioned specimens held in world museum collections. All but one of these individuals inhabited the northern branch or horn of Somalia (roughly historic British Somaliland and northern Italian Somaliland) and most occurrence localities are less than 150 km from the Gulf of Aden shoreline (Rathbun, Agnelli & Innocenti, 2014). The original and holotype specimen is in Paris at the National Museum of Natural History and was collected by French naturalist Georges Révoil in the northern Majeerteen territory of Somalia during his 1878–1881 expedition (Révoil et al., 1882). In 1881, Hüet published the initial description of this new sengi species and named it *Macroscelides revoilii* in honor of its discoverer. From 1891 to 1973, a few expeditions by British, American, and Italian scientists collected the remainder of the known hypodigm.

Only a single research study, conducted by Corbet and Hanks and published in 1968, has included multiple Somali Sengi specimens (*n* = 15) to assess the species’ morphological characters and distribution. The year of this manuscript is often cited as the latest possible date to document the Somali Sengi *as extant—*though this is inaccurate given the few specimens collected up to five years later which are now held at the University of Florence’s Natural History Museum (Rathbun, Agnelli & Innocenti, 2014). Corbet and Hanks’ valuable contribution to the sengi literature makes explicit most of the anatomical traits that are still used to identify the majority of macroscelidean taxa. For the Somali Sengi, this suite of characters includes: (1) a conspicuous pale ring that surrounds the eye which is accompanied by a dark brown post-ocular mark that interrupts the ring; (2) the presence of hair growing on the lower rhinarium; (3) second upper incisors that are about equal in size to both the first and third upper incisors; (4) absence of lower third molars; (5) lower first premolars with only one root; (6) a pectoral gland fringed with short white hairs; (7) a relatively hairy tail with a tuft formed by longer brown-tipped hairs growing near the tail’s distal end; (8) a relatively long tail that is, on average, about 19% longer than head-body length; and (9) head-body length within the range of 122–148 mm (*n* = 6). Several of these characters require close examination to properly assess. However, in practice, a non-specialist in a collections or field setting can identify an individual as a Somali Sengi with reasonable accuracy using only a few traits if they are clearly visible. If a sengi has the distinctive “spectacled” facial pattern (i.e., a pale eye-ring that is broken by a dark post-ocular spot), it can only be one of three species—the Somali Sengi, the Rufous Sengi (*Elephantulus rufescens*), or the Four-Toed Sengi (*Petrodromus tetradactylus*). Within this group, only the Somali Sengi has relatively long tail hair that forms a tuft at the tail’s tip. The other two taxa have very short tail hairs with exposed skin that can give the tail a naked and somewhat scaly appearance.

Important to any discussion of Somali Sengis is their phylogenetic and biogeographic stations within the subfamily Macroscelidinae, commonly called the Soft-Furred Sengi group (Rathbun, 2009). While the three sengi species (Somali, Rufous, and Four-Toed) share a spectacled facial pattern, it is evident by an assemblage of discrete traits, skeletal proportions, and body size, that the Four-Toed Sengi (which is placed in its own genus) is substantially different from the other two taxa (Corbet & Hanks, 1968; Evans, 1942). In addition to a spectacled eye, the Somali Sengi and Rufous Sengi uniquely share hair growth on the lower rhinarium. These two traits are the basis for a systematic hypothesis which arranges the Somali and Rufous Sengis as sister-species (Corbet, 1995; Corbet & Hanks, 1968; Koontz & Roeper, 1983). Compared to the Somali Sengi, about which there is very little information and geographic distribution is rather confined, there is a relatively large body of literature about the Rufous Sengi which has six subspecies and a broad range that includes parts of Tanzania, Kenya, Uganda, South Sudan, and Ethiopia (Koontz & Roeper, 1983; Perrin & Rathbun, 2013). A single subspecies of Rufous Sengi (the Somalian Rufous Sengi, *Elephantulus rufescens somalicus*) occurs in northern Somalia, but like the Somali Sengi, this is only documented in a limited hypodigm of historic museum specimens. Taken together, these data suggest that the Rufous Sengi’s range in eastern Africa extends to the north where it abuts or overlaps with the range of its supposed closest relative, the Somali Sengi (Corbet & Hanks, 1968).

In the central portion of northern Somalia, there are two localities where the Somali Sengi and the Somalian Rufous Sengi have both been documented. It appears that, at least in the early 1900s, these taxa were locally sympatric. However, field notes indicate that the two differ in their habitats—the Somali Sengi was found in areas with rocky terrain and the Somalian Rufous Sengi among bushes on sandy soils (Corbet & Hanks, 1968). Elsewhere in Africa, the habitats of Rufous Sengis are well-understood; they occur in arid woodlands and bushlands with compact sandy soils, open canopies, scrub vegetation, and very little grass (Koontz & Roeper, 1983; Perrin & Rathbun, 2013; Rathbun, 1979). It seems that the Somalian Rufous Sengi and other Rufous Sengi subspecies are consistent in their habitats and substrate use. Whether or not the Somali Sengi is an obligate rock-dweller has not been documented. However, in southern Africa, where several sengi species are well-studied, there are noteworthy comparisons. Here the Western Rock Sengi (*Elephantulus rupestris*) and the Bushveld Sengi (*Elephantulus intufi*) are phylogenetic sister-species with overlapping geographic ranges. The former uses stony substrates (rupicolous) and shelters among boulders and the later uses compact sandy loams (arenicolous) and shelters at the bases of bushes. These two species can occur adjacently where scrublands meet rocky outcrops but are, in essence, divided by their use of habitats (Heritage, 2018). It could be the case that a similar scenario is at-play in the Horn of Africa.

Here we report that the Somali Sengi is currently extant and convey data from the first newly vouchered individuals since 1973. These new records are from the Republic of Djibouti—to the northwest and adjacent to northern Somalia—and considerably extend the species’ documented distribution. Data are from multiple animals and multiple sites, and when combined with interview information from local people, suggest that comparative species abundance is not rare. Within Djibouti, the Somali Sengi is widespread and certainly rupicolous, using the rugged rocky habitats present throughout the country. The types of conservation threats that impact other sengi species are not intense in Djibouti. Also reported are new Somali Sengi DNA sequences and accompanying phylogenetic analyses which yield a necessary framework for taxonomic reassessment, the estimation of diversification ages, and modeling ancestral biogeography.

## Methods

### Fieldwork

Fieldwork in Djibouti was conducted for 15 consecutive dates beginning on February 1, 2019. At scouted localities, H.B. Sherman brand folding aluminum live-traps (3 × 3.5 × 9 in.) were set each afternoon beginning at roughly 17:00 and collected the following morning before 07:00. Each setting included placing 80–90 traps in multiple transects and spaced 10 paces apart. Traps were baited with a dry mixture of whole rolled oats, unsweetened peanut butter, and yeast spread. A pinch of this mixture was added inside each trap and at its entrance. In total, 1,259 traps were set at 12 localities. A Garmin model GPS 60 handheld global positioning unit was used to document GPS coordinates for each locality and the georeferenced dataset was collected and proofed using Google Earth Pro v7.3.2. Four fieldwork areas were chosen based on previous sengi sightings (or potential sengi sightings) with observations being either first-hand (coauthor H.R.) or communicated to our team by François Turpin and Roman Bourgeais. Specific trapping localities were determined daily by assessing terrain, sheltering potential, and dung piles at candidate sites—and by considering information from local interviewees about small mammals in the area. Authorization to work, collect, and export was granted by Djibouti’s Department of Environment and Sustainable Development, Ministry of Urban Affairs, Environment and Tourism (Document 619|DEDD|18). All work with live animals followed the standard guidelines of the American Society of Mammalogists and these methods were approved for this research by the Institutional Animal Care and Use Committee at the California Academy of Sciences (CAS IACUC approval number 2019-1).

We collected standard specimen measurements using a hanging analog scale with 0.1 g precision and a ruler with 1 mm precision precision. (1) Body mass was measured by full suspension. (2) Total length was measured by placing the specimen’s ventrum on a ruler and lightly depressing its dorsum. This metric is defined as the distance along the ventral body from the tip of the nose to the fleshy tip of the tail (i.e., excludes tail tuft hairs). (3) Tail length was measured along the tail’s ventral side from the proximal base near the anus to the fleshy distal tip. We note that standard head-body length can be derived by subtracting tail length from total length and that relative tail length is tail length divided by head-body length. (4) Foot length was measured on the right pes from the hind edge of the heel to the distal tip of the longest claw. (5) Ear length was measured on the right pinna from notch to crown.

In the field, fresh tissues (e.g., liver, muscle) were removed using sterile instruments and gloves and then immediately plunged in 95% ethanol. Whole body specimens were submerged in 10% buffered formalin until fully cured and then transferred to ethanol for storage. A few specimens were prepared as classical study skins and skeletons. All new animals and tissues have been deposited in the research collections at the CAS Department of Ornithology and Mammalogy where work, export and import authorization documents are also maintained. See Table 1 for specimen catalog numbers and preparation types.

**Table 1:** Measurements and locality data for the Somali Sengi in Djibouti.

Catalog number (w/ prefix CASMAM) & Field ID	Total length (mm)	Tail length (mm)	Head- body length (mm)	Rel. tail length (%)	Pes length (mm)	Ear length (mm)	Mass (g)	Sex	Col. Date (2019) & Site	Decimal GPS (Lat° N, Lon° E)	Alt. (m)	Specimen Prep & DNA seq
32723 GBR941	280	151	129	117.1	38	19	54+11^x^	F	02 Feb *a* (east)	11.36906, 42.82961	663	*WB,T* yes
32724 GBR945	294	156^#^	138	–	38	24	49	M	03 Feb *a* (west)	11.36464, 42.83104	665	*WB,T* no
32725 GBR953	260	140	120	116.7	35	29	41	M	11 Feb *b*	11.01915, 42.87870	538	*WB,T* yes
32728 GBR955	275	148	127	116.5	38	25	51	M	12 Feb *b*	11.01946, 42.87882	560	*SSS,T* no
32726 GBR957	250	121^#^	129	–	38	24	49	M	15 Feb *c*	11.51955, 42.87431	557	*WB,T* yes
32729 GBR958	286	157	129	121.7	37	26	59	M	15 Feb *c*	11.52188, 42.87577	551	*SSS,T* no
32727 GBR959	270	145	125	116	36	24	46	F	15 Feb *c*	11.52168, 42.87577	551	*WB,T* no
32730 GBR960	285	153	132	115.9	39	27	50	F	16 Feb *c*	11.52234, 42.87638	524	*SSS,T* no
uncollected sighting*	–	–	–	–	–	–	–	–	08 Feb *d*	11.78317, 42.64082	1489	–
uncollected sighting	–	–	–	–	–	–	–	–	11 Feb *b*	11.01817, 42.87772	541	–
uncollected sighting	–	–	–	–	–	–	–	–	12 Feb *b*	11.01848, 42.87986	555	–
uncollected sighting	–	–	–	–	–	–	–	–	14 Feb *c*	11.51936, 42.87501	560	–
**Mean**			**128.6**	**117.3**	**37.4**	**24.8**	**49.9**					

**Notes:**

* Photographed.

# Tail tip missing.

x Mother+fetus.

*a* Djibouti, Arta Region, Djalelo Protected Area, Hansane Hill.

*b* Djibouti, Ali Sabieh Region, Assamo Decan Camp, South Ridge Base.

*c* Djibouti, Arta Region, Arta Town, Oú Est Mon Camarade Hill.

*d* Djibouti, Tadjoura Region, Goda Mountain, Day Forest Village.

*WB,T* Whole Body (in fluid) & Tissues.

*SSS,T* Skin, Skull, Skeleton & Tissues.

### DNA extraction and purification

Three specimens, one representing each of the new Djiboutian collection sites, were selected for genetic sequencing (GBR941 Djalelo locality, GBR953 Assamo locality, GBR957 Arta Town locality). Lab work closely followed previously published protocols (Heritage et al., 2016). Total DNA was extracted from ethanol preserved liver samples using a Qiagen DNEasy Blood and Tissue Kit. The recommended lysis protocol was modified by increasing the volumes of Proteinase K and buffer ATL to 40 µl and 360 µl, respectively—and by increasing incubation time to 36 h. Total DNA yield was promoted by opting to double-elute the final spin column. We processed a negative control by following identical methods but substituting nuclease-free water for donor tissue.

### DNA amplification and sequencing

PCR amplifications targeted two nuclear (IRBP, VWF) and four mitochondrial (12S, 16S, COX1, CYTB) loci. We used a comparative sample of existing macroscelidean and other afrothere sequences (from GenBank) to design novel amplification primers. Promega GoTaq G2 Hot Start Master Mix and protocols were used to perform 50 µl (nuclear) and 25 µl (mitochondrial) reactions. Each PCR run consisted of 40 cycles—preceded by an initial denaturation (5 min, 95 °C) and followed by a final extension (5 min, 72 °C). Each cycle comprised 1 min of denaturation at 95 °C, 1 min of annealing at variable temperatures, and a 72 °C extension period of variable times. Primer sequences and their associated annealing and extension metrics are reported in Data S1.

Since the availability of Somali Sengi voucher specimens is quite rare, we elected to expand the genetic data available for future comparative studies by amplifying and sequencing the full mitochondrial genomes of the three specimens. Sequence results from the four targeted mitochondrial loci were used to construct unambiguous amplification primers near segment flanks and their accompanying second strand primers were newly designed using a GenBank sample of comparative taxa. With this strategy, and with the four loci used as seeding points, work moved stepwise in both directions to produce overlapping segments that extended mitogenomic sequences until loops were complete. The additional mitochondrial primers are also reported in Data S1.

We screened PCR products for expected lengths and non-specifics using electrophoresis through a 1% agarose gel. All negative control runs resulted in empty lanes. Excised gel slices containing banded DNA products were purified and double-sequenced at a commercial facility (Eurofins Operon, Applied Biosystems 3730xl analyzer). Each sequencing result was BLAST searched against the NCBI database to screen for potential contamination. All queries indicated that resultant DNA sequences are unique and have high percent identity relative to other macroscelidean taxa. No other sengi species have been previously processed in the lab. In silico translations of amino acid coding segments contained no unexpected stop codons. Overlapping mitochondrial segments were mapped to the previously published mitogenomes of the Soft-Furred Sengis *Macroscelides proboscideus* (GenBank accession NC_004026) and *Elephantulus edwardii* (NC_041486). Geneious v7.1.7 (Kearse et al., 2012) was used for mapping, assembly and annotations. The new *revoilii* DNA sequences are deposited at GenBank with accession numbers MT354760–MT354768.

### Comparative DNA dataset

In addition to *revoilii* sequences, our comparative DNA dataset collects six gene segments from 45 other afrothere taxa including all but two macroscelidean species (see “Discussion”). Sequences were retrieved from GenBank and special care was taken to identify source specimens for sengi taxa. Priority of inclusion in the dataset was given to sequences derived from vouchered animals with verifiable taxonomy. GenBank accession numbers and source specimen information are reported in Data S2.

Alignments were performed using the standard Geneious toolkit. For amino acid coding loci, the multiple align tool and default settings were used to produce initial alignments and these were refined manually with respect to translation reading frames. For rRNA coding loci, we used previously published structural models of both ribosomal subunits to identify and annotate stem and non-stem base positions within each included sequence (Burk, Douzery & Springer, 2002; Cannone et al., 2002; Springer & Douzery, 1996). Alignments of rRNA coding segments proceeded by adding sequences one at a time and manually adjusting positions with respect to stem and non-stem annotations. Some non-stem regions are variable in length across the taxon sample, and in these cases, algorithm-driven alignments were applied regionally using the Geneious multiple align tool. These results were then visually assessed and areas containing alignment ambiguity were removed. Within ribosomes, stem and non-stem positions are subject to different evolutionary pressures and isolating the two types allows for phylogenetic analysis where, if justified, they can be treated with independent substitution models (Kjer, Roshan & Gillespie, 2009; Roberts et al., 2011; Springer & Douzery, 1996). Finally, all individual gene segment alignments were concatenated yielding the 7,307 position supermatrix provided in Data S3. Nuclear and mitochondrial portions of this dataset are 2,505 and 4,802 alignment positions, respectively.

### Phylogenetic analysis

PartitionFinder v2.1.1 (Guindon et al., 2010; Lanfear et al., 2016) was used to select a subset scheme and DNA substitution models. Input data blocks were defined by gene, codon position (amino acid coding loci), and structural position (rRNA coding loci). PartitionFinder settings directed exploration of all models available in MrBayes and all possible combinatorial subset schemes given the input data blocks. The Bayesian Information Criterion (BIC) was used to evaluate a best-fit scheme and corresponding models (see Data S3).

We performed standard Bayesian and time-scaled Bayesian phylogenetic analyses using MrBayes v3.2.6 MPI (Altekar et al., 2004; Ronquist et al., 2012). Partitions and models were specified according to PartitionFinder results and set as unlinked. Markov chain Monte Carlo (MCMC) parameters were set for two runs with four chains each (one cold, three hot) and to sample in 1,000 generation increments. Heating temperature was lowered to 0.05 to promote chain swapping and relative burn-in was set to 10%. Tree distributions were summarized using the MrBayes option for majority rule plus compatible groups. Multiple studies of mammalian phylogenetics have resolved Paenungulata as the sister group of all other extant afrotheres (Meredith et al., 2011; Seiffert, 2007; Springer et al., 2005) and our analyses were thus rooted on the stem lineage of the paenungulate clade.

The time-scaled analysis incorporated paleontological data to calibrate five tree nodes. Included fossils were identified from literature sources to be the geologically oldest known taxa within relevant crown clades. These taxa are: *Miorhynchocyon meswae*, crown-Macroscelidea, 21.0–22.0 Ma (Holroyd, 2010); *Dilambdogale gheerbranti*, crown-Afrosoricida, 36.7–37.8 Ma (Cooper et al., 2014; Seiffert, 2006); *Todralestes variabilis*, crown-Afroinsectivora, 56.0–57.1 Ma (Gheerbrant et al., 1998; Seiffert, 2010b); *Daouitherium rebouli*, crown-Tethytheria, 55.9–56.1 Ma (Kocsis et al., 2014; Seiffert, 2007); *Eritherium azzouzorum*, crown-Afrotheria, 59.2–60.5 Ma (Cooper et al., 2014; Kocsis et al., 2014). Node age calibrations applied truncated normal distributions (TND) where minima and means corresponded to the youngest possible ages of these fossil taxa. Deeper node ages were softly restrained but unbounded (TND:SD = 5 Ma). The clock rate prior was estimated using a previously published R script (Gunnell et al., 2018; Sallam & Seiffert, 2019) and generated with an initial root age estimate at the range-maximum of *Eritherium*. The independent gamma rates (IGR) model was selected to estimate relaxed clock rate variation and parameterized with the MrBayes default IGR variance prior. Time-scaled branch lengths were set to sample from a uniform distribution.

Analysis run lengths were assessed and selected using two diagnostics for convergence and sampling. First, we required run lengths sufficient to yield an average standard deviation of split frequencies (ASDSF) less than 0.01 (Ronquist et al., 2020). Individual runs converging on the same topological distributions should produce an ASDSF diagnostic that approaches zero (Ronquist et al., 2012). Second, we required run lengths sufficient to yield analysis metrics where the minimum estimated sample size (minESS) of all parameters were greater than 200 (Rambaut et al., 2018; Ronquist et al., 2020). Accordingly, both the standard (STD) and time-scaled (TS) analyses were continued for 15 M generations (STD ASDSF = 0.000514 smallest minESS = 478.19; TS ASDSF = 0.000955 smallest minESS = 264.72). Diagnostic values at these run lengths are interpreted as evidence of sufficient convergence and sampling of parameter space. Tree results were visualized and rendered as vector graphics using FigTree v1.4.4 (Rambaut, 2018). Data S3 includes settings for both supermatrix analyses, the STD result with a corresponding genetic distance matrix, and the TS result with divergence age estimates and 95% Highest Posterior Dentistry (HPD) ranges. Additionally, we ran separate standard Bayesian analyses for each gene segment to explore phylogenetic signal within individual loci (Data S4).

### Historic distribution data

We compiled museum records of Somali Sengi vouchers using institutional web portals and by correspondence with collections staff. Where possible, GPS coordinates for collection localities were re-estimated. To do so, locality names in museum ledgers and on specimen tags were cross-referenced with historic maps of British Somaliland, collection notes, elevation data, expedition routes, and literature sources (Great Britain General Staff Geographical Section, 1922; Hansen, 1882; Lanza, 1981; Rathbun, Agnelli & Innocenti, 2014). Google Earth Pro was used to return geocoordinates of pinned locality estimates and to approximate a minimum convex polygon to represent a geographic range for the species (IUCN, 2018). Of the 39 previously known specimens held in 8 institutions, 4 were not included in this GPS dataset. Locality names for 2 of these were ambiguously recorded as “British Somaliland” and thus cannot be localized. A single specimen from Bud Bud (in the eastern branch of Somalia) was excluded because the locality has been considered anomalous pending additional records (Rathbun, Agnelli & Innocenti, 2014). However, if future research further documents the species as far south as Bud Bud, it would considerably expand the known geographic range. The last excluded voucher was the 1881 holotype specimen where the collection notes refer to the “Medjourtine” region of Somalia. While we cannot be certain about a specific site, Révoil’s known expedition route in the northern Majeerteen territory narrows the possibilities (Hansen, 1882). This specimen must be from a site to the east of all other included localities. In total, we identified 15 referenceable localities in northern Somalia from which 35 Somali Sengi specimens were collected. Historical distribution data, along with the new data from Djibouti, are provided in Data S5. We attempted to assemble records from all historically collected specimens but note that an institution unknown to us may hold additional Somali Sengi vouchers.

Within this manuscript and accompanying data supplements, specimen catalog numbers denote the holding institutions with the following codes: AMNH, American Museum of Natural History, New York, NY, USA; CAS, California Academy of Sciences, San Francisco, CA, USA; FMNH, Field Museum of Natural History, Chicago, IL, USA; LACM, Los Angeles County Museum, Los Angeles, CA, USA; MNHN, National Museum of Natural History, Paris, France; MZUF, Museum of Natural History (Zoology, La Specola) at the University of Florence, Florence, Italy; NHMUK, Natural History Museum of the United Kingdom, London, England; NHMO, Natural History Museum at the University of Oslo, Oslo, Norway; SAMA, South Australian Museum, Adelaide, Australia; USNM, Smithsonian National Museum of Natural History, Washington, DC, USA.

### Ecological niche modeling

To explore a potential distribution of the Somali Sengi—in terms of environmental variables that support the species—we used Maxent v3.4.1 GUI (Phillips, Anderson & Schapire, 2006; Phillips & Dudík, 2008) for maximum entropy ecological niche modeling (ENM). Input occurrence records included the 35 specimens from northern Somalia that are associated with referenceable localities. We appended this data with the geocoordinates of all new Djiboutian records of Somali Sengis—including collected specimens and uncollected sightings (*n* = 12, see Table 1). For all Maxent runs described below, we used the default option to remove duplicate presence records (within the same grid-cells) to control for correlation of occurrence localities.

Environmental data included the standard WorldClim v2 Bioclimatic variables + elevation set (*n* = 20) and these data were downloaded at 2.5 arc-min resolutions (Fick & Hijmans, 2017). Data grids of all environmental variables were converted from GeoTiff to ESRI Ascii format using the ‘raster’ package (Hijmans et al., 2020) in R v3.6.1 (R Core Team, 2019). The Horn of Africa was broadly defined as our region of interest (ROI) using the geocoordinate ranges 32–56° longitude and −3° to 22° latitude—and all environmental data was cropped to this region using DIVA-GIS v7.5.0 (Hijmans et al., 2004). Three additional preparation steps were taken prior to the final ENM analysis: (1) identifying and removing highly correlated (collinear) environmental variables; (2) identifying and removing poorly-contributing environmental variables; and (3) testing for and selecting an optimal regularization multiplier (RM) value.

Pairwise correlation tests of all ROI-cropped environmental variables were run using the ‘raster’ package in R where ten pairs were identified with Pearson correlation coefficients >0.9 or <−0.9 (Fourcade et al., 2014; Jueterbock et al., 2016). For each of these pairs, we ran five Maxent test analyses (with logistic output) using a range of RMs—from 1 to 5, in increments of 1—and including only the two variables of the collinear pair. The result of each analysis includes percent contribution of individual variables to the produced model. Within each pair, the variable with the lowest percent contribution was selected for exclusion from the final ENM analysis (*n* = 8 unique collinear variables to exclude). We note that some authors have reported that Maxent distribution models can be robust despite the inclusion of highly correlated variables (Feng et al., 2019), but these models can be overly complex (Jueterbock, 2016) and the resultant response curves and percent contributions can be misleading when doing so (Phillips, 2017).

To address the potential for model over-parameterization (Jueterbock, 2016), we ran five additional Maxent test analyses (with logistic output) that included the full set (*n* = 12) of non-collinear environmental variables and at the same range of RM values previously described. From these results, variables with very poor percent contributions were identified as those yielding 0% at one or more RM treatments (*n* = 7 poorly contributing variables to exclude). Averaged across all five tested RMs, each of these variables contributed <1% to the produced models. After the exclusion of collinear and poorly-contributing environmental variables, the ROI-cropped dataset marked for inclusion in the final ENM analysis was: (A) Bioclim-4 Temperature Seasonality; (B) Bioclim-14 Precipitation of Driest Month; (C) Bioclim-5 Max Temperature of Warmest Month; (D) Bioclim-13 Precipitation of Wettest Month; and (E) Bioclim-19 Precipitation of Coldest Quarter.

To identify an optimal RM value, we ran 75 Maxent test analyses (with raw output), each including the final set of environmental variables and using a range of RMs—from 0.1 to 7.5, in increments of 0.1. ENMTools v.1.4.4 (Warren, Glor & Turelli, 2010) was then used to calculate BIC scores (Warren & Seifert, 2011) for all test runs and the RM value resulting in the lowest BIC was selected for use in the final ENM analysis (RM = 0.7).

Using the 47 Somali Sengi occurrence records, the 5 non-collinear and positively contributing environmental variables, and a statistically justified regularization multiplier (0.7), our final Maxent run specified for logistic output, 10,000 background points, and 20 cross-validated replicates. All other analysis settings used Maxent defaults. Mean results were visualized using DIVA-GIS. The logistic output format assigns a value, ranging from 0 to 1, to all grid-cells in the mapped result. These values can be interpreted as a measure of habitat suitability for the species (Fourcade et al., 2014), given the data used to construct the model. Data S6 includes final analysis output, but excludes WorldClim data which are not redistributable.

### Ancestral biogeography analysis

To assess the ancestral biogeography of sengis in a phylogenetic context, we used BayesTraits v3.0.1 (Pagel & Meade, 2017) for continuous trait modeling of geocoordinates at ancestral lineage splits. In brief, Bayesian ancestral state reconstruction incorporates tree topology, patristic distances (e.g., scaled to time), and trait data for taxa at tree-tips to estimate trait data at internal tree-nodes. The method has been widely applied to both categorical and continuous data. A best practice used when modeling quantitative traits against species-level phylogenies is to code tree-tips with mean values derived from multiple intraspecific measurements (Boyer et al., 2013; Yapuncich et al., 2019). A dedicated geographical module (which accommodates geodesic distances and associations around any directional horizon) uses a Brownian motion model to estimate ancestral positions in a three-dimensional Cartesian coordinates system.

The analysis input phylogeny was adapted from the time-scaled Bayesian tree by pruning all non-macroscelidean taxa and by collapsing subspecies within genus *Rhynchocyon* to single tree-tips at their respective species ranks. Additionally, the *Petrodromus* tree-tip was split to create paired lineages with null branch lengths so that this taxon’s two disjoined geographic ranges could be represented.

Per species distributions were compiled from literature sources (Heritage, 2018; IUCN, 2020; Rathbun, 2017) and these data estimate the current ranges of living sengis. We note that the few fossils that have been referred to extant Macroscelidinae species are relatively young (Plio-Pleistocene) and all are from localities that are within or near the modern ranges of corresponding species—at least within the same countries of occurrence (Holroyd, 2009). A possible exception is *Elephantulus fuscus*, where fossil specimens that have been tentatively referred to the species (Butler, 1995) may indicate a broader than the modern range. Regardless, *E. fuscus* was not included in any of our analyses (see “Discussion”). For tree-tip data, geographic centroids (=spatial means) were derived from perimeter coordinates of species range polygons and calculated with the ‘geosphere’ package (Hijmans, 2019) in R.

Ancestral state reconstruction was performed using the BayesTraits Geo model and the MCMC run was called for 1.5 M generations, with burn-in set at 0.5 M and sampling at 1,000 generation increments. Results were inspected and summarized using Tracer v1.7.1 (Rambaut et al., 2018). The estimated sample size (ESS) of all reconstructed characters (i.e., longitude and latitude at each tree node) were between 593 and 850. ESS values above 200 are interpreted as an indication of sufficient sampling and run length. Input data, settings and full results (including 95% HPD ranges) are provided in Data S7.

### ZooBank registration

The electronic version of this article in Portable Document Format (PDF) will represent a published work according to the International Commission on Zoological Nomenclature (ICZN), and hence the new names contained in the electronic version are effectively published under that Code from the electronic edition alone. This published work and the nomenclatural acts it contains have been registered in ZooBank, the online registration system for the ICZN. The ZooBank Life Science Identifiers (LSIDs) can be resolved and the associated information viewed through any standard web browser by appending the LSID to the prefix http://zoobank.org/. The LSID for this publication is: urn:lsid:zoobank.org:pub:7D882E47-0982-46E7-AA67-20BB71FDD31D. The online version of this work is archived and available from the following digital repositories: PeerJ, PubMed Central and CLOCKSS.

## Results

**Figure 1:**
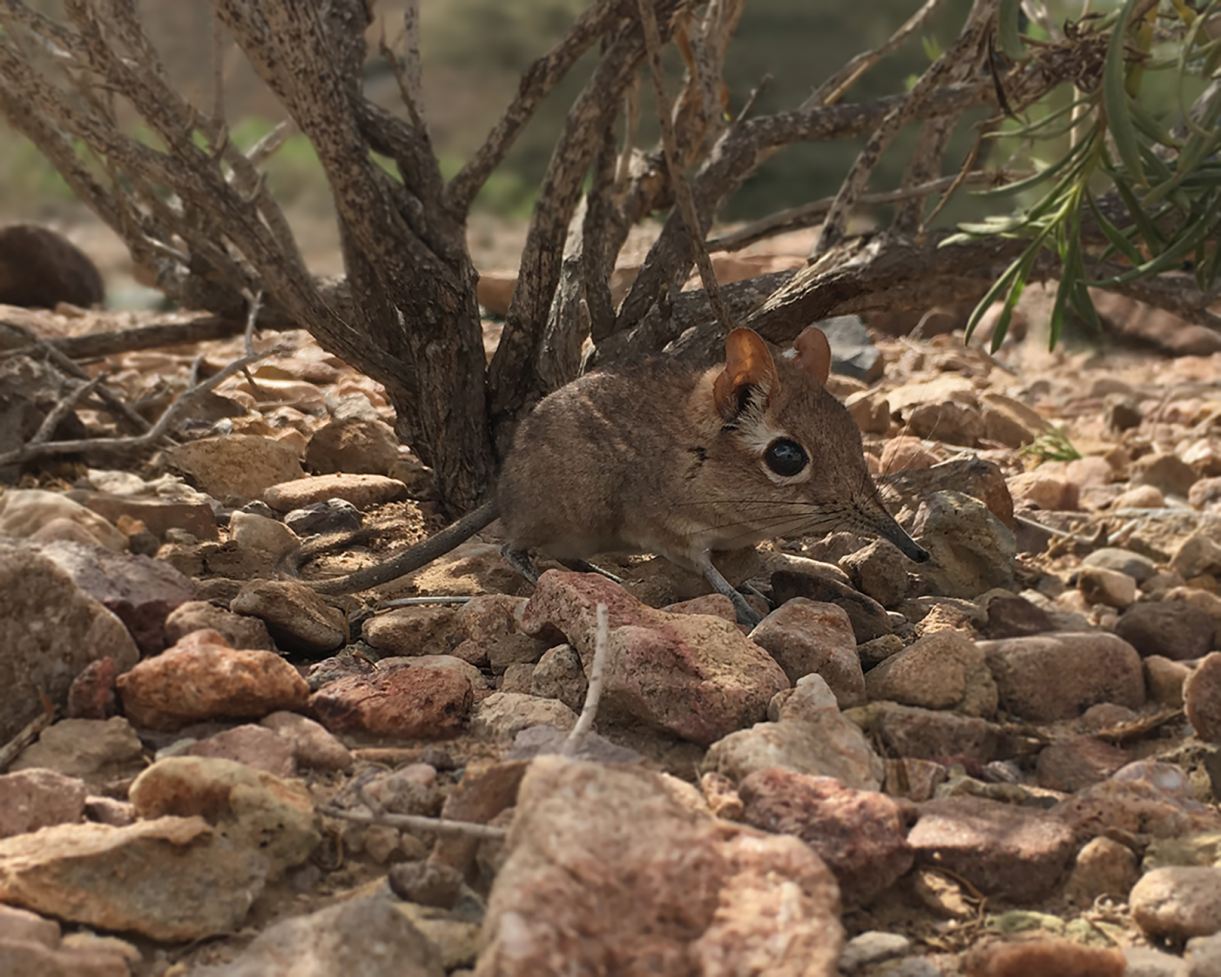
Somali Sengi photograph at the Assamo locality in Djibouti. As is observed in other sengi species, Somali Sengis have some variation in pelage colors. These differences seem to correspond to the color variation of substrates between occurrence localities. At the Assamo site, in the extreme southeastern corner of Djibouti, sengi habitats are comprised of rocks with more rusty coloration than elsewhere in the country. Compared to other Djiboutian sites, sengis from Assamo have dorsal pelage hairs with more pronounced reds. CAS MAM 32728 photograph by Steven Heritage.

**Figure 2:**
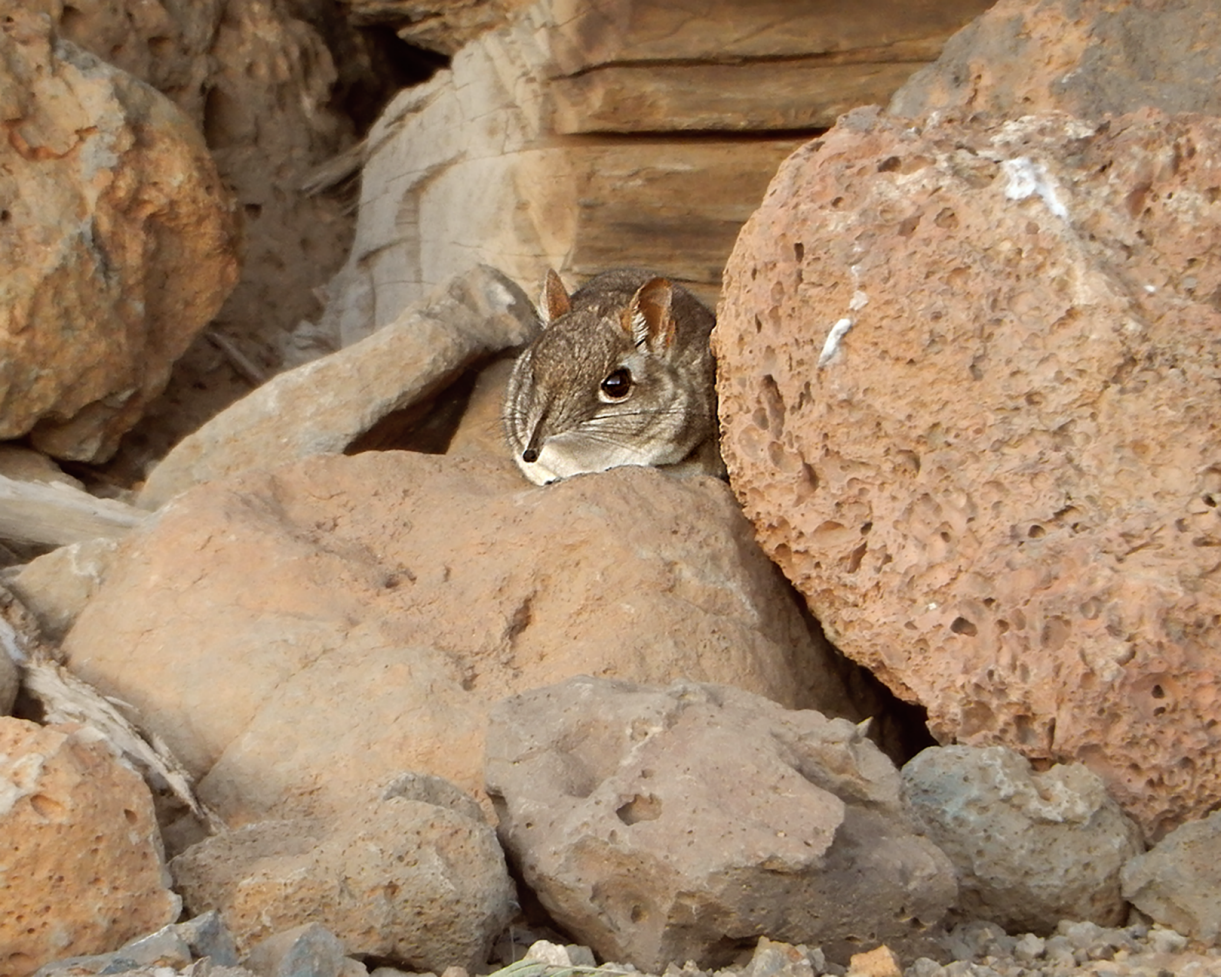
Somali Sengi photograph at the Day Forest locality in Djibouti. One extant sengi species (*Petrosaltator rozeti*) occurs in the Maghreb region but the other nineteen species have ranges restricted to latitudes south of the Sahara (i.e., south of ~13° N). New Somali Sengi records from Djibouti are the northernmost occurrence data for any sub-Saharan macroscelidean taxon and this photographed individual (uncollected) from the Day Forest site documents the northern extent of the Somali Sengi’s known geographic range. This animal was observed sunning itself near a wood pile within the Day Forest Village. Photograph by Houssein Rayaleh.

The 12 targeted trapping sites in Djibouti were from 6 areas (Djalelo, Hemed, Day Forest, Assamo, Grand Bara, and Arta Town) in 4 administrative regions (Arta, Dikhil, Tadjourah, and Ali Sabieh). From the 1,259 traps that were set over 15 dates, we recovered 263 spiny mice (genus *Acomys*), 17 gerbils (genus *Gerbillus*), 1 gundi (genus *Pectinator*), and 8 sengis. Three additional sengis were sighted at Assamo (Fig. 1) and Arta Town and one uncollected sengi was photographed at Day Forest (Fig. 2). Overall small mammal trapping success was about 23% and we note that spiny mice predominance in trap occupancy probably inhibited our targeted approach for sengis.

**Figure 3:**
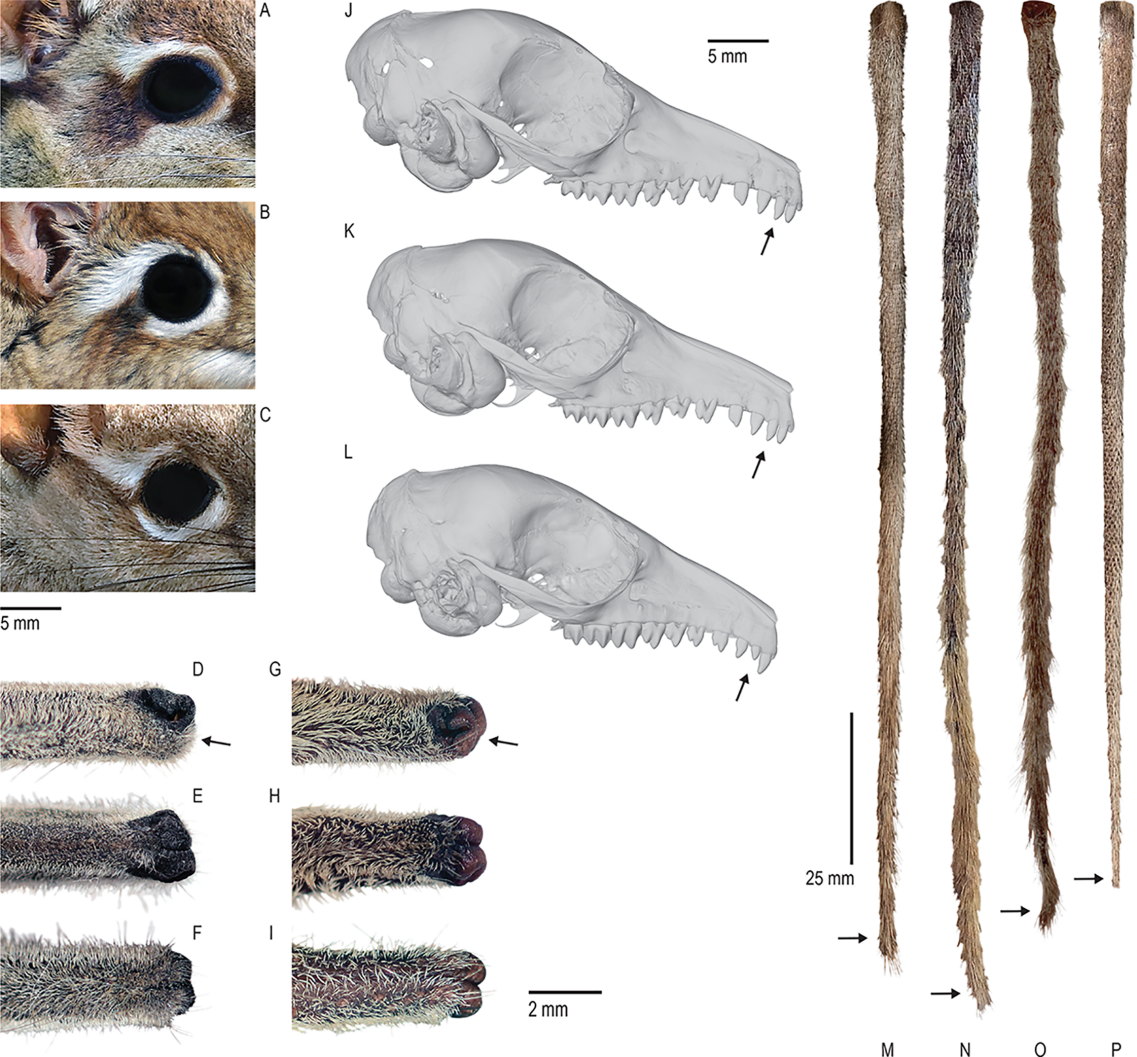
Some distinguishing traits of Somali Sengis. Three sengi species have “spectacled” facial patterns where the eye is surrounded by a pale ring that is interrupted by a dark post-ocular spot. These taxa are the Four-Toed Sengi ((A) *Petrodromus tetradactylus*), Rufous Sengi ((B) *Elephantulus rufescens*), and Somali Sengi ((C) CAS MAM 32728 *revoilii*). From this group, only the Somali Sengi and a single subspecies of Rufous Sengi (*E. rufescens somalicus*) occur in the Horn of Africa. At the distal end of their elongated noses, both the Somali and Rufous Sengis have hair on the inferior portion of the anterior rhinarial skin (CAS MAM 32725 *revoilii* in (D) lateral, (E) dorsal, and (F) ventral views). Other sengi species do not have this trait (AMNH M-31788 *Petrosaltator rozeti* in (G) lateral, (H) dorsal, and (I) ventral views). Somali Sengis have second upper incisors that are about equal in size to the first and third upper incisors ((J) AMNH M-48066 *revoilii* from Somalia; (K) CAS MAM 32723 *revoilii* from Djibouti) but Rufous Sengis have second upper incisors that are smaller than both adjacent teeth ((L) USNM 283315 *E. rufescens*). Additionally, Somali Sengis have relatively hairy tails with hairs that form a tuft at the distal tip ((M) AMNH M-48066 *revoilii* from Somalia in dorsal view; (N) LACM 19499 *revoilii* from Somalia in dorsal view; (O) CAS MAM 32723 *revoilii* from Djibouti in dorsal view). However, Rufous Sengis have tails with sparse and short hairs, exposed skin, and no tufts ((P) USNM 182606 *E. rufescens* in dorsal view). Photographs and micro-CT images by Galen Rathbun and Steven Heritage.

**Figure 4:**
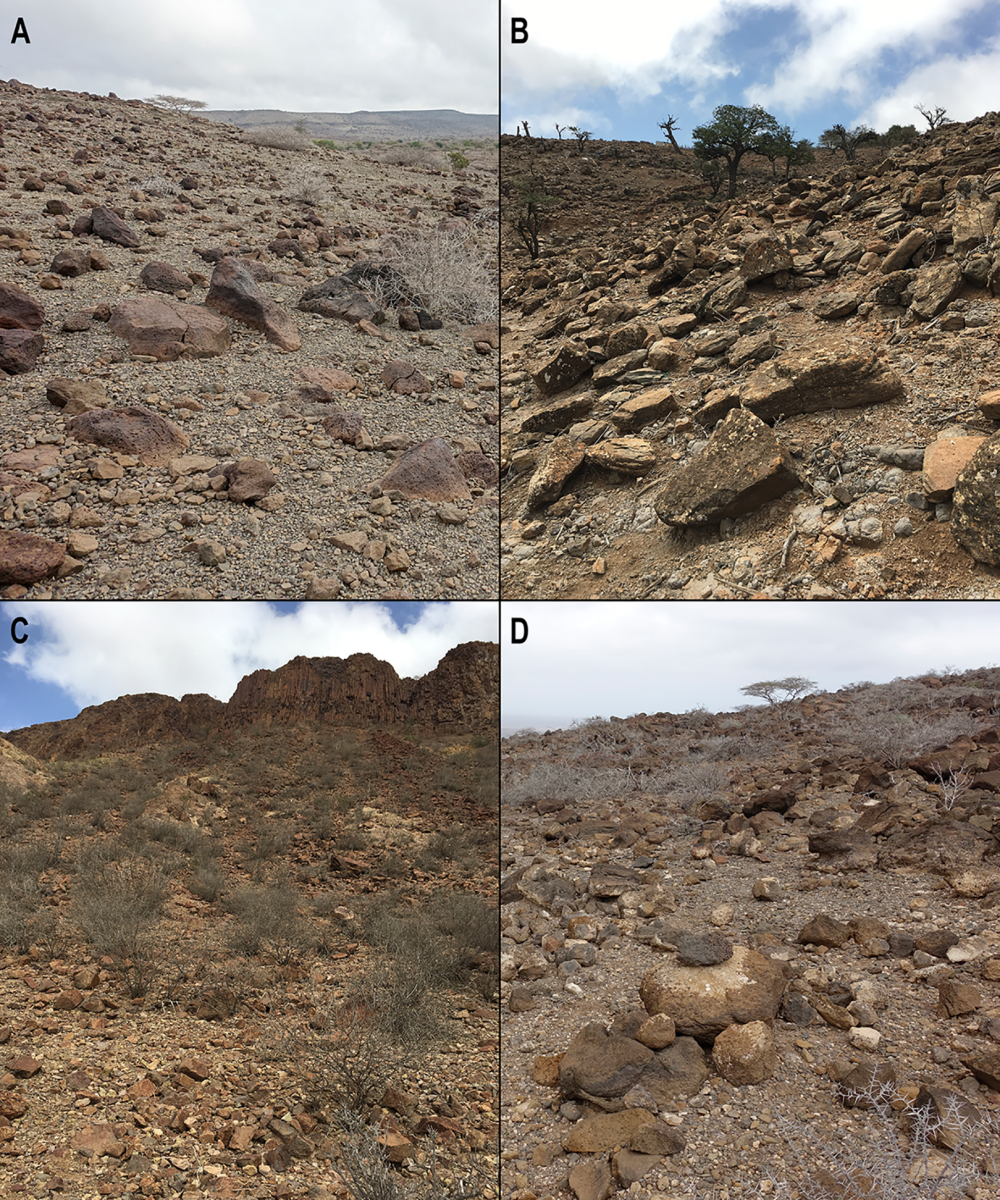
Somali Sengi habitat photographs from four localities in Djibouti. The Somali Sengi has been hypothesized as a rupicolous (petrophilic) species based on the collection coordinates of historic museum specimens which generally occur within the montane ecosystems of northern Somalia. However, whether or not the species is an obligate rock-dweller has not been documented. In Djibouti, all evidence of Somali Sengis are from habitats with rocky substrates and relatively sparse vegetation where the predominant sheltering potential is among boulders. A rupicolous characterization of the Somali Sengi is valid, at least in Djibouti. (A) Arta Region, Djalelo Protected Area, Hansane Hill (east side). (B) Tadjoura Region, Goda Mountain, Day Forest Village (south hill). (C) Ali Sabieh Region, Assamo Decan Camp (south ridge base). (D) Arta Region, Arta Town, Oú Est Mon Camarade Hill (south side). See Table 1 for site coordinates and altitudes. Photographs by Galen Rathbun and Steven Heritage.

All sengis that we documented in Djibouti are without-question ‘*Elephantulus’ revoilii* as diagnosed (Fig. 3) by their spectacled eye patterns, rhinarial hair growth, tail tufts, upper incisor proportions, and relative tail lengths (Corbet & Hanks, 1968). All collection localities were characterized by rocky substrates and relatively sparse vegetation (Fig. 4). We found no evidence of the Somalian Rufous Sengi (smaller bodied, tuftless tail, relative tail length ~100%, relatively small I^2^, pure white feet) in Djibouti, nor did we observe the habitats and substrates that we expect to be used by any subspecies of Rufous Sengi (Koontz & Roeper, 1983; Rathbun, 2009). Our anatomical comparisons of the Djiboutian specimens to historic *revoilii* specimens from Somalia do not lead us to suspect that the *revoilii* sengis in Djibouti are a distinct subspecies.

The new vouchers include 5 males and 3 females. Specimen CAS MAM 32723 was pregnant with a single fetus (11 g) developing from the right uterine horn which we preserved along with the mother. Excluding the fetus, specimens were 41–59 g (mean = 49.9 g) and head-body lengths were 120–138 mm (mean = 128.6 mm). Two specimens had lost portions of their tail tips, but based on the other 6 individuals, average relative tail length was 117%. Additional measurements are reported in Table 1. Pelage colors and patterns are very similar to historic specimens from northern Somalia (e.g., NHMUK 1897.8.9.6, LACM 19499, AMNH M-48066). On the dorsum, hairs are black or dark grey for the basal two-thirds and reddish-brown or yellowish-brown for the terminal one-third. On the flanks, hairs are similarly dark at the base but the tips are slightly lighter than on the dorsum. The ventral pelage appears whitish-grey with hairs that have black or dark grey bases and white termini. As is observed in other sengi species, there is some color variation in the Djiboutian sample which likely corresponds to differences in substrate colors between localities. For instance, specimens from the Assamo site have more pronounced reds in the dorsal and lateral portions of their pelage.

**Figure 5:**
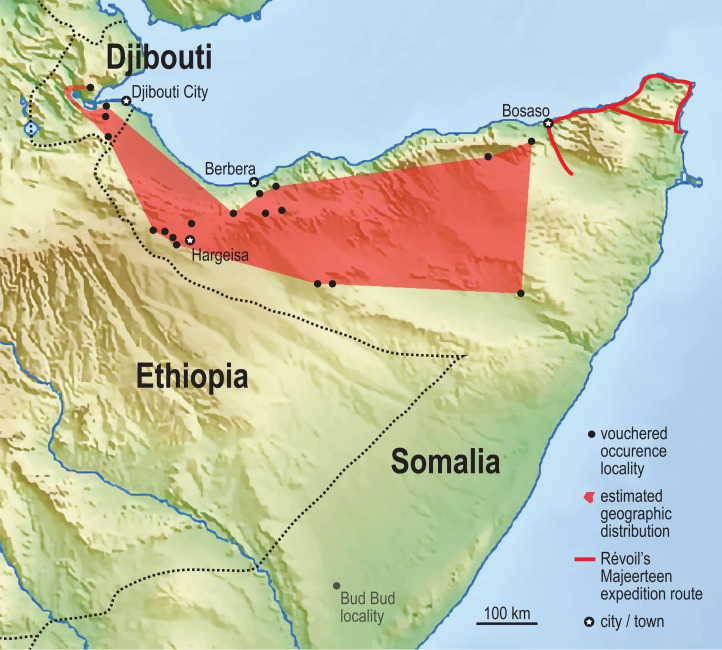
Vouchered occurrence localities of the Somali Sengi. World museum collections preserve Somali Sengi individuals from 15 referenceable sites in northern Somalia (*n* = 35 specimens). The exact site of the holotype specimen (from 1881) is unknown, but Georges Révoil’s documented expedition route in the northern Majeerteen territory indicates that the specimen is from a location to the east of all other records. A minimum convex polygon estimated from these northern Somalia localities is about 88,500 sq km. Inclusion of the new localities from Djibouti increases this area to about 106,000 sq km (polygon in red). See methods for information concerning the Bud Bud site in the eastern branch of Somalia. Base map from Mapswire.com (*CC-BY 4.0*).

The Assamo locality, in the extreme southeastern corner of Djibouti, is 3 km north and 10 km west of the Ethiopian and Somalian borders, respectively. We observed no habitat barriers that would limit the Somali Sengi’s distribution in adjacent areas of these neighboring countries. The Day Forest locality is nearly 100 km to the NNW of Assamo and is the northernmost site where Somali Sengis are evidenced. Given the distance and variability between Assamo and Day Forest, and our observations of the intermediate terrain, we suspect that sengis occur in suitable habitats throughout most of Djibouti. A minimum convex polygon that spans our updated historical occurrence localities in northern Somalia estimates an area of about 88,500 sq km. After combining this locality dataset with the new occurrence sites in Djibouti, the geographic range estimate is increased by 20% to about 106,000 sq km (Fig. 5).

**Figure 6:**
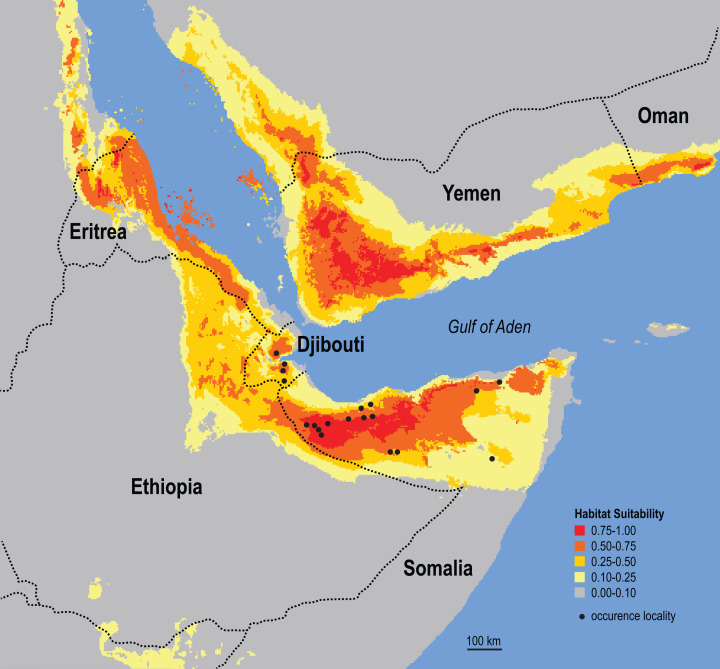
Ecological niche model for the Somali Sengi. Maximum entropy model results are in logistic output format which assigns values to each grid-cell that range from 0 to 1. Values can be interpreted as a measure of habitat suitability for the species, given the occurrence records and environmental variable data used to construct the model. These results predict that suitable habitats for the Somali Sengi are present throughout large portions of northern Somalia and Djibouti—and carry further into other Horn of Africa countries. Although environmental characterization is similar in southern portions of the Arabian Peninsula, sengis have never been documented outside of continental Africa so this prediction may be better interpreted as a discontiguous ecological likeness rather than a potential species distribution. In this analysis, the environmental variables that contributed most to the model were Temperature Seasonality (53.4%), Precipitation of Driest Month (20.5%), and Maximum Temperature of Warmest Month (13.4%). Grid resolution is 2.5 arc-min (4.63 km at the equator).

The results of the ecological niche model indicate that the included environmental variables that best predict the known distribution of Somali Sengis are present throughout large portions of northern Somalia and Djibouti—and carry further into other Horn of Africa countries (Fig. 6). Environmental characterization is similar across the Gulf of Aden in southern portions of the Arabian Peninsula. However, sengis have never been reported outside of the African continent, so this result may be better interpreted as a discontiguous ecological likeness rather than a potential species distribution. Percent contributions of the variables included in the model were: Temperature Seasonality 53.4%, Precipitation of Driest Month 20.5%, and Maximum Temperature of Warmest Month 13.4%. The other variables contributed less than 7% each (see Data S6). The model’s emphasis on a relatively small standard deviation of mean monthly temperatures (WorldClim’s Temperature Seasonality) could be attributable to the coastal proximity (a temperature modulator) of the species’ known range. Otherwise, an interpretation that warm and arid environments are good predictors of the Somali Sengi distribution is consistent with previous discussions (Corbet & Hanks, 1968).

Complete mitogenomic sequences are 16,578 (CASMAM32723), 16,571 (CASMAM32725), and 16,581 (CASMAM32726) base positions (Data S3) and nearly all length variation is contained in D-loop regions. Currently, only two other Macroscelidinae species have published mitogenomes on GenBank which limits detailed comparative analyses at this time. The mitogenomes that are available (*M. proboscideus*, NC_004026, 16,641 bp and *E. edwardii*, NC_041486 16,552 bp) were used here for assembly mapping which yielded alignment gaps (=interspecific length variation) mostly in 12S rRNA, 16S rRNA, and D-loop loci.

**Figure 7:**
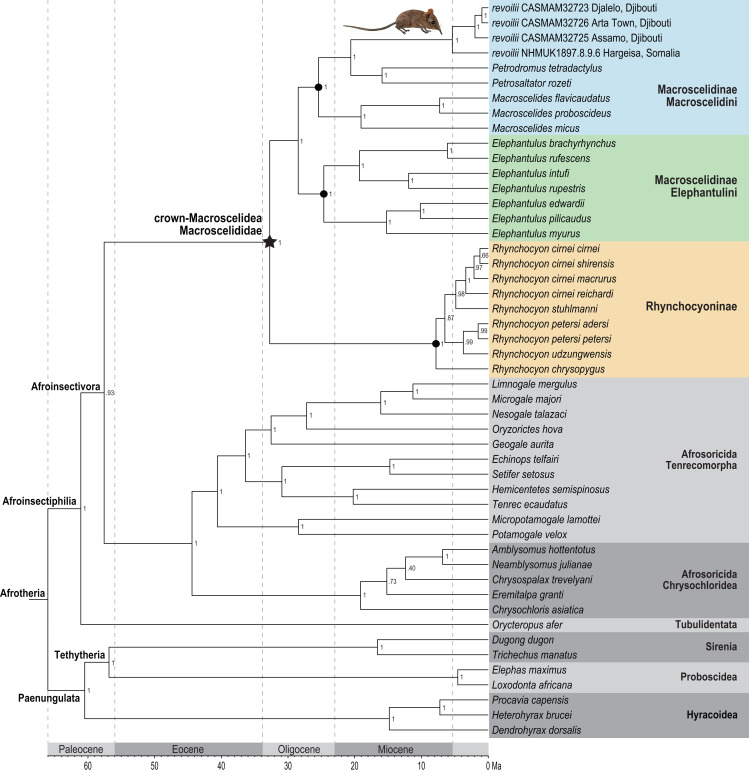
Phylogenetic position of the Somali Sengi. Results from Bayesian phylogenetic analyses of combined nuclear (IRBP, VWF) and mitochondrial (12S, 16S, COX1, CYTB) loci. Clade credibility (=posterior probability) values at tree nodes indicate statistical support for lineage splits given analysis settings. Branch lengths are time-scaled estimates in millions of years (Ma, mega-annum). The Somali Sengi (*revoilii*) group is unambiguously recovered as a descendant of tribe Macroscelidini and sister to the *Petrodromus-Petrosaltator* clade. CAS MAM 32728 photograph by Steven Heritage.

Phylogenetic analysis of the concatenated supermatrix (Fig. 7) found that sengis from the three Djiboutian localities are an exclusive clade which is in turn sister to *revoilii* from northern Somalia. We note that the genetic sequence that represents this Somalian voucher was derived from a specimen (NHMUK 1897.8.9.6) that was collected in the highlands west of Hargeisa in October 1896. Furthermore, phylogenetic placement of this four-specimen *revoilii* group was within tribe Macroscelidini and sister to the *Petrodromus*-*Petrosaltator* clade. Within the subfamily Macroscelidinae, all nodal posterior probability values were unequivocal at 100%. These results do not support a phylogenetic sister-relationship between the Somali Sengi (*revoilii*) and Rufous Sengi (*Elephantulus rufescens*). The common ancestor of the genus *Elephantulus* effectively defines the tribe Elephantulini and our results indicate that the Somali Sengi does not descend from this lineage. All analyses of individual gene segments placed *revoilii* among Macroscelidini taxa (Data S4).

**Figure 8:**
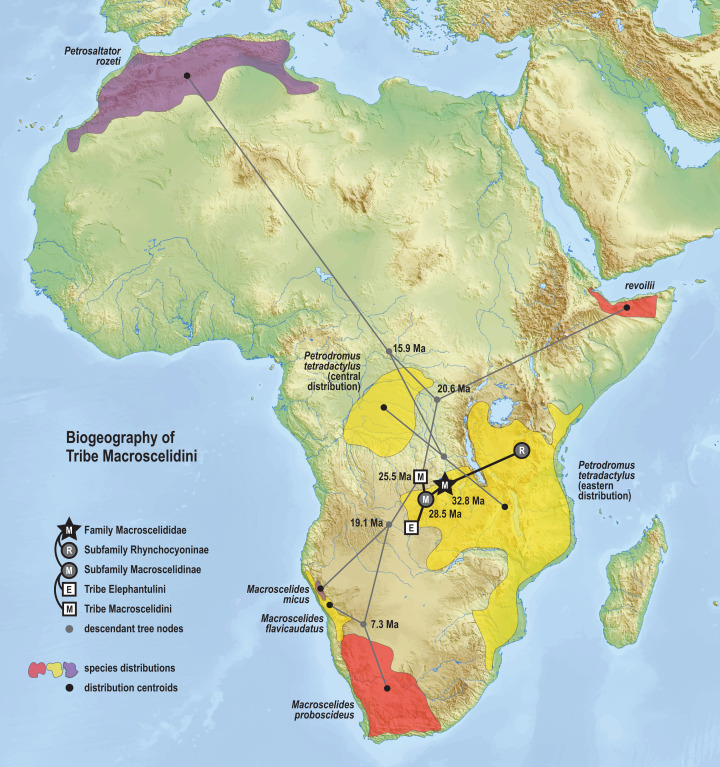
Ancestral biogeography model for tribe Macroscelidini. Subset results (Macroscelidini) from the Bayesian ancestral state reconstruction analysis of sengi biogeography. Continuous variable modeling was used to estimate geocoordinates at each lineage split using the time-scaled phylogenetic result and centroid estimates for species ranges. The spatiotemporal estimate for the common ancestor of tribe Macroscelidini is in Central Africa during the late Oligocene (25.5 Ma, 22.2–29.3 HPD). The arrival of the Somali Sengi in the Horn of Africa must postdate the diversification from its sister clade (early Miocene, 20.6 Ma, 16.8–24.6 HPD) and predate the common ancestor of the four *revoilii* individuals sampled in this study (latest Miocene, 5.4 Ma, 3.1–7.3 HPD). Colors for smoothed distribution polygons were chosen for graphical contrast where species ranges overlap but range colors are otherwise arbitrary. Base map from Mapswire.com (*CC-BY 4.0*).

**Figure 9:**
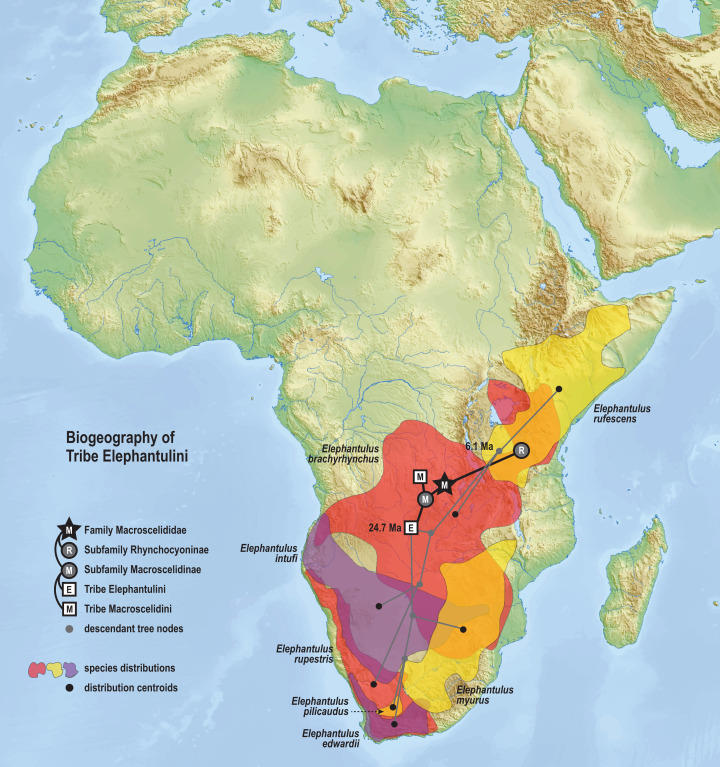
Ancestral biogeography model for tribe Elephantulini. Subset results (Elephantulini) from the Bayesian ancestral state reconstruction analysis of sengi biogeography. The common ancestor of the Rufous Sengi (*Elephantulus rufescens*) and its sister taxon is estimated on the eastern side of the East African Rift (south of Lake Victoria) during the late Miocene (6.1 Ma, 4.3–8.9 HPD). The spread of the Somalian Rufous Sengi (*E. rufescens somalicus*) to the Horn of Africa is thus constrained to a younger age (<6.1 Ma). The common ancestor of tribe Elephantulini is estimated in Central Africa during the late Oligocene (24.7 Ma, 19.0–31.0 HPD). Most extant species that descend from this lineage have geographic distributions predominantly south of the estimated ancestral latitude. Colors for smoothed distribution polygons were chosen for graphical contrast where species ranges overlap but range colors are otherwise arbitrary. Base map from Mapswire.com (*CC-BY 4.0*).

The time-scaled phylogenetic and biogeographic analyses estimate the split of the *revoilii* lineage from the stem leading to the *Petrodromus*-*Petrosaltator* clade with a median age in the early Miocene (20.6 Ma, 16.8–24.6 HPD) and in an area that is now northeastern Democratic Republic of Congo (Fig. 8). The split of the Djiboutian *revoilii* specimens from the single Somalian *revoilii* specimen is estimated in the latest Miocene (5.4 Ma, 3.1–7.3 HPD). Taken together, these dates indicate that the common ancestor of the *revoilii* species (as a whole) must be older than 5.4 million years but younger than the 20.6 Ma lineage diversification. Within the tribe Elephantulini, the split of *Elephantulus rufescens* from its sister taxon is estimated in the late Miocene (6.1 Ma, 4.3–8.9 HPD) in west central Tanzania (Fig. 9). The pattern of phylogenetic descent necessitates that the presence of the Somali Sengi and Somalian Rufous Sengi in the Horn of Africa are independently derived. The chronology within these results constrain the Rufous Sengi’s arrival in northern Somalia to a late Miocene-to-present age and, given this species’ current geographic range, most parsimoniously by a northward route on the eastern side of the East African Rift System. The model also constrains the Somali Sengi’s arrival in the Horn of Africa to an early-to-late Miocene age making this species the presumed earlier inhabitant of the region.

## Discussion

Previous small mammal surveys have not produced vouchered evidence of macroscelideans in Djibouti (Dove et al., 2016; Laurent & Laurent, 2002; Pearch, Bates & Magin, 2001; Rathbun, Agnelli & Innocenti, 2014; Scaramella, Russo & D’Enrico, 1974). However, many interviewees near our trapping sites were familiar with sengis in their country, could accurately discern sengis in a photoset of small mammals, and communicated previous sightings and habitat information. Our targeted approach for macroscelideans yielded results from multiple localities that document, and almost certainly underestimate, a relatively broad geographic range in Djibouti. We found percent trapping success to be equal or better than previous efforts for other Soft-Furred Sengi taxa—for example, *Macroscelides* in Namibia (Dumbacher et al., 2014). Taken together, these results suggest that the Somali Sengi’s comparative species abundance is not rare, at least in Djibouti. The paucity of scientific data concerning the Somali Sengi seems attributable to a gap in targeted research within the Horn of Africa. Regardless, the Somali Sengi should no longer be considered missing, nor is the species endemic to a single country. Our occurrence locality data very near the adjoined borders of Djibouti, Somalia, and Ethiopia strongly suggests that the Somali Sengi is a current inhabitant of all three countries. This is further supported by the results of ENM where suitable habitats for the species are predicted as relatively contiguous in northern Somalia and Djibouti and also carry into Horn of Africa countries where species occurrence has not yet been documented.

Although no Soft-Furred Sengis are currently considered threatened or Near Threatened, conservation concerns for sengi species of the subfamily Rhynchocyoninae are mainly habitat loss and fragmentation associated with growing human populations, agriculture, and livestock grazing (Heritage, 2018; IUCN, 2020). Larger sengi taxa (like *Rhynchocyon* and *Petrodromus*) can also be taken as bushmeat or for trade. In Djibouti, goat herding is unconcentrated but relatively common and certainly occurs in sengi habitats. However, the Somali Sengi is adapted to live and shelter among rocks and the species may tolerate (in its current intensity) the impact of grazing goat herds on vegetation and substrates. Further, given the arid climate and limitation of water sources in Djibouti, agriculture is not (and is unlikely to become) a threat that fragments sengi habitats. We found that the Somali Sengi is present in wildlife protected areas and observed relatively continuous suitable habitats throughout most of the country. Further, it is our understanding that the Djiboutian people do not typically hunt or trap any kind of wildlife for protein (which accords with the cultural traditions of their nomadic lifestyle). We are unable to assess population trend at this time—but there is no reason to believe that numbers are increasing or decreasing significantly. Our recommendation to the IUCN Afrotheria Specialist Group will be that the conservation status of the Somali Sengi be updated from Data Deficient to Least Concern.

One previous study has addressed the Somali Sengi’s phylogenetic position using genetic sequences (Smit et al., 2011). The *revoilii* data in that research was limited by specimen availability and included only ribosomal loci (mitochondrial) derived from an 1800s archival voucher. The result was a spurious placement at the base of subfamily Macroscelidinae (outside of any monophyletic genus), relatively low statistical support, and a comment by the authors that *revoilii*’s position remains unresolved. Our approach differed by representing *revoilii* with multiple nuclear and mitochondrial loci, by incorporating independently parameterized per partition substitution models, in alignment strategy, in broader outgroup sampling, and in the application of multiple age calibrations for the estimation of time-scaled branch lengths. The resultant placement of all *revoilii* specimens, including the historic specimen from Somalia, was with maximum statistical support.

As a descendant of the Macroscelidini lineage, and as the immediate sister taxon to the *Petrodromus–Petrosaltator* clade, the Somali Sengi’s taxonomic referral to the genus *Elephantulus* is incompatible with macroscelidean phylogeny. The diversification split of the *revoilii* stem from its sister clade predates the age estimates for the common ancestors of four of the extant sengi genera and thus signals a comparatively long period of lineage independence. Further, *revoilii* cannot be reassigned to any existing genus without first collapsing together *Petrodromus* and *Petrosaltator*. These two genera are well-established as distinct (Corbet & Hanks, 1968; Dumbacher, Carlen & Rathbun, 2016) based on morphology, genetics, ecology, biogeography, and (by our estimate) 15.9 million years since their shared common ancestor. This extraordinary taxonomic revision cannot be justified. Instead, we accommodate the resolved phylogenetic position of the Somali Sengi—previously *Elephantulus revoilii—*by recognizing a new genus replacement:

***Galegeeska* Heritage & Rayaleh 2020, new genus**

urn:lsid:zoobank.org:act:68FACC58-A04D-44E5-AA86-30FB87B86611

**Type species**: *Galegeeska revoilii* (*Hüet, 1881*). New binomial combination. The genus is currently monotypic.

**Species Synonyms**: *Elephantulus revoilii*, *Elephantulus revoili*, *Macroscelides revoilii*

**Holotype**: The type specimen, MNHN ZM-MO-1881-11, is located at the National Museum of Natural History in Paris, France. See Data S5 for hypodigm voucher specimens.

**Distribution**: The documented geographic range includes Somalia and Djibouti. The species may also occur in other Horn of Africa countries.

**Diagnosis and description**: See Corbet & Hanks (1968), Hüet (1881), and elsewhere in this manuscript for full details. In brief, the Somali Sengi can be distinguished from all other macroscelidean species by a combination of traits including: the presence of a pale eye ring with a dark post-ocular mark, hair on the lower portion of the rhinarium, a tufted tail, and second upper incisors that are subequal in size to adjacent upper incisors.

**Etymology**: In the Somali language, the word *geeska* ([**gey**·skah], /'gāskä/) means *corner* or *horn—*as in Geeska Afrika (=Horn of Africa). This root is chosen to indicate that the species has a broader geographic range than was previously recognized. From the Ancient Greek, *galê* translates to *weasel* (Beekes, 2009) and has been widely used as a general descriptor for small mammal taxa (e.g., *Galeopterus*, *Petrogale*, *Microgale*). In Somali, *gal* (variants: *gala*, *galay, gale*) can refer to *one who enters or inhabits*. A direct translation of *Galegeeska* is “weasel of the horn”, but a Somali speaker’s interpretation could be “inhabitant of the horn”. The root *gale* is also chosen to honor Galen B. Rathbun (1944–2019), who devoted more than four decades of scientific inquiry to sengi biology and who hiked the Djiboutian hillsides in search of this species.

The genus-level taxonomic hierarchy for extant sengis is as follows:

Class: Mammalia *Linnaeus, 1758*

Supercohort: Afrotheria *Stanhope et al., 1998*

Order: Macroscelidea *Butler, 1956*

Family: Macroscelididae *Bonaparte, 1838*

Subfamily: Rhynchocyoninae *Gill, 1872*

Genus: *Rhynchocyon* *Peters, 1847*

Subfamily: Macroscelidinae *Bonaparte, 1838*

Tribe: Elephantulini *Dumbacher, Carlen & Rathbun, 2016*

Genus: *Elephantulus* *Thomas & Schwann, 1906*

Tribe: Macroscelidini *Bonaparte, 1838*

Genus: *Macroscelides* *A. Smith, 1829*

Genus: *Petrodromus* *Peters, 1846*

Genus: *Petrosaltator* *Dumbacher, Carlen & Rathbun, 2016*

Genus: *Galegeeska* *Heritage et al., 2020*, **new genus**

We suggest that the existing common name, Somali Sengi, remains in use. This retention will continue to acknowledge that the taxon’s first description in the scientific literature was based on a specimen from Somalia and also provides a continuity of terminology before and after the generic rank reattribution. Concerning the spelling of the specific epithet, the ICZN code is clear in Articles 32.2.1, 32.3, 32.5.1 and 33.4 that the original designation *revoilii* is the only valid spelling. Unfortunately, the shortened version ‘*revoili*’ has entered the literature and this misspelling has become a legacy. Several species of plants and animals were first described after Revoil’s expedition returned from Somalia, and all that were named for him used -*ii* as the genitive of his Latinized surname.

Some historic Somalian specimens are accompanied by collection notes which indicate that *walo* (variant: *wallo*) was the local name for sengis at the time. We note that *walo* is currently recognized as a common name for the Somali Gerbil (*Ammodillus imbellis*) (Granjon, Gerrie & Kennerley, 2017) and may also be a modern local reference for other rodents and mouse-sized mammals. The local name w*alo sandheer*, where sandheer translates to long-nose, is also a current reference for macroscelideans in Somalia. In Djibouti, we found that the vernacular for sengis is *wali* or *wali sandheer* and that local people distinguish *wali* from *walo* (e.g., spiny mice).

DNA sequences are the primary support for grouping the species of tribe Macroscelidini. Discrete anatomical traits that are shared among this clade have been notoriously elusive and thus remain a subject of active research. However, a few characters have been proposed as common to some Macroscelidini taxa. (1) In both *Petrodromus* and *Petrosaltator*, males possess size-reduced nipples (relative to females) but males lack nipples in all other sengi species (Olbricht & Stanley, 2009), including the Somali Sengi. (2) A similarly shaped glans penis with bilateral lobes and a tapered tip has been reported for *Petrodromus* and *Petrosaltator* (Woodall, 1995); though there is some shape variation between these genera and also among *Petrodromus*. The collared shape of the *Macroscelides* glans clearly differs from these taxa. Comparative analysis of the Somali Sengi glans will be a forthcoming project. (3) Details of the Facial Nerve (CN VII) pathway—as the nerve courses through the tympanic cavity and exits the stylomastoid opening—have also been proposed as a shared trait. In extant sengis, this pathway can be characterized as two dichotomous states (Benoit et al., 2014; Benoit, Orliac & Tabuce, 2013). The nerve either runs in a groove along the petrosal bone between the secondary facial foramen and the stylomastoid foramen/notch (i.e., facial sulcus or stapediofacial groove) or a fully ossified tube encloses the nerve’s path along the petrosal and also contains a segment of the stapedial artery (i.e., stapediofacial tube). Both *Macroscelides* and *Petrosaltator* possess the ossified tube. However, the groove is present in *Petrodromus* and this state has been clearly discussed and figured (Benoit et al., 2014). A groove is also present in the Somali Sengi. The suggestion that the presence of a stapediofacial tube is a non-homoplastic character state shared by *Macroscelides, Petrosaltator* and *Petrodromus* is in error and has unfortunately been cited as non-molecular character support for the *Petrodromus–Petrosaltator* clade (Dumbacher, Carlen & Rathbun, 2016). (4) A 2*n* = 28 karyotype is shared by *Petrodromus* and *Petrosaltator* which contrasts with the 2*n* = 26 chromosome complement of *Macroscelides* and most species in tribe Elephantulini (Smit et al., 2011). The karyotype of the Somali Sengi has not yet been characterized. (5) The upper third incisors (I^3^) of *Petrodromus* are patently double-rooted. Some authors have proposed the presence of slightly-developed double roots on I^3^ of *Petrosaltator* (Corbet & Hanks, 1968) and it has been suggested that double roots could be a shared character of the two taxa (Dumbacher, Carlen & Rathbun, 2016). Our assessment of micro-CT scanned *Petrosaltator* specimens (e.g., AMNH M-31788 and FMNH 47754) finds this tooth to be single-rooted. However, we acknowledge that the root has a relatively wide (or fin-shaped) terminus and does not taper to a cone-like tip. The alveolar bone at the I^3^ crown-base reveals no mid-tooth constriction and there is a single foramen in the root tip for the transmission of the alveolar nerve and vessels. These conditions support a single-rooted interpretation. The only hint of root bifurcation are the two nubs at the mesial and distal edges of the root terminus. The I^3^ of Somali Sengis also has a fin-shaped single root but in *Macroscelides* the single root is tapered. (6) One study has assessed sengi ventral crania with two-dimensional geometric morphometrics and applied neighbor joining clustering to the Procrustes distance matrix where the Somali Sengi was placed as nested among *Petrodromus*, *Petrosaltator* and *Macroscelides* (Scalici & Panchetti, 2011). The shape of the ventral cranium is, to our knowledge, the only published morphological evidence that unites all taxa of the monophyletic Macroscelidini tribe. The phylogenetic position of the Somali Sengi, as signaled by DNA sequences, thus has precedent in the phylogenetic hypothesis from shape analysis.

The Djiboutian localities reported here have some habitat variability and thus provide details about the environmental conditions that can support the Somali Sengi species. The Assamo site is characterized as BWh (hot arid desert) in the Köppen-Geiger climate classification system with about 200 mm annual precipitation and an average monthly temperature (AMT) range of 21.1–31.6 °C throughout a given year. Principal flora at Assamo include *Cadaba rotundifolia*, *Balanites rotundifolia*, and *Boscia coriacea*. By contrast, the Day Forest site at Goda Mountain is classified as BSh (hot semi-arid steppe) with a per year AMT range of 16.4–25.9 °C and 430 mm annual precipitation. *Juniperus procera*, *Olea africana*, and *Acacia etbaica* are among the main vegetation at Day Forest and these species occur in relatively dispersed distributions along rocky hillsides and valleys. The Arta region, which includes both the Djalelo site and the site near Arta Town, is also classified as BWh with about 230 mm annual precipitation and an AMT range of 20.0–32.3 °C. *Acacia mellifera* and *Rhigozum somalense* are common at the Arta Town locality while *Acacia horrida* and *Balanites rotundifolia* are among the principal flora at Djalelo. Throughout Djibouti, annual temperature peaks are between 40 °C and 50 °C (Association Djibouti Nature, 2019, unpublished data). Given these mostly arid climates, Somali Sengis almost certainly consume most of their water in food. Additionally, while we can confirm sheltering among boulders, Somali Sengis could also take cover under suitable low shrubs if present. However, low shrubs can be sparse or absent at some sites.

**Figure 10:**
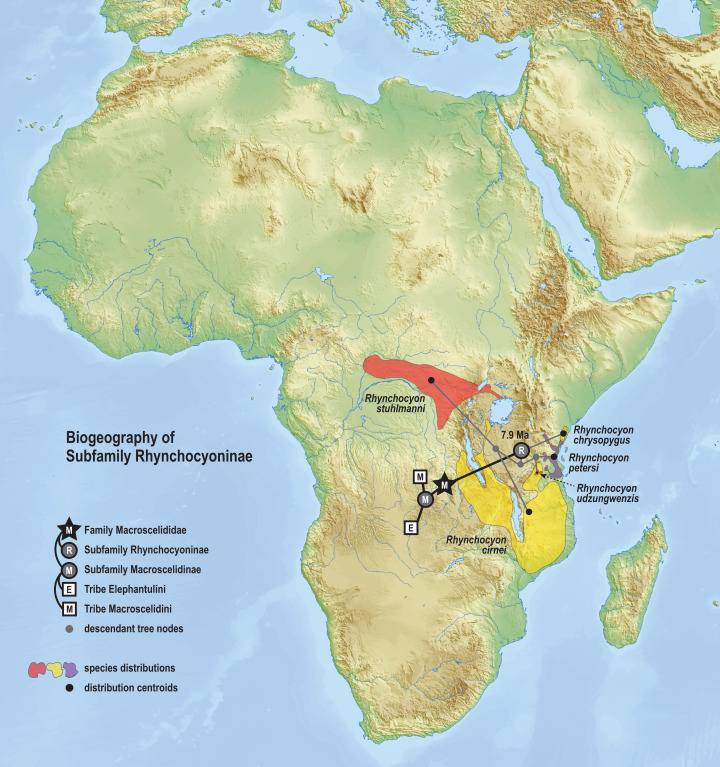
Ancestral biogeography model for subfamily Rhynchocyoninae. Subset results (Rhynchocyoninae) from the Bayesian ancestral state reconstruction analysis of sengi biogeography. Commonly called Giant Sengis, the extant taxa that comprise the subfamily Rhynchocyoninae have an estimated origin in East Africa during the late Miocene (7.9 Ma, 5.2–11.3 HPD). This age is also associated with a reduction in canopy forests and expansion of C4 grasslands in Africa. The coincidence is noteworthy because Giant Sengis require habitats with canopies that produce dense leaf litter. A relatively young crown-clade ancestor and a relatively long stem-lineage (32.8–7.9 Ma) implies that fossil Giant Sengi taxa that predate the early Miocene were derived from the Rhynchocyoninae stem. The spread of the extant subfamily’s range into Central Africa is constrained to a Pliocene or younger age (<4.9 Ma). Colors for smoothed distribution polygons were chosen for graphical contrast where species ranges overlap but range colors are otherwise arbitrary. Base map from Mapswire.com (*CC-BY 4.0*).

The phylogenetic model of ancestral biogeography presented here generates new hypotheses about the geographic origins of sengi clades. The biogeographic patterns of tribe Macroscelidini (Fig. 8) are remarkable with an estimated common ancestor in Central Africa during the late Oligocene (25.5 Ma, 22.2–29.3 HPD) and extant descendants widely dispersed across the continent. We note that all sengi species are non-migratory and that individuals spend most of their adult lives within a single home range that is <0.1 sq km (Rathbun & Dumbacher, 2015). Nevertheless, from the tribe’s origin, the *Macroscelides* lineage has spread to Africa’s southern cape, the *Petrosaltator* lineage has spread to the Maghreb region of North Africa, and *Petrodromus* subspecies are present in both Central and East Africa. The recognition of the Somali Sengi as a Macroscelidini taxon appends the tribe’s grand biogeographic story with a spread into the Horn of Africa. Our time estimate for the *Petrodromus*-*Petrosaltator* split slightly predates previous estimates (Douady et al., 2003; Smit et al., 2011) and thus should not constrain prior hypotheses about the role of Sahara aridification in the North African distribution of *Petrosaltator* (Douady et al., 2003; Nyári, Peterson & Rathbun, 2010). Macroscelidini and Elephantulini may have had similar temporospatial origins, but for Elephantulini (Fig. 9), most extant species have geographic distributions to the south of the tribe’s estimated ancestral latitude and overlapping species ranges are comparatively extensive. Given the distribution of all extant *Rhynchocyon* species, an East African origin of crown-clade Rhynchocyoninae (Fig. 10) is not unexpected. However, the age estimate (late Miocene, 7.9 Ma, 5.2–11.3 HPD) for this common ancestor is noteworthy. The late Miocene is associated with a reduction in closed canopy forests and the expansion of open landscapes and C4 grasslands in East Africa (Ségalen, Lee-Thorp & Cerling, 2007). *Rhynchocyon* species require habitats with dense leaf litter (i.e., canopy forests and dense woodlands) which is used to build nests for neonates and for nightly sheltering (Rathbun, 1979, 2009). Extinct Rhynchocyoninae taxa that predate the crown-clade could have been pressured by expanding grasslands and the spread of the subfamily’s geographic range could have been, and is, constrained by forest contiguity. Further, the late Miocene estimate for crown-clade Rhynchocyoninae is quite young (cf. Macroscelidinae) and supports stem-positions for early Miocene fossils that are referred to the subfamily (Holroyd, 2010; Novacek, 1984).

Foot drumming is a communication behavior of many species of Soft-Furred Sengis where one or both hindfeet are rapidly and audibly tapped on the ground. In Djibouti, we observed the Somali Sengi engage in foot drumming when approached for close photography. A short video clip of this behavior is provided in Data S8. Sengi foot drumming has been noted in several settings with proposed functions that include intrasexual signals about home range boundaries, mate communication, predator alerts, conspecific recognition, signaling a predator of the intent to flee, and other types of intraspecific and interspecific communications (Faurie, Dempster & Perrin, 1996; Randall, 2015; Rathbun, 1979; Roeper, 1981). It may be the case that sengi foot drumming has several functions. Regardless, multiple Macroscelidini and Elephantulini species have been observed foot drumming which suggests that this behavior had already evolved and was present in the common ancestor of the Macroscelidinae subfamily (early Oligocene, 28.5 Ma, 23.2–34.3 HPD).

The phylogenetic systematics and taxonomy of extant sengis have been relatively active research topics and in the past two decades have included: field discoveries of new species (Dumbacher et al., 2014; Rovero et al., 2008); the detection of unrecognized taxa in museum collections (Smit et al., 2008); the elevation of subspecies to species-level ranks (Carlen et al., 2017; Dumbacher et al., 2012); and molecular discoveries of unsuspected descent patterns which have prompted taxonomic revisions (Douady et al., 2003; Dumbacher, Carlen & Rathbun, 2016). We consider the phylogenetic positions of *Elephantulus fuscus* and *Elephantulus fuscipes* to be unresolved and note that these two species were not included in our analysis. Similar to the Somali Sengi, a molecular assessment of both *E. fuscus* and *E. fuscipes* has been somewhat problematic with challenges in obtaining modern voucher tissues and in the limited hypodigm of historic museum specimens (Rathbun, 2015a, 2015b). Previous DNA sequencing of these species has produced partial mitochondrial rRNA segments but no amino acid coding loci (Smit et al., 2011) and these limited data have yielded seemingly anomalous phylogenetic estimates, much like the Somali Sengi. We advocate that more evidence is needed to produce compelling phylogenetic hypotheses for *E. fuscus* and *E. fuscipes* and consider this is an important pending issue in sengi systematics. Given these and other remaining questions (e.g., the unknown affinities among *Petrodromus* subspecies) and the recent activity in macroscelidean research, we expect that the understanding of sengi phylogeny will continue to evolve.

## Conclusion

These new records confirm the Somali Sengi as an extant taxon and reveal a broader geographic distribution than was previously documented. Further, the species’ conservation status can now be reconsidered using modern evidence which we have discussed as positively informing assessment criteria. New data also characterizes habitat use (including substrate and sheltering affiliations)—a species attribute that has been hypothesized but never formally reported. DNA from voucher specimens have yielded a robust phylogenetic estimate that newly identifies the Somali Sengi as a descendant of the Macroscelidini lineage. The former taxonomic referral to the genus *Elephantulus* (and tribe Elephantulini) has been revised and the Somali Sengi species-group is now recognized as *Galegeeska revoilii*. Future fieldwork in the Horn of Africa should aim to further document extent of occurrence, to monitor population trends, and to study the behavioral ecology and life history of the species.

## Supplemental Information

10.7717/peerj.9652/supp-1 Supplemental Information 1 PCR Primers, Annealing Temperatures, and Extension Times.

10.7717/peerj.9652/supp-2 Supplemental Information 2 GenBank Accession Numbers and Source Specimen Information.

10.7717/peerj.9652/supp-3 Supplemental Information 3 DNA Alignments and Phylogenetic Analyses.

10.7717/peerj.9652/supp-4 Supplemental Information 4 Phylogenetic Analyses of Individual Loci.

10.7717/peerj.9652/supp-5 Supplemental Information 5 Somali Sengi Museum and Geocoordinates Data.

10.7717/peerj.9652/supp-6 Supplemental Information 6 Ecological Niche Modeling Results.

10.7717/peerj.9652/supp-7 Supplemental Information 7 Ancestral Biogeography Analyses Data and Settings.

10.7717/peerj.9652/supp-8 Supplemental Information 8 Somali Sengi Foot Drumming.

## Additional Information and Declarations

### Field Study Permissions

The following information was supplied relating to field study approvals (i.e., approving body and any reference numbers): Fieldwork, collection, and export authorizations were granted by the Department of Environment and Sustainable Development, Ministry of Urban Affairs, Environment and Tourism, Republic of Djibouti (Document 619|DEDD|18).

### DNA Deposition

The following information was supplied regarding the deposition of DNA sequences: Accession numbers and Source Specimen Information are available in the Supplemental Files. The group sequences described are available via GenBank accession numbers: MT354760–MT354768.

### Data Availability

The following information was supplied regarding data availability: Digital data are available in the Supplemental Files. Voucher specimens and tissues are deposited at and available from the California Academy of Sciences, Department or Ornithology and Mammalology. Catalog numbers of the 8 specimens are: CAS MAM 32723; CAS MAM 32724; CAS MAM 32725; CAS MAM 32728; CAS MAM 32726; CAS MAM 32729; CAS MAM 32727; CAS MAM 32730. The CAS mammal collections database is here: http://researcharchive.calacademy.org/research/bmammals/MamColl/index.asp.

### New Species Registration

The following information was supplied regarding the registration of a newly described species: Publication LSID: urn:lsid:zoobank.org:pub:7D882E47-0982-46E7-AA67-20BB71FDD31D. *Galegeeska* gen. nov. urn:lsid:zoobank.org:act:68FACC58-A04D-44E5-AA86-30FB87B86611.
