# Supplementary material for: New records of a lost species and a geographic range expansion for sengis in the Horn of Africa
Source: PeerJ. 2020 Aug 18;8:e9652. doi: 10.7717/peerj.9652 (PMC7441985; doi:10.7717/peerj.9652)
Supplement: Supplemental Information 3 [file peerj-08-9652-s003.zip › Data_S3_DNA_Alignments_and_Phylogenetic_Analysis/2_Phylogenetic_Analysis/2_MrBayes/2_MrBayes_results/Heritage.et.al.2020_Sengis_Bayesian_Phylogenetic_Analaysis_STANDARD.pdf]

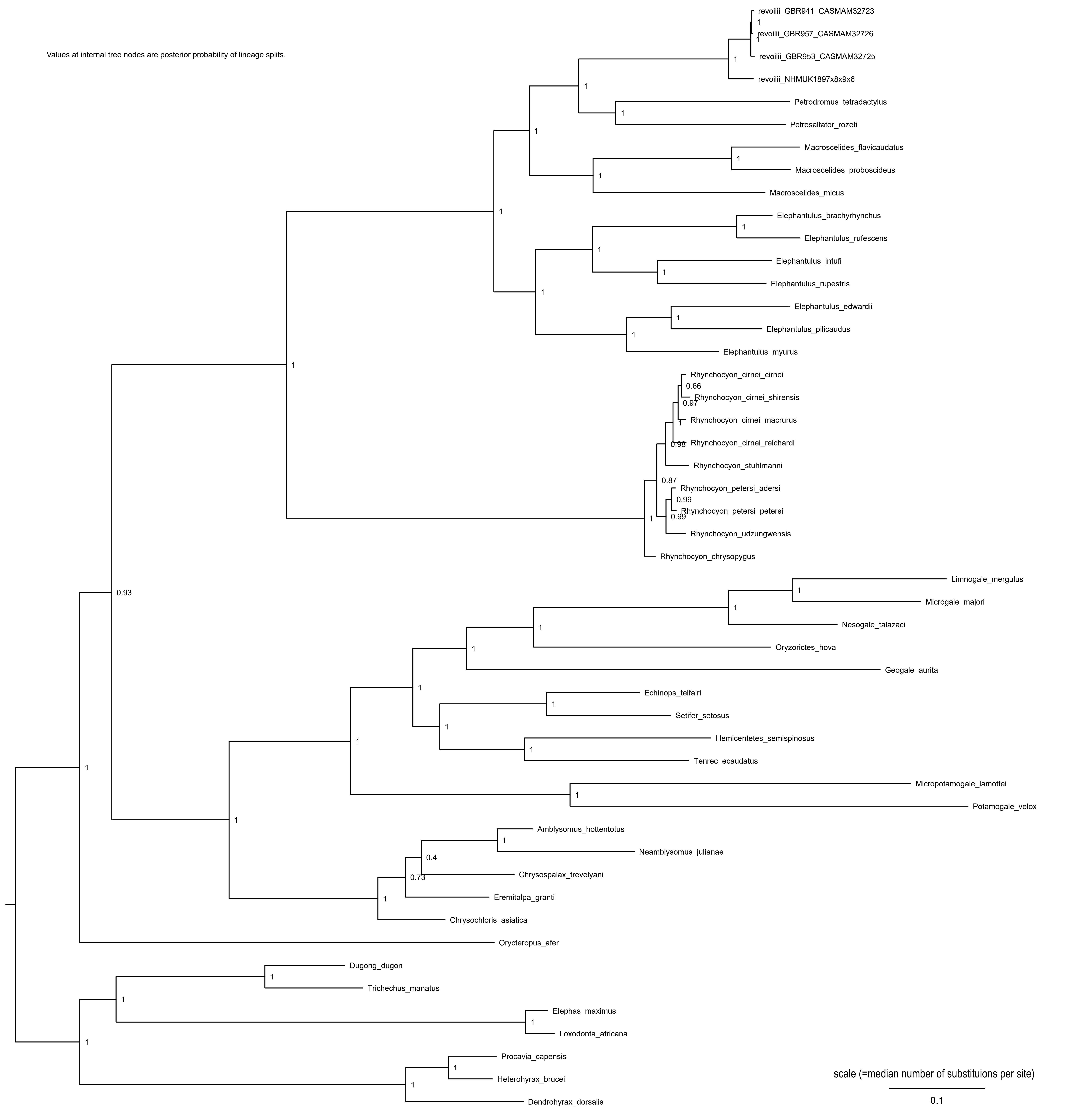

Genetic distance matrix reporting percent identity of macroscelidean DNA sequences using the six-locus supermatirx alignment.

	revoilii_GBR941_CASMAM32723	revoilii_GBR953_CASMAM32725	revoilii_GBR957_CASMAM32726	revoilii_NHMUK1897x8x9x6	Petrodromus tetradactylus	Petrosaltator rozeti	Macroscelides micus	Macroscelides flavicaudatus	Macroscelides proboscideus	Elephantulus brachyrhynchus	Elephantulus edwardii	Elephantulus intufi	Elephantulus myurus	Elephantulus pilicaudus	Elephantulus rufescens	Elephantulus rupestris	Rhynchocyon chrysopygus	Rhynchocyon cirnei cirnei	Rhynchocyon cirnei macrurus	Rhynchocyon cirnei reichardi	Rhynchocyon cirnei shirensis	Rhynchocyon petersi adersi	Rhynchocyon petersi petersi	Rhynchocyon stuhlmanni	Rhynchocyon udzungwensis
revoilii_GBR941_CASMAM32723	100.00																								
revoilii_GBR953_CASMAM32725	99.41	100.00																							
revoilii_GBR957_CASMAM32726	99.77	99.49	100.00																						
revoilii_NHMUK1897x8x9x6	88.66	88.77	88.72	100.00																					
Petrodromus tetradactylus	65.05	64.95	65.09	83.26	100.00																				
Petrosaltator rozeti	64.85	64.74	64.88	83.64	86.78	100.00																			
Macroscelides micus	65.23	65.09	65.23	81.82	84.36	88.10	100.00																		
Macroscelides flavicaudatus	64.66	64.63	64.70	81.75	85.01	88.38	89.97	100.00																	
Macroscelides proboscideus	83.56	83.46	83.57	81.92	64.59	67.23	69.15	72.31	100.00																
Elephantulus brachyrhynchus	52.67	52.60	52.71	88.98	76.65	72.24	71.32	71.60	51.24	100.00															
Elephantulus edwardii	55.47	55.40	55.41	80.48	64.23	64.68	65.68	64.90	50.62	55.69	100.00														
Elephantulus intufi	60.72	60.61	60.75	84.12	84.97	80.83	80.17	80.52	61.03	82.03	63.02	100.00													
Elephantulus myurus	53.68	53.55	53.71	81.71	72.07	72.27	73.53	72.95	56.25	58.21	54.34	69.25	100.00												
Elephantulus pilicaudus	47.99	47.97	47.97	88.15	73.10	67.43	66.13	66.57	44.96	84.47	75.09	77.25	50.75	100.00											
Elephantulus rufescens	66.10	66.04	66.10	82.28	80.14	82.50	83.58	83.29	64.36	77.77	68.75	79.23	71.20	67.77	100.00										
Elephantulus rupestris	62.22	62.09	62.24	82.97	85.12	81.57	80.71	80.84	61.82	76.89	63.78	85.20	70.73	75.34	79.05	100.00									
Rhynchocyon chrysopygus	30.11	30.01	30.17	53.02	42.27	42.48	43.15	42.68	30.98	44.59	22.52	43.71	56.53	34.02	41.86	42.47	100.00								
Rhynchocyon cirnei cirnei	50.56	50.47	50.64	78.09	67.66	67.85	68.65	68.19	52.34	54.02	49.26	64.05	84.15	46.43	65.27	65.53	65.84	100.00							
Rhynchocyon cirnei macrurus	51.27	51.16	51.34	78.14	64.92	64.93	66.29	65.40	50.98	53.34	50.26	61.47	82.23	46.50	66.78	62.77	64.54	94.73	100.00						
Rhynchocyon cirnei reichardi	50.32	50.23	50.40	77.81	67.76	67.81	68.75	68.11	52.27	54.15	49.18	64.00	84.22	46.47	65.30	65.57	65.68	99.39	94.45	100.00					
Rhynchocyon cirnei shirensis	62.14	62.00	62.14	64.02	58.64	59.61	61.93	60.21	60.97	64.26	60.87	61.26	62.28	64.86	61.89	60.31	92.70	72.78	72.71	72.23	100.00				
Rhynchocyon petersi adersi	65.79	65.66	65.79	67.72	62.46	63.50	65.72	64.34	65.24	68.47	64.60	65.12	66.21	68.77	65.82	63.84	97.98	75.88	75.81	75.53	93.46	100.00			
Rhynchocyon petersi petersi	57.56	57.44	57.63	78.14	59.61	59.77	60.51	60.15	59.91	46.46	44.30	57.11	72.35	38.44	58.37	57.69	53.96	85.21	81.47	84.95	71.81	76.71	100.00		
Rhynchocyon stuhlmanni	50.13	50.06	50.22	78.69	66.87	67.06	68.19	67.30	51.91	54.75	49.07	64.45	83.55	47.03	64.92	64.85	66.68	97.59	93.34	97.54	72.51	75.85	83.62	100.00	
Rhynchocyon udzungwensis	61.34	61.24	61.39	77.70	78.58	82.44	82.93	82.71	63.23	68.30	62.57	75.02	68.47	64.39	79.18	75.45	50.21	80.48	77.17	80.32	71.26	75.60	71.32	79.13	100.00
