# Supplementary material for: New records of a lost species and a geographic range expansion for sengis in the Horn of Africa
Source: PeerJ. 2020 Aug 18;8:e9652. doi: 10.7717/peerj.9652 (PMC7441985; doi:10.7717/peerj.9652)
Supplement: Supplemental Information 3 [file peerj-08-9652-s003.zip › Data_S3_DNA_Alignments_and_Phylogenetic_Analysis/2_Phylogenetic_Analysis/2_MrBayes/2_MrBayes_results/Heritage.et.al.2020_Sengis_Bayesian_Phylogenetic_Analaysis_TIME-SCALED.pdf]

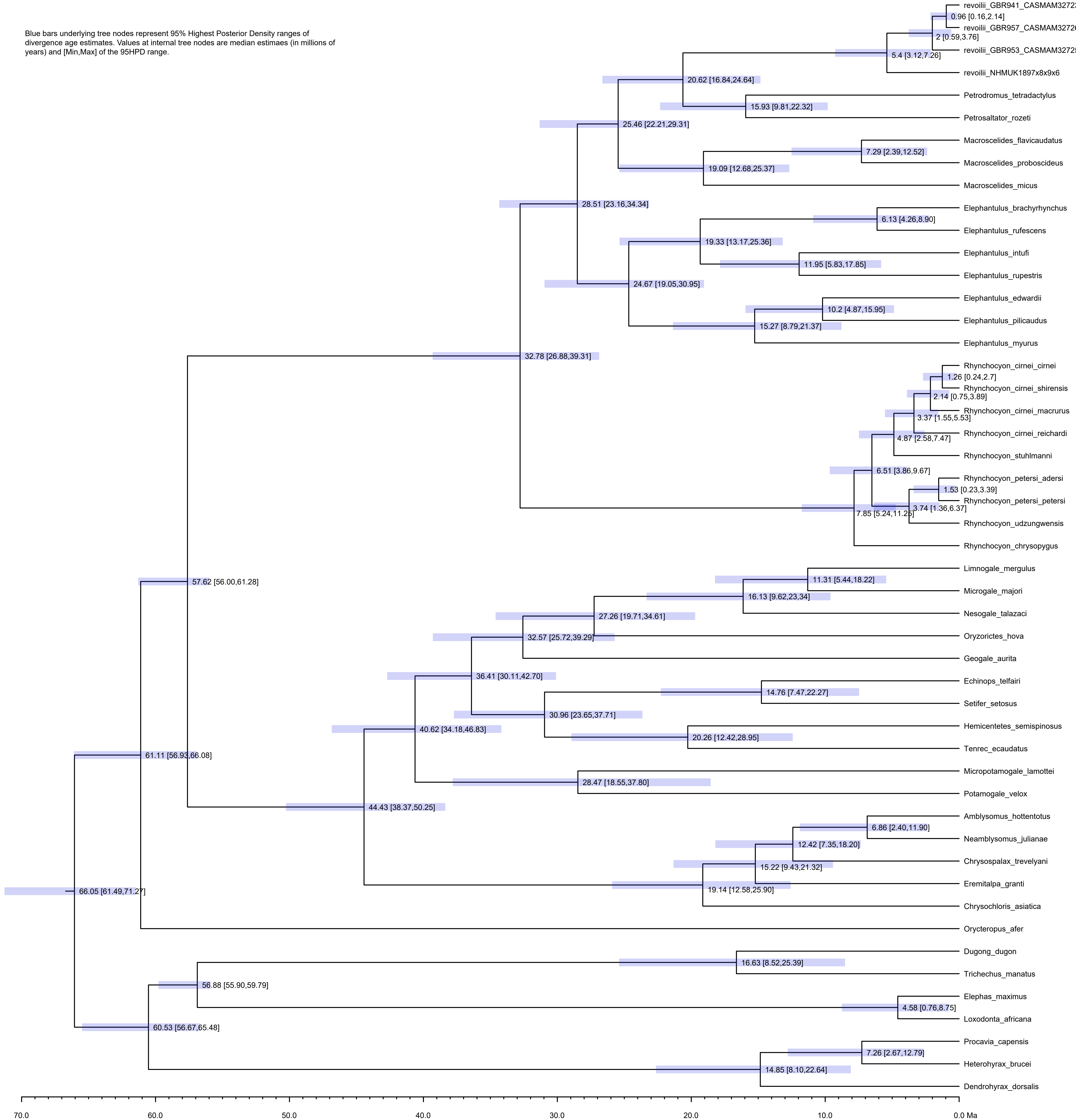

Blue bars underlying tree nodes represent 95% Highest Posterior Density ranges of divergence age estimates. Values at internal tree nodes are median estimaes (in millions of years) and [Min,Max] of the 95HPD range.
revoilii_GBR941_CASMAM3272
0.96 [0.16,2.14]
revoilii_GBR957_CASMAM3272
2 [0.59,3.76]
revoilii_GBR953_CASMAM3272
5.4 [3.12,7.26]
revoilii_NHMUK1897x8x9x6
20.62 [16.84,24.64]
Petrodromus_tetradactylus
15.93 [9.81,22.32]
Petrosaltator_rozeti
25.46 [22.21,29.31]
Macroscelides_flavicaudatus
7.29 [2.39,12.52]
Macroscelides_proboscideus
19.09 [12.68,25.37]
Macroscelides_micus
28.51 [23.16,34.34]
Elephantulus_brachyrhynchus
6.13 [4.26,8.90]
Elephantulus_rufescens
19.33 [13.17,25.36]
Elephantulus_intufi
11.95 [5.83,17.85]
Elephantulus_rupestris
24.67 [19.05,30.95]
Elephantulus_edwardii
10.2 [4.87,15.95]
Elephantulus_pilicaudus
15.27 [8.79,21.37]
Elephantulus_myurus
32.78 [26.88,39.31]
Rhynchocyon_cirnei_cirnei
1.26 [0.24,2.7]
Rhynchocyon_cirnei_shirensis
2.14 [0.75,3.89]
Rhynchocyon_cirnei_macrurus
3.37 [1.55,5.53]
Rhynchocyon_cirnei_reichardi
4.87 [2.58,7.47]
Rhynchocyon_stuhlmanni
6.51 [3.86,9.67]
Rhynchocyon_petersi_adersi
1.53 [0.23,3.39]
Rhynchocyon_petersi_petersi
3.74 [1.36,6.37]
7.85 [5.24,11.26]
Rhynchocyon_udzungwensis
Rhynchocyon_chrysopygus
57.62 [56.00,61.28]
Limnogale_mergulus
11.31 [5.44,18.22]
Microgale_majori
16.13 [9.62,23,34]
Nesogale_talazaci
27.26 [19.71,34.61]
Oryzorictes_hova
32.57 [25.72,39.29]
Geogale_aurita
36.41 [30.11,42.70]
Echinops_telfairi
14.76 [7.47,22.27]
Setifer_setosus
30.96 [23.65,37.71]
Hemicentetes_semispinosus
40.62 [34.18,46.83]
20.26 [12.42,28.95]
Tenrec_ecaudatus
61.11 [56.93,66.08]
Micropotamogale_lamottei
28.47 [18.55,37.80]
Potamogale_velox
44.43 [38.37,50.25]
Amblysomus_hottentotus
6.86 [2.40,11.90]
Neamblysomus_julianae
12.42 [7.35,18.20]
Chrysospalax_trevelyani
15.22 [9.43,21.32]
Eremitalpa_granti
66.05 [61.49,71.27]
19.14 [12.58,25.90]
Chrysochloris_asiatica
Orycteropus_afer
Dugong_dugon
16.63 [8.52,25.39]
Trichechus_manatus
56.88 [55.90,59.79]
Elephas_maximus
4.58 [0.76,8.75]
Loxodonta_africana
60.53 [56.67,65.48]
Procavia_capensis
7.26 [2.67,12.79]
Heterohyrax_brucei
14.85 [8.10,22.64]
Dendrohyrax_dorsalis
70.0
60.0
50.0
40.0
30.0
20.0
10.0
0.0 Ma
