# Supplementary figures and images for: New records of a lost species and a geographic range expansion for sengis in the Horn of Africa (part 1 of 2)
Source: PeerJ. 2020 Aug 18;8:e9652. doi: 10.7717/peerj.9652 (PMC7441985; doi:10.7717/peerj.9652)

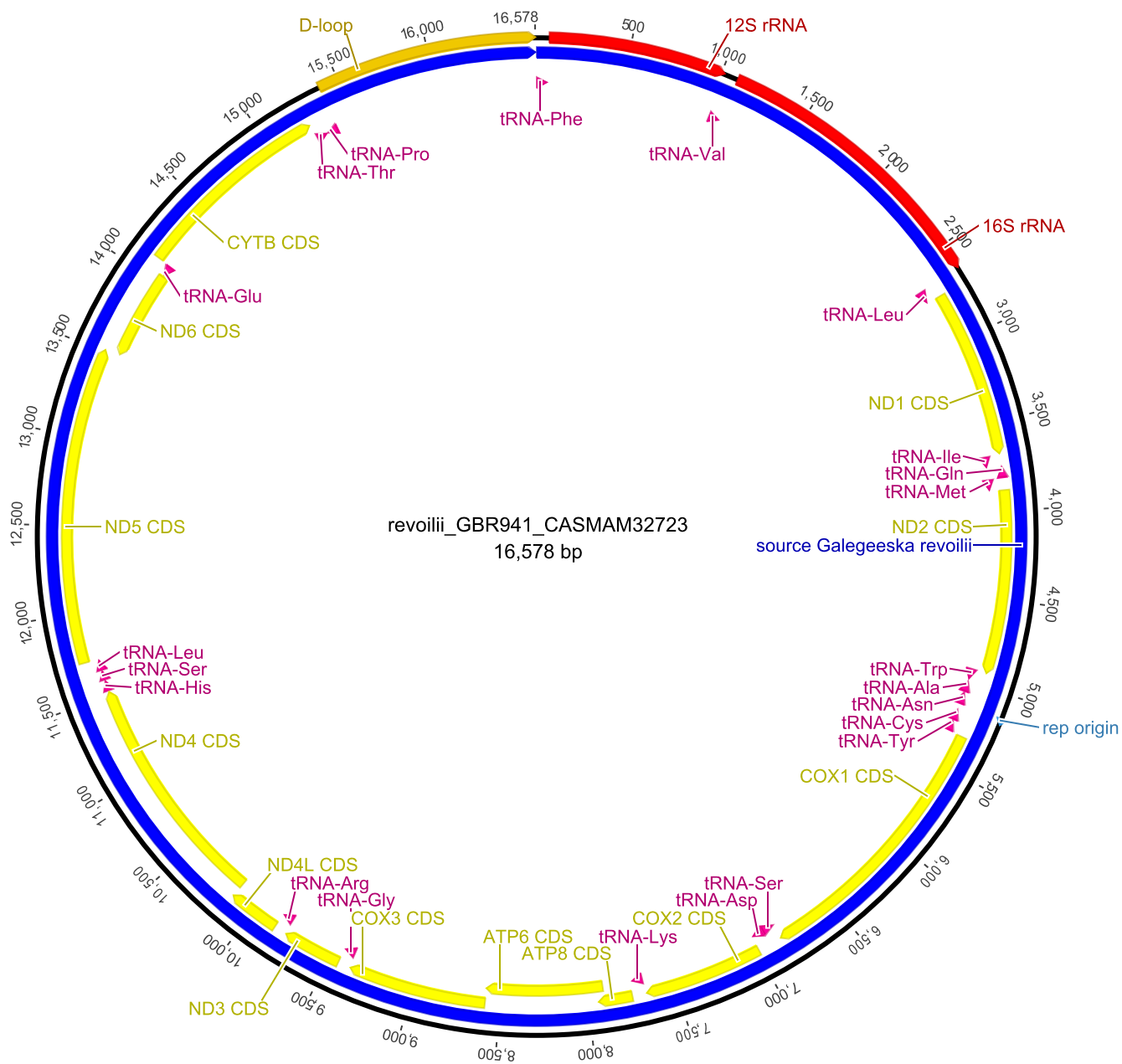

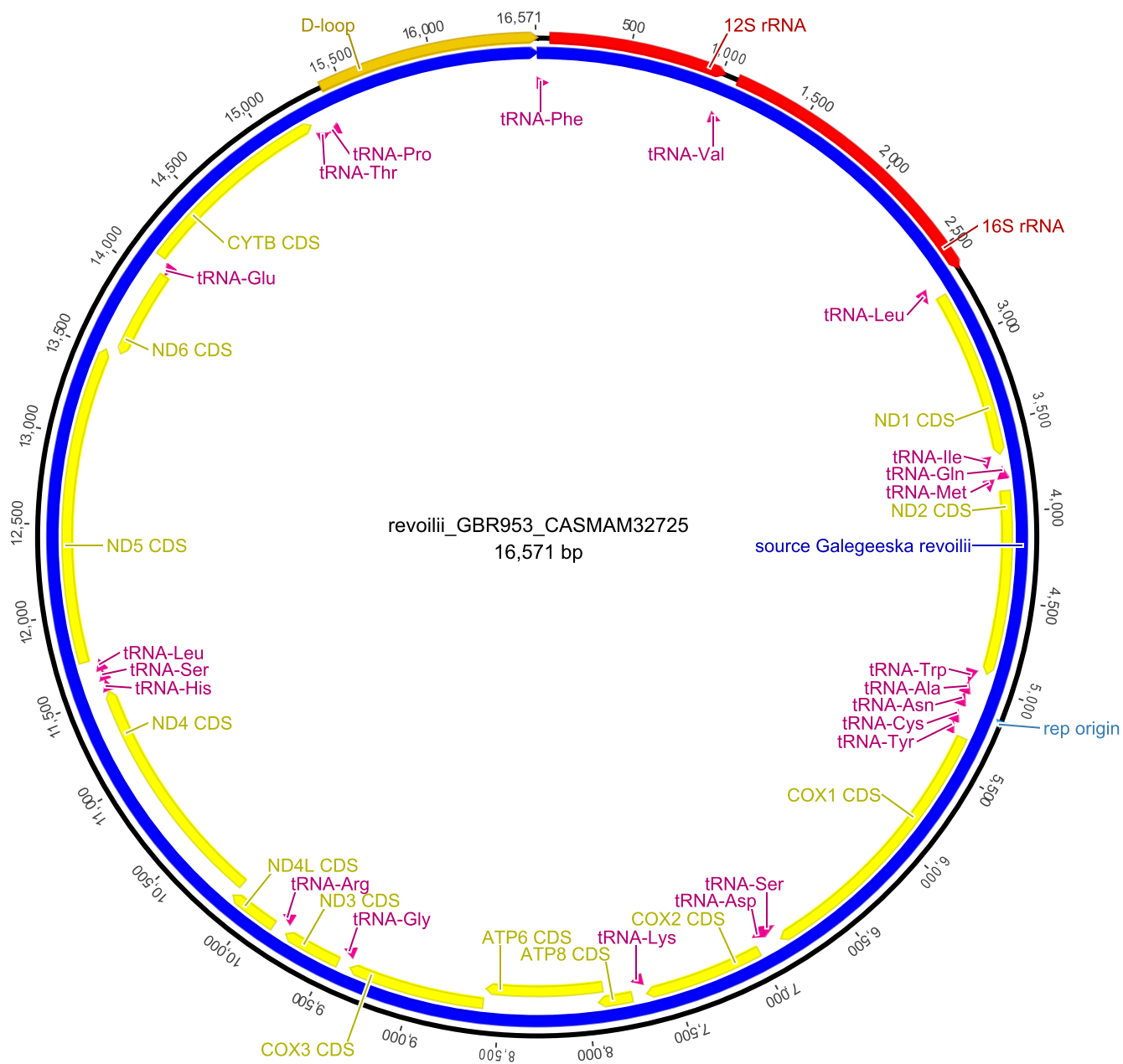

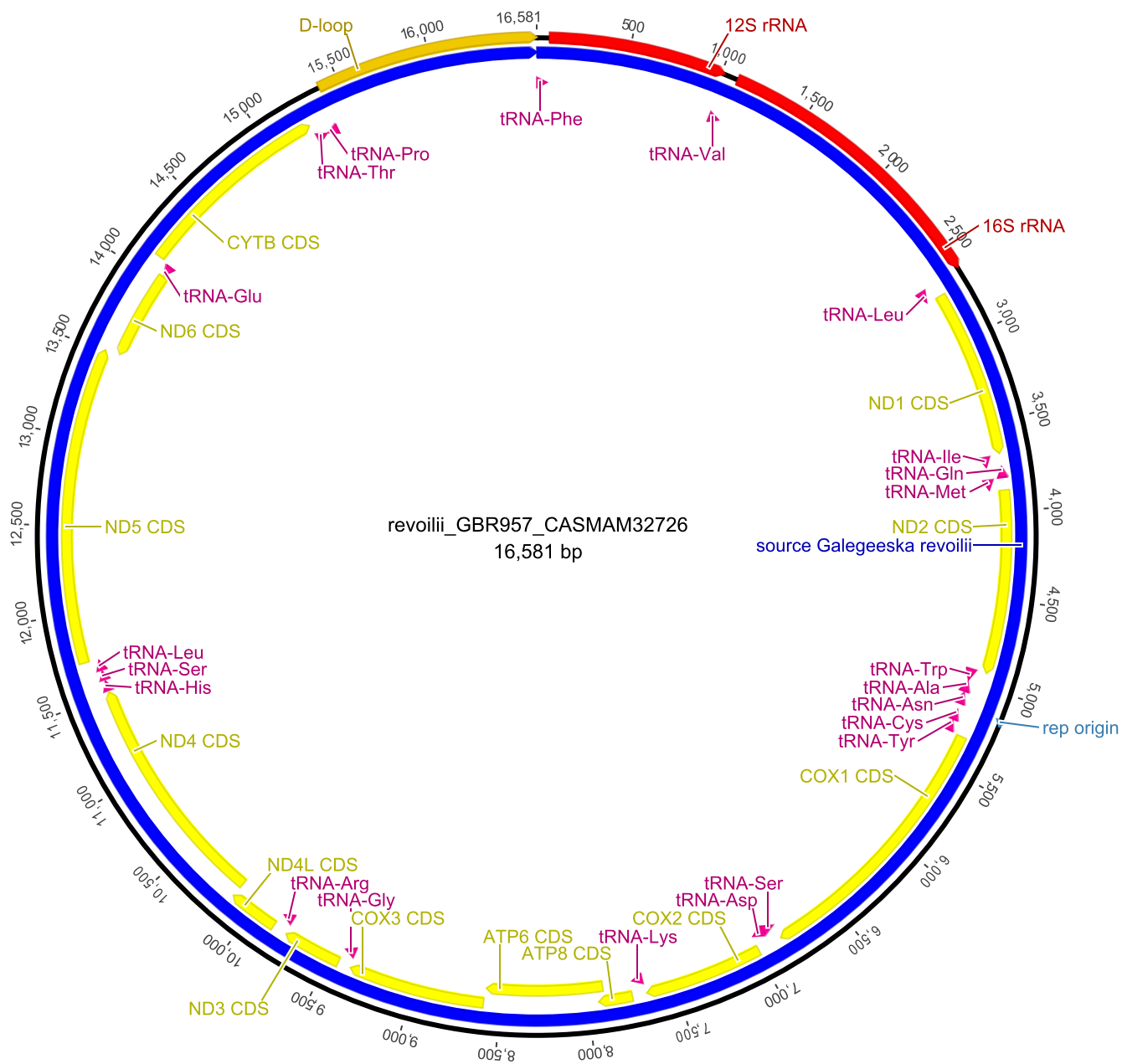

Supplement: Supplemental Information 3 [file peerj-08-9652-s003.zip › Data_S3_DNA_Alignments_and_Phylogenetic_Analysis/1_DNA_Alignments/complete_mitochondrial_genomes/Heritage.et.al.2020_Galegeeska_revoilii__3_Mitochondrial_Genomes_GRAPHICAL.pdf]

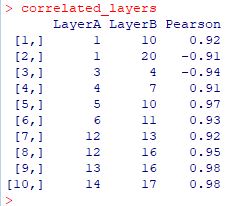

Supplement: Supplemental Information 6 [file peerj-08-9652-s006.zip › Data_S6_Ecological_Niche_Modeling/1_collinear_pairs/_correlated_layer_pairs.JPG]

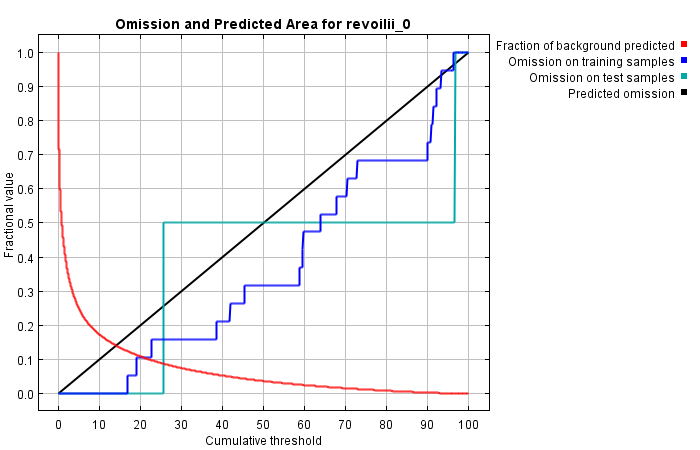

Supplement: Supplemental Information 6 [file peerj-08-9652-s006.zip › Data_S6_Ecological_Niche_Modeling/4_final_Maxent_analysis/output/plots/revoilii_0_omission.png]

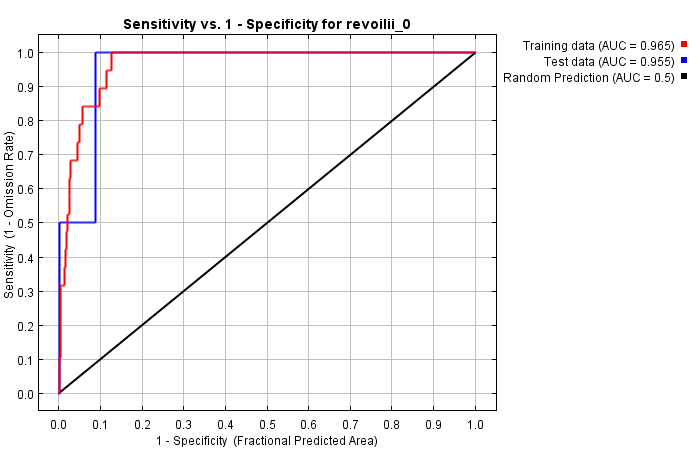

Supplement: Supplemental Information 6 [file peerj-08-9652-s006.zip › Data_S6_Ecological_Niche_Modeling/4_final_Maxent_analysis/output/plots/revoilii_0_roc.png]

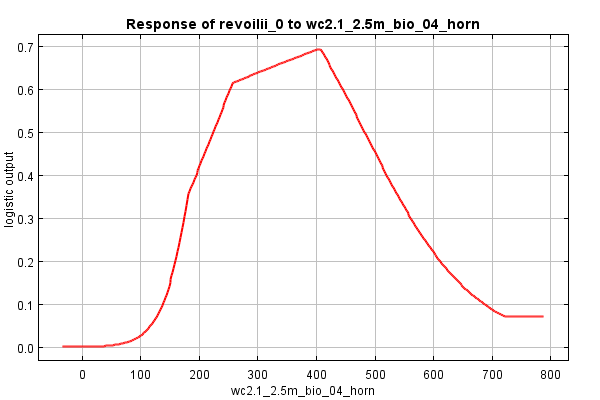

Supplement: Supplemental Information 6 [file peerj-08-9652-s006.zip › Data_S6_Ecological_Niche_Modeling/4_final_Maxent_analysis/output/plots/revoilii_0_wc2.1_2.5m_bio_04_horn.png]

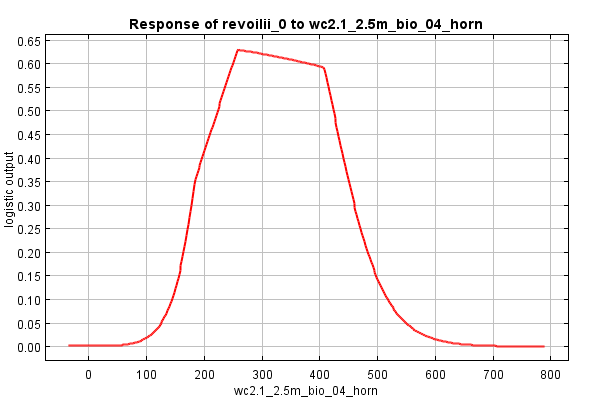

Supplement: Supplemental Information 6 [file peerj-08-9652-s006.zip › Data_S6_Ecological_Niche_Modeling/4_final_Maxent_analysis/output/plots/revoilii_0_wc2.1_2.5m_bio_04_horn_only.png]

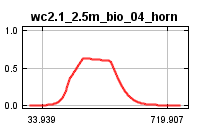

Supplement: Supplemental Information 6 [file peerj-08-9652-s006.zip › Data_S6_Ecological_Niche_Modeling/4_final_Maxent_analysis/output/plots/revoilii_0_wc2.1_2.5m_bio_04_horn_only_thumb.png]

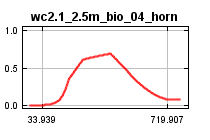

Supplement: Supplemental Information 6 [file peerj-08-9652-s006.zip › Data_S6_Ecological_Niche_Modeling/4_final_Maxent_analysis/output/plots/revoilii_0_wc2.1_2.5m_bio_04_horn_thumb.png]

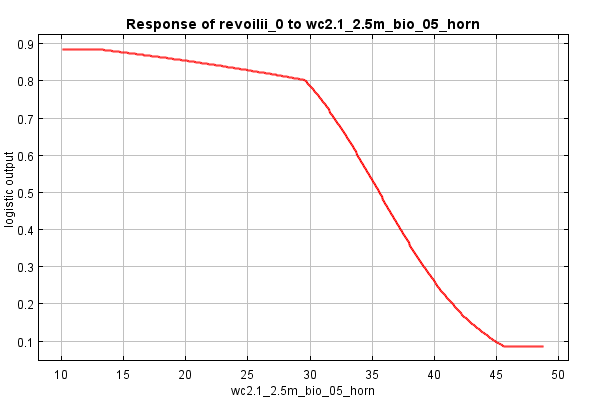

Supplement: Supplemental Information 6 [file peerj-08-9652-s006.zip › Data_S6_Ecological_Niche_Modeling/4_final_Maxent_analysis/output/plots/revoilii_0_wc2.1_2.5m_bio_05_horn.png]

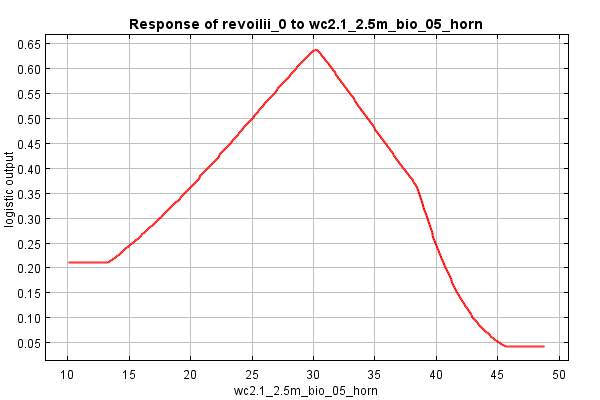

Supplement: Supplemental Information 6 [file peerj-08-9652-s006.zip › Data_S6_Ecological_Niche_Modeling/4_final_Maxent_analysis/output/plots/revoilii_0_wc2.1_2.5m_bio_05_horn_only.png]

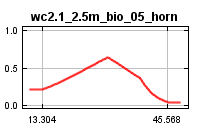

Supplement: Supplemental Information 6 [file peerj-08-9652-s006.zip › Data_S6_Ecological_Niche_Modeling/4_final_Maxent_analysis/output/plots/revoilii_0_wc2.1_2.5m_bio_05_horn_only_thumb.png]

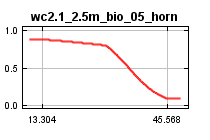

Supplement: Supplemental Information 6 [file peerj-08-9652-s006.zip › Data_S6_Ecological_Niche_Modeling/4_final_Maxent_analysis/output/plots/revoilii_0_wc2.1_2.5m_bio_05_horn_thumb.png]

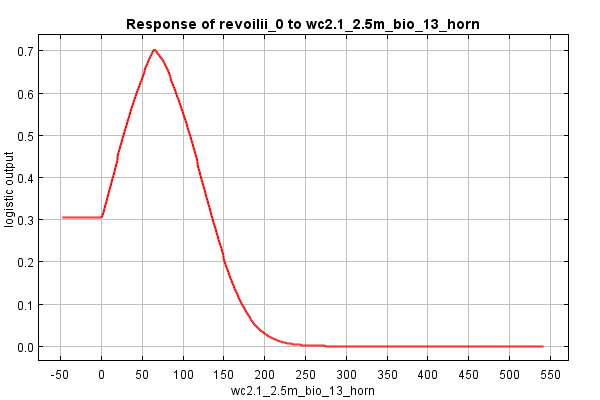

Supplement: Supplemental Information 6 [file peerj-08-9652-s006.zip › Data_S6_Ecological_Niche_Modeling/4_final_Maxent_analysis/output/plots/revoilii_0_wc2.1_2.5m_bio_13_horn.png]

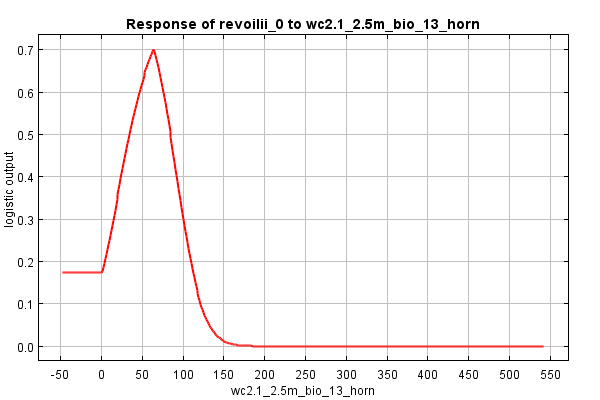

Supplement: Supplemental Information 6 [file peerj-08-9652-s006.zip › Data_S6_Ecological_Niche_Modeling/4_final_Maxent_analysis/output/plots/revoilii_0_wc2.1_2.5m_bio_13_horn_only.png]

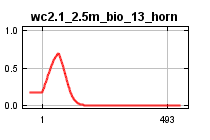

Supplement: Supplemental Information 6 [file peerj-08-9652-s006.zip › Data_S6_Ecological_Niche_Modeling/4_final_Maxent_analysis/output/plots/revoilii_0_wc2.1_2.5m_bio_13_horn_only_thumb.png]

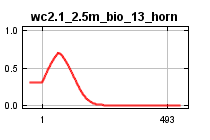

Supplement: Supplemental Information 6 [file peerj-08-9652-s006.zip › Data_S6_Ecological_Niche_Modeling/4_final_Maxent_analysis/output/plots/revoilii_0_wc2.1_2.5m_bio_13_horn_thumb.png]

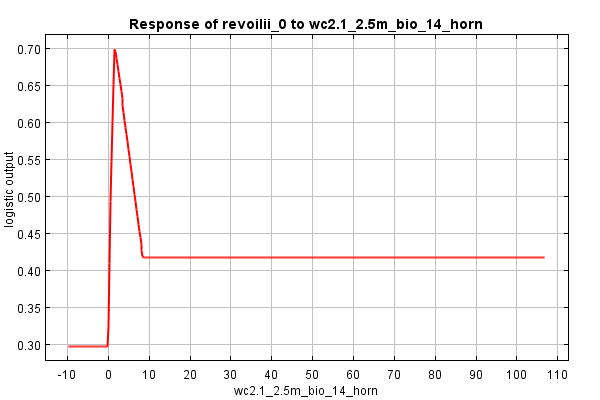

Supplement: Supplemental Information 6 [file peerj-08-9652-s006.zip › Data_S6_Ecological_Niche_Modeling/4_final_Maxent_analysis/output/plots/revoilii_0_wc2.1_2.5m_bio_14_horn.png]

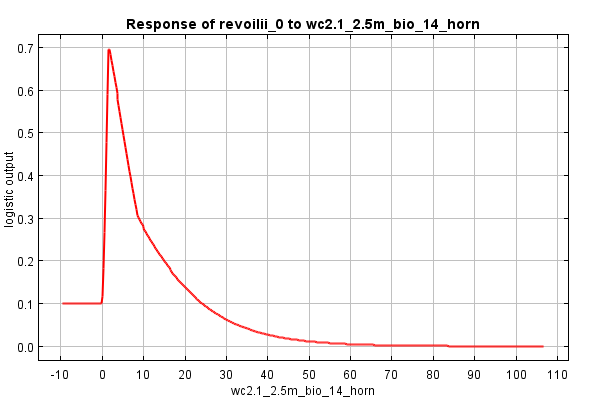

Supplement: Supplemental Information 6 [file peerj-08-9652-s006.zip › Data_S6_Ecological_Niche_Modeling/4_final_Maxent_analysis/output/plots/revoilii_0_wc2.1_2.5m_bio_14_horn_only.png]

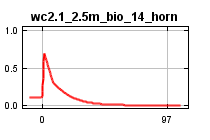

Supplement: Supplemental Information 6 [file peerj-08-9652-s006.zip › Data_S6_Ecological_Niche_Modeling/4_final_Maxent_analysis/output/plots/revoilii_0_wc2.1_2.5m_bio_14_horn_only_thumb.png]

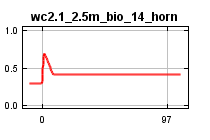

Supplement: Supplemental Information 6 [file peerj-08-9652-s006.zip › Data_S6_Ecological_Niche_Modeling/4_final_Maxent_analysis/output/plots/revoilii_0_wc2.1_2.5m_bio_14_horn_thumb.png]

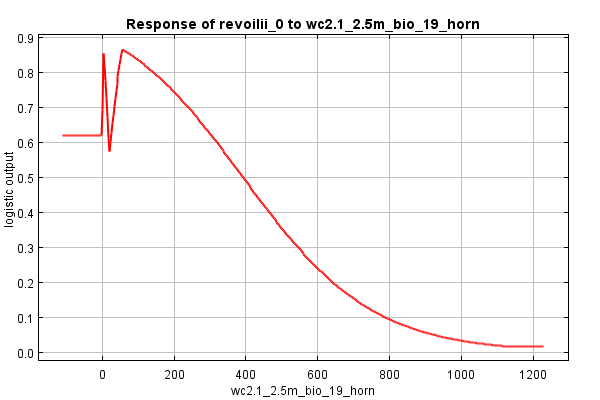

Supplement: Supplemental Information 6 [file peerj-08-9652-s006.zip › Data_S6_Ecological_Niche_Modeling/4_final_Maxent_analysis/output/plots/revoilii_0_wc2.1_2.5m_bio_19_horn.png]

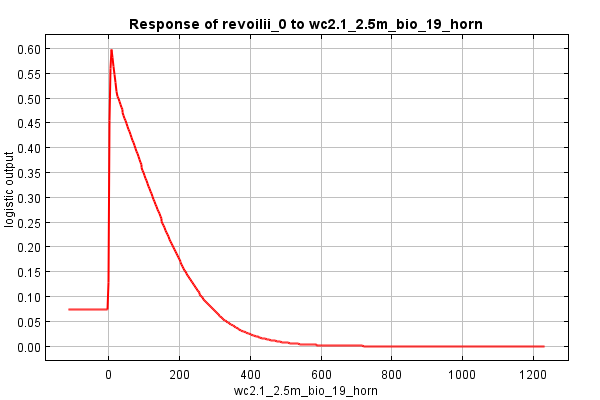

Supplement: Supplemental Information 6 [file peerj-08-9652-s006.zip › Data_S6_Ecological_Niche_Modeling/4_final_Maxent_analysis/output/plots/revoilii_0_wc2.1_2.5m_bio_19_horn_only.png]

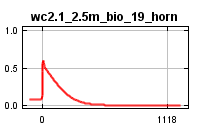

Supplement: Supplemental Information 6 [file peerj-08-9652-s006.zip › Data_S6_Ecological_Niche_Modeling/4_final_Maxent_analysis/output/plots/revoilii_0_wc2.1_2.5m_bio_19_horn_only_thumb.png]

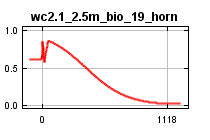

Supplement: Supplemental Information 6 [file peerj-08-9652-s006.zip › Data_S6_Ecological_Niche_Modeling/4_final_Maxent_analysis/output/plots/revoilii_0_wc2.1_2.5m_bio_19_horn_thumb.png]

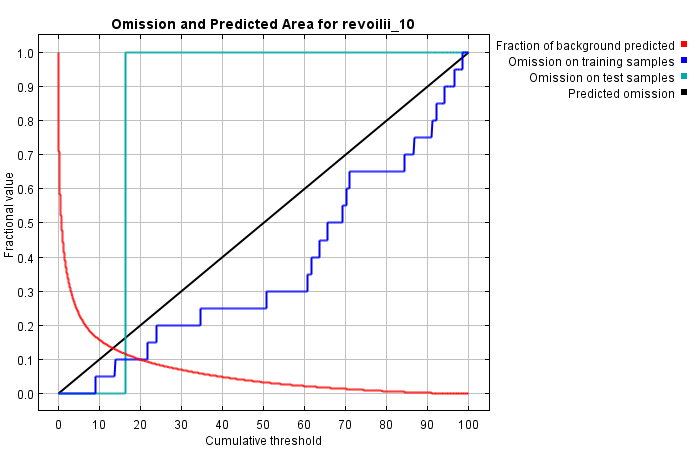

Supplement: Supplemental Information 6 [file peerj-08-9652-s006.zip › Data_S6_Ecological_Niche_Modeling/4_final_Maxent_analysis/output/plots/revoilii_10_omission.png]

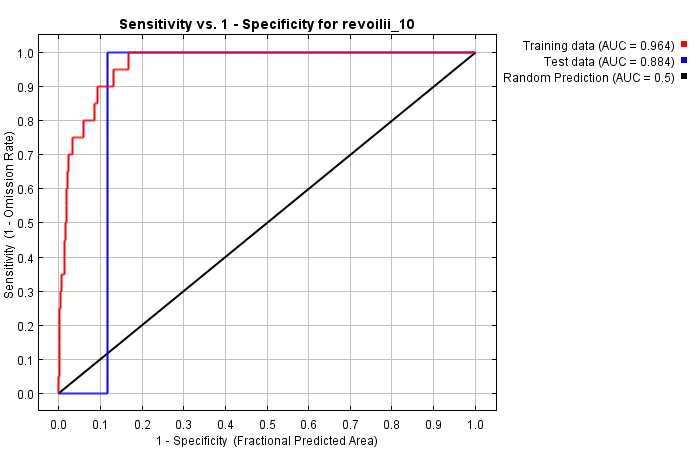

Supplement: Supplemental Information 6 [file peerj-08-9652-s006.zip › Data_S6_Ecological_Niche_Modeling/4_final_Maxent_analysis/output/plots/revoilii_10_roc.png]

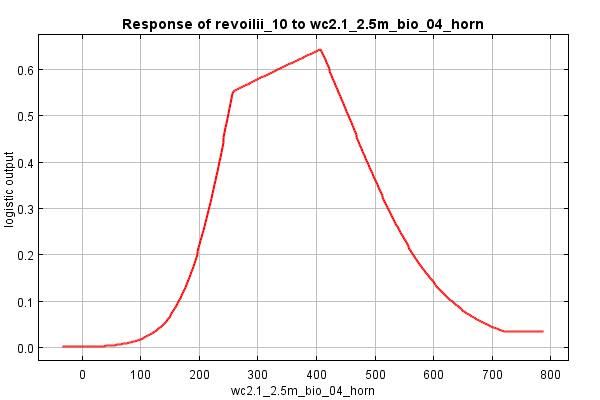

Supplement: Supplemental Information 6 [file peerj-08-9652-s006.zip › Data_S6_Ecological_Niche_Modeling/4_final_Maxent_analysis/output/plots/revoilii_10_wc2.1_2.5m_bio_04_horn.png]

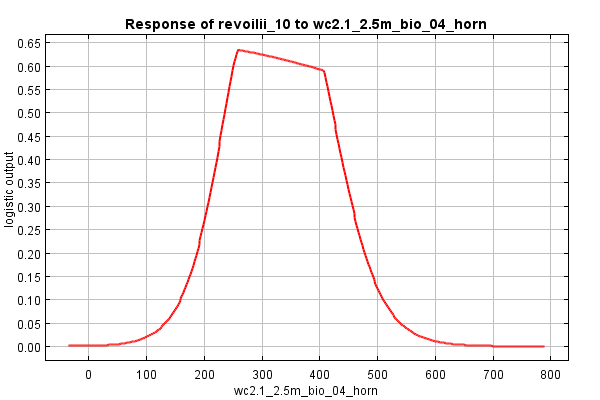

Supplement: Supplemental Information 6 [file peerj-08-9652-s006.zip › Data_S6_Ecological_Niche_Modeling/4_final_Maxent_analysis/output/plots/revoilii_10_wc2.1_2.5m_bio_04_horn_only.png]

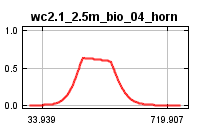

Supplement: Supplemental Information 6 [file peerj-08-9652-s006.zip › Data_S6_Ecological_Niche_Modeling/4_final_Maxent_analysis/output/plots/revoilii_10_wc2.1_2.5m_bio_04_horn_only_thumb.png]

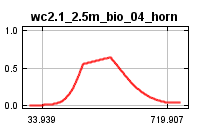

Supplement: Supplemental Information 6 [file peerj-08-9652-s006.zip › Data_S6_Ecological_Niche_Modeling/4_final_Maxent_analysis/output/plots/revoilii_10_wc2.1_2.5m_bio_04_horn_thumb.png]

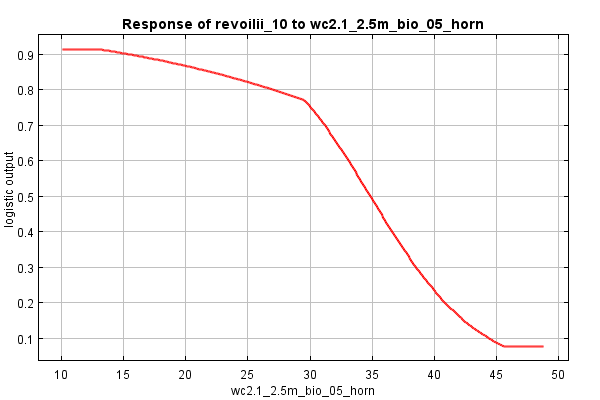

Supplement: Supplemental Information 6 [file peerj-08-9652-s006.zip › Data_S6_Ecological_Niche_Modeling/4_final_Maxent_analysis/output/plots/revoilii_10_wc2.1_2.5m_bio_05_horn.png]

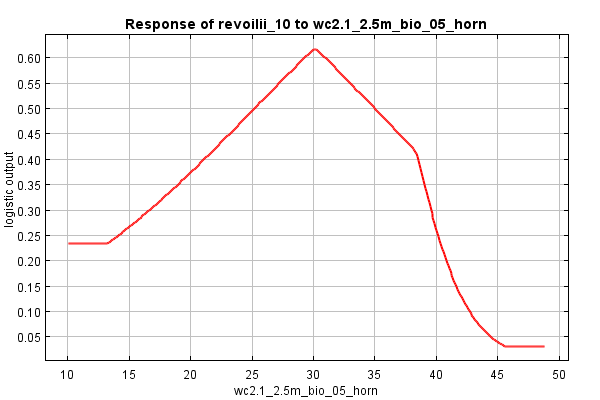

Supplement: Supplemental Information 6 [file peerj-08-9652-s006.zip › Data_S6_Ecological_Niche_Modeling/4_final_Maxent_analysis/output/plots/revoilii_10_wc2.1_2.5m_bio_05_horn_only.png]

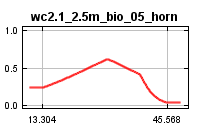

Supplement: Supplemental Information 6 [file peerj-08-9652-s006.zip › Data_S6_Ecological_Niche_Modeling/4_final_Maxent_analysis/output/plots/revoilii_10_wc2.1_2.5m_bio_05_horn_only_thumb.png]

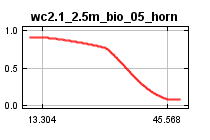

Supplement: Supplemental Information 6 [file peerj-08-9652-s006.zip › Data_S6_Ecological_Niche_Modeling/4_final_Maxent_analysis/output/plots/revoilii_10_wc2.1_2.5m_bio_05_horn_thumb.png]

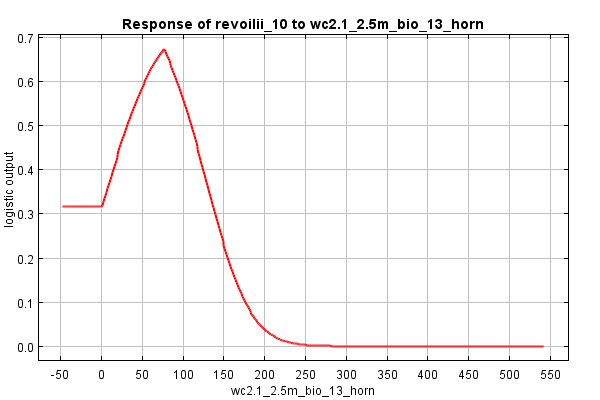

Supplement: Supplemental Information 6 [file peerj-08-9652-s006.zip › Data_S6_Ecological_Niche_Modeling/4_final_Maxent_analysis/output/plots/revoilii_10_wc2.1_2.5m_bio_13_horn.png]

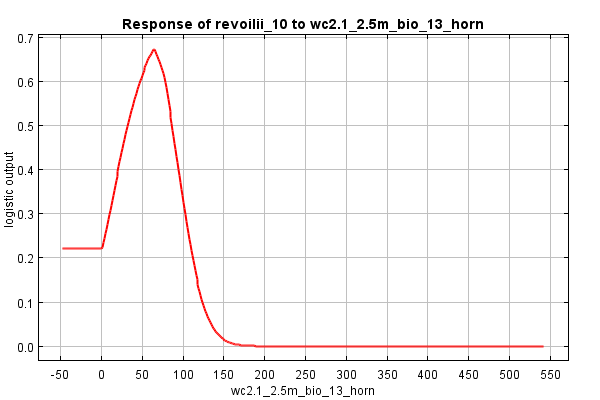

Supplement: Supplemental Information 6 [file peerj-08-9652-s006.zip › Data_S6_Ecological_Niche_Modeling/4_final_Maxent_analysis/output/plots/revoilii_10_wc2.1_2.5m_bio_13_horn_only.png]

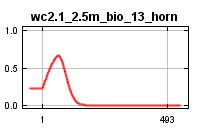

Supplement: Supplemental Information 6 [file peerj-08-9652-s006.zip › Data_S6_Ecological_Niche_Modeling/4_final_Maxent_analysis/output/plots/revoilii_10_wc2.1_2.5m_bio_13_horn_only_thumb.png]

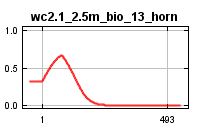

Supplement: Supplemental Information 6 [file peerj-08-9652-s006.zip › Data_S6_Ecological_Niche_Modeling/4_final_Maxent_analysis/output/plots/revoilii_10_wc2.1_2.5m_bio_13_horn_thumb.png]

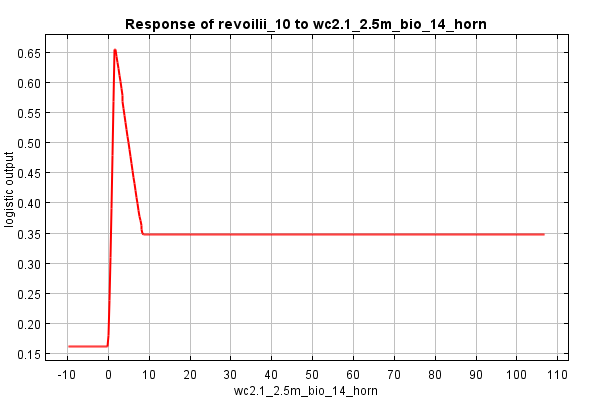

Supplement: Supplemental Information 6 [file peerj-08-9652-s006.zip › Data_S6_Ecological_Niche_Modeling/4_final_Maxent_analysis/output/plots/revoilii_10_wc2.1_2.5m_bio_14_horn.png]

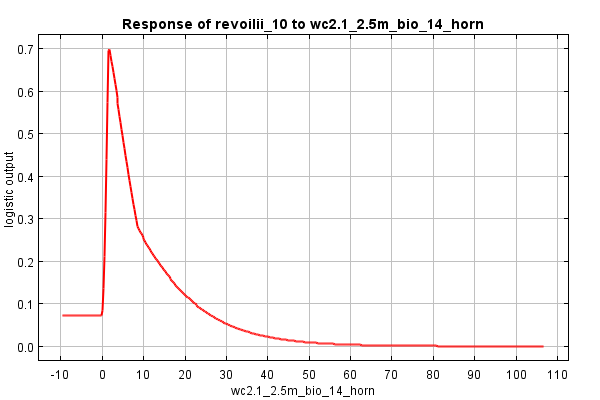

Supplement: Supplemental Information 6 [file peerj-08-9652-s006.zip › Data_S6_Ecological_Niche_Modeling/4_final_Maxent_analysis/output/plots/revoilii_10_wc2.1_2.5m_bio_14_horn_only.png]

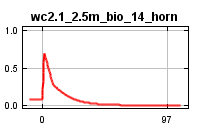

Supplement: Supplemental Information 6 [file peerj-08-9652-s006.zip › Data_S6_Ecological_Niche_Modeling/4_final_Maxent_analysis/output/plots/revoilii_10_wc2.1_2.5m_bio_14_horn_only_thumb.png]

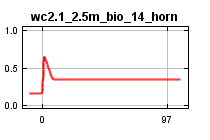

Supplement: Supplemental Information 6 [file peerj-08-9652-s006.zip › Data_S6_Ecological_Niche_Modeling/4_final_Maxent_analysis/output/plots/revoilii_10_wc2.1_2.5m_bio_14_horn_thumb.png]

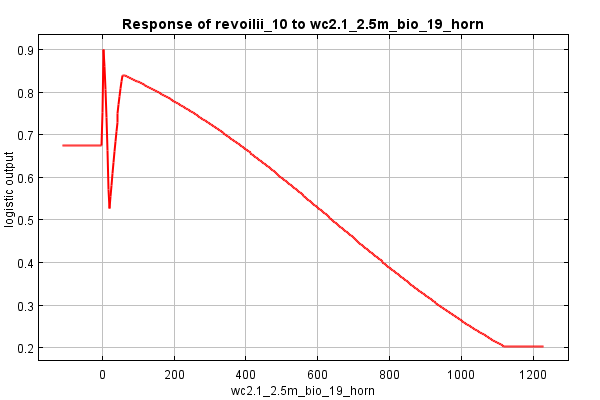

Supplement: Supplemental Information 6 [file peerj-08-9652-s006.zip › Data_S6_Ecological_Niche_Modeling/4_final_Maxent_analysis/output/plots/revoilii_10_wc2.1_2.5m_bio_19_horn.png]

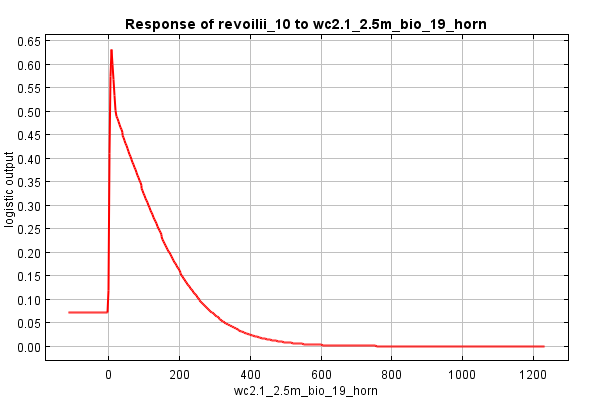

Supplement: Supplemental Information 6 [file peerj-08-9652-s006.zip › Data_S6_Ecological_Niche_Modeling/4_final_Maxent_analysis/output/plots/revoilii_10_wc2.1_2.5m_bio_19_horn_only.png]

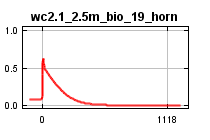

Supplement: Supplemental Information 6 [file peerj-08-9652-s006.zip › Data_S6_Ecological_Niche_Modeling/4_final_Maxent_analysis/output/plots/revoilii_10_wc2.1_2.5m_bio_19_horn_only_thumb.png]

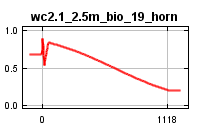

Supplement: Supplemental Information 6 [file peerj-08-9652-s006.zip › Data_S6_Ecological_Niche_Modeling/4_final_Maxent_analysis/output/plots/revoilii_10_wc2.1_2.5m_bio_19_horn_thumb.png]

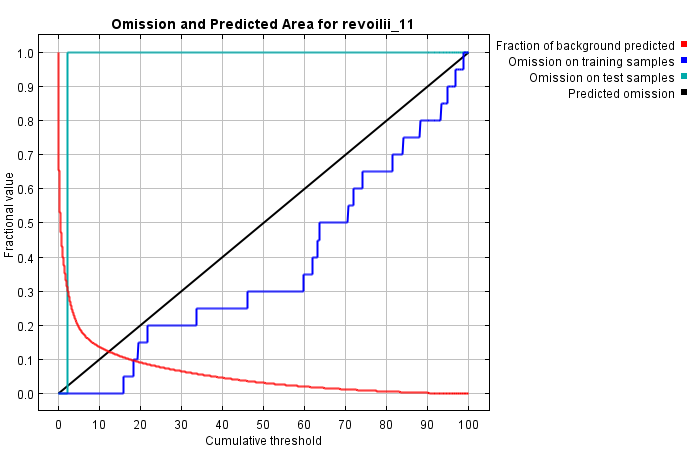

Supplement: Supplemental Information 6 [file peerj-08-9652-s006.zip › Data_S6_Ecological_Niche_Modeling/4_final_Maxent_analysis/output/plots/revoilii_11_omission.png]

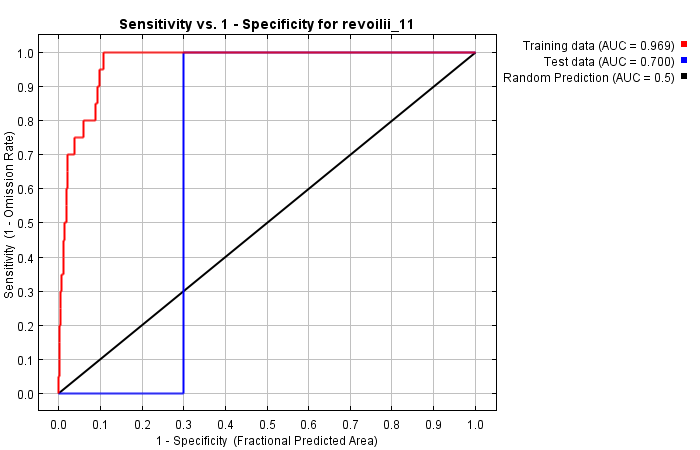

Supplement: Supplemental Information 6 [file peerj-08-9652-s006.zip › Data_S6_Ecological_Niche_Modeling/4_final_Maxent_analysis/output/plots/revoilii_11_roc.png]

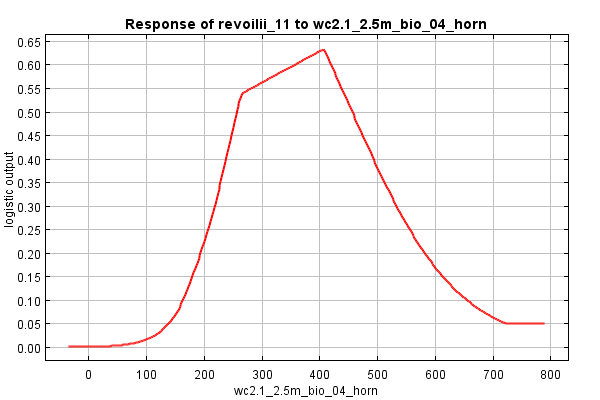

Supplement: Supplemental Information 6 [file peerj-08-9652-s006.zip › Data_S6_Ecological_Niche_Modeling/4_final_Maxent_analysis/output/plots/revoilii_11_wc2.1_2.5m_bio_04_horn.png]

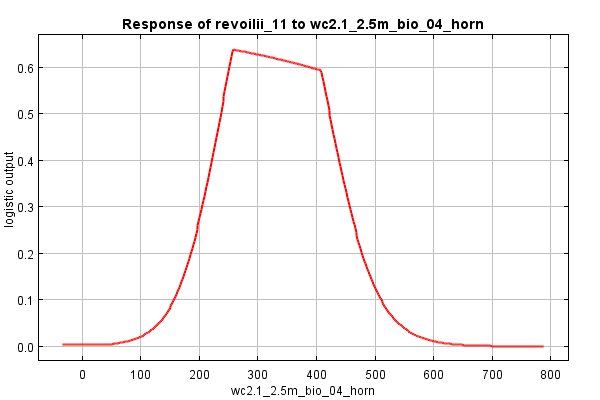

Supplement: Supplemental Information 6 [file peerj-08-9652-s006.zip › Data_S6_Ecological_Niche_Modeling/4_final_Maxent_analysis/output/plots/revoilii_11_wc2.1_2.5m_bio_04_horn_only.png]

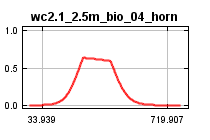

Supplement: Supplemental Information 6 [file peerj-08-9652-s006.zip › Data_S6_Ecological_Niche_Modeling/4_final_Maxent_analysis/output/plots/revoilii_11_wc2.1_2.5m_bio_04_horn_only_thumb.png]

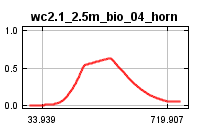

Supplement: Supplemental Information 6 [file peerj-08-9652-s006.zip › Data_S6_Ecological_Niche_Modeling/4_final_Maxent_analysis/output/plots/revoilii_11_wc2.1_2.5m_bio_04_horn_thumb.png]

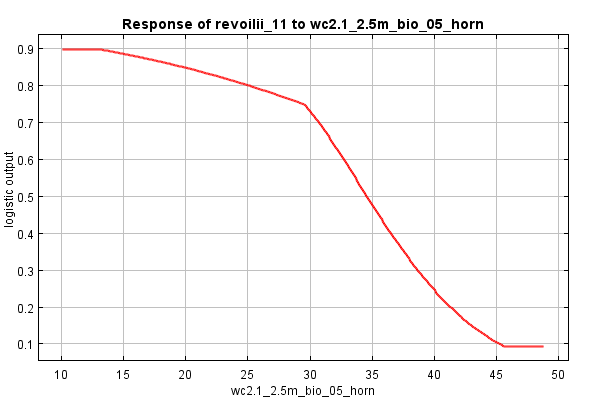

Supplement: Supplemental Information 6 [file peerj-08-9652-s006.zip › Data_S6_Ecological_Niche_Modeling/4_final_Maxent_analysis/output/plots/revoilii_11_wc2.1_2.5m_bio_05_horn.png]

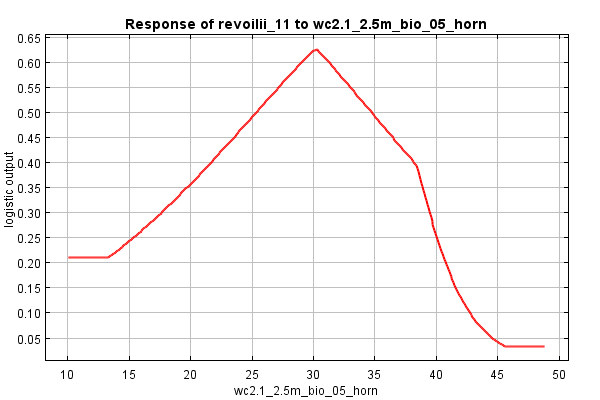

Supplement: Supplemental Information 6 [file peerj-08-9652-s006.zip › Data_S6_Ecological_Niche_Modeling/4_final_Maxent_analysis/output/plots/revoilii_11_wc2.1_2.5m_bio_05_horn_only.png]

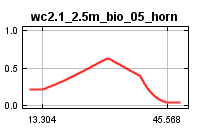

Supplement: Supplemental Information 6 [file peerj-08-9652-s006.zip › Data_S6_Ecological_Niche_Modeling/4_final_Maxent_analysis/output/plots/revoilii_11_wc2.1_2.5m_bio_05_horn_only_thumb.png]

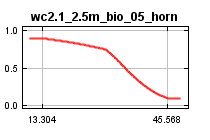

Supplement: Supplemental Information 6 [file peerj-08-9652-s006.zip › Data_S6_Ecological_Niche_Modeling/4_final_Maxent_analysis/output/plots/revoilii_11_wc2.1_2.5m_bio_05_horn_thumb.png]

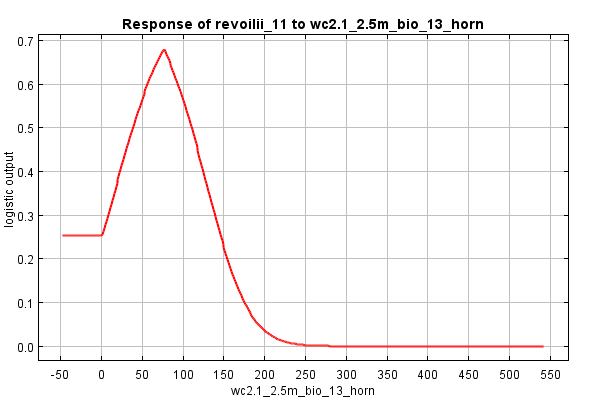

Supplement: Supplemental Information 6 [file peerj-08-9652-s006.zip › Data_S6_Ecological_Niche_Modeling/4_final_Maxent_analysis/output/plots/revoilii_11_wc2.1_2.5m_bio_13_horn.png]

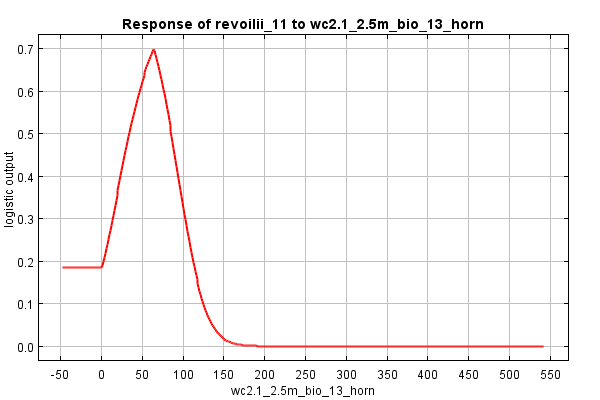

Supplement: Supplemental Information 6 [file peerj-08-9652-s006.zip › Data_S6_Ecological_Niche_Modeling/4_final_Maxent_analysis/output/plots/revoilii_11_wc2.1_2.5m_bio_13_horn_only.png]

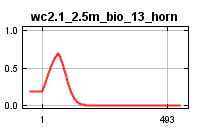

Supplement: Supplemental Information 6 [file peerj-08-9652-s006.zip › Data_S6_Ecological_Niche_Modeling/4_final_Maxent_analysis/output/plots/revoilii_11_wc2.1_2.5m_bio_13_horn_only_thumb.png]

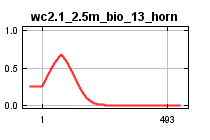

Supplement: Supplemental Information 6 [file peerj-08-9652-s006.zip › Data_S6_Ecological_Niche_Modeling/4_final_Maxent_analysis/output/plots/revoilii_11_wc2.1_2.5m_bio_13_horn_thumb.png]

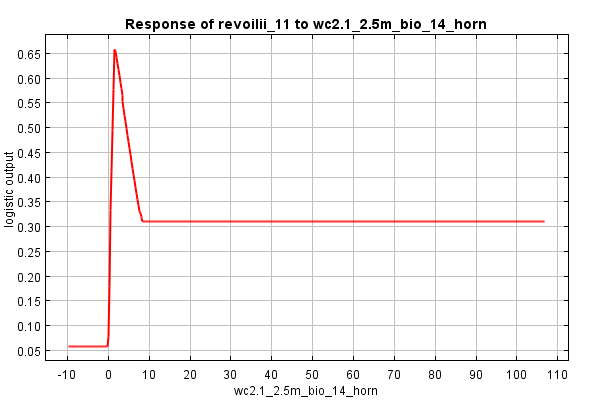

Supplement: Supplemental Information 6 [file peerj-08-9652-s006.zip › Data_S6_Ecological_Niche_Modeling/4_final_Maxent_analysis/output/plots/revoilii_11_wc2.1_2.5m_bio_14_horn.png]

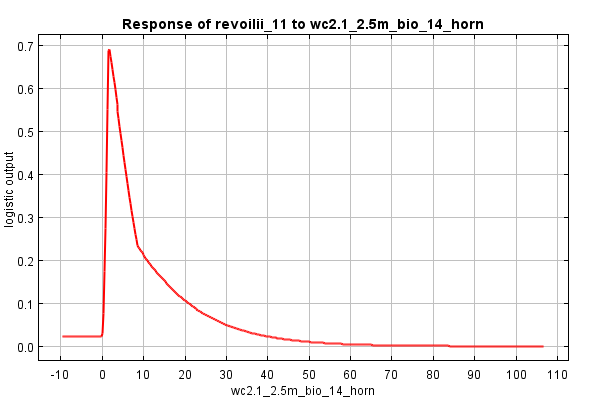

Supplement: Supplemental Information 6 [file peerj-08-9652-s006.zip › Data_S6_Ecological_Niche_Modeling/4_final_Maxent_analysis/output/plots/revoilii_11_wc2.1_2.5m_bio_14_horn_only.png]

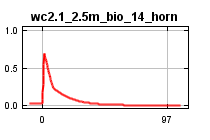

Supplement: Supplemental Information 6 [file peerj-08-9652-s006.zip › Data_S6_Ecological_Niche_Modeling/4_final_Maxent_analysis/output/plots/revoilii_11_wc2.1_2.5m_bio_14_horn_only_thumb.png]

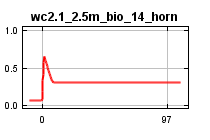

Supplement: Supplemental Information 6 [file peerj-08-9652-s006.zip › Data_S6_Ecological_Niche_Modeling/4_final_Maxent_analysis/output/plots/revoilii_11_wc2.1_2.5m_bio_14_horn_thumb.png]

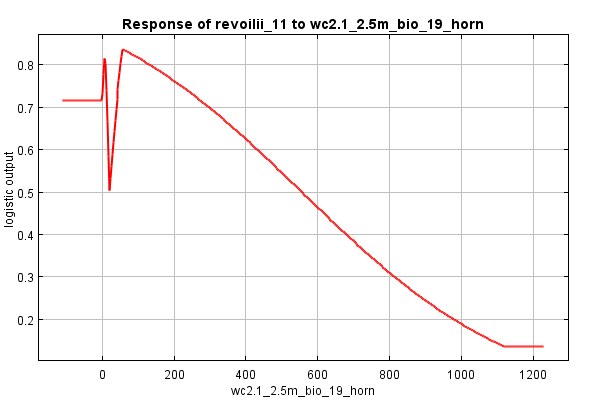

Supplement: Supplemental Information 6 [file peerj-08-9652-s006.zip › Data_S6_Ecological_Niche_Modeling/4_final_Maxent_analysis/output/plots/revoilii_11_wc2.1_2.5m_bio_19_horn.png]

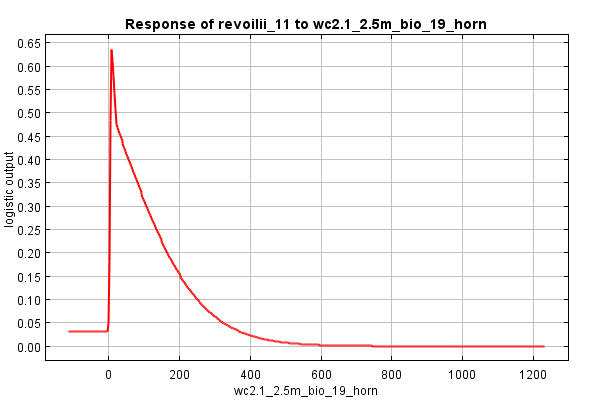

Supplement: Supplemental Information 6 [file peerj-08-9652-s006.zip › Data_S6_Ecological_Niche_Modeling/4_final_Maxent_analysis/output/plots/revoilii_11_wc2.1_2.5m_bio_19_horn_only.png]

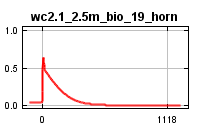

Supplement: Supplemental Information 6 [file peerj-08-9652-s006.zip › Data_S6_Ecological_Niche_Modeling/4_final_Maxent_analysis/output/plots/revoilii_11_wc2.1_2.5m_bio_19_horn_only_thumb.png]

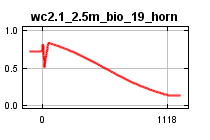

Supplement: Supplemental Information 6 [file peerj-08-9652-s006.zip › Data_S6_Ecological_Niche_Modeling/4_final_Maxent_analysis/output/plots/revoilii_11_wc2.1_2.5m_bio_19_horn_thumb.png]

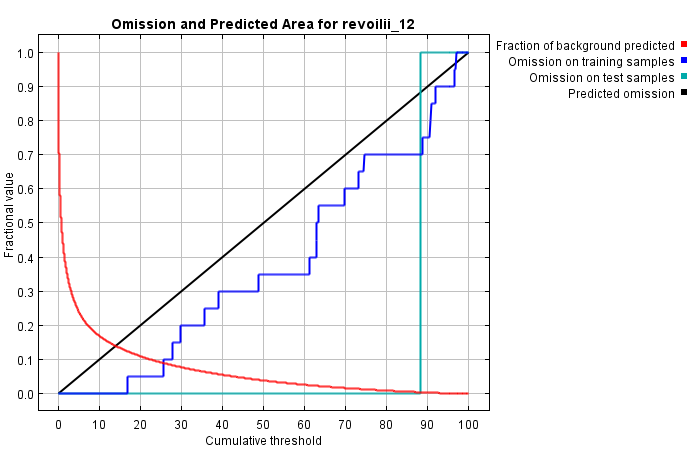

Supplement: Supplemental Information 6 [file peerj-08-9652-s006.zip › Data_S6_Ecological_Niche_Modeling/4_final_Maxent_analysis/output/plots/revoilii_12_omission.png]

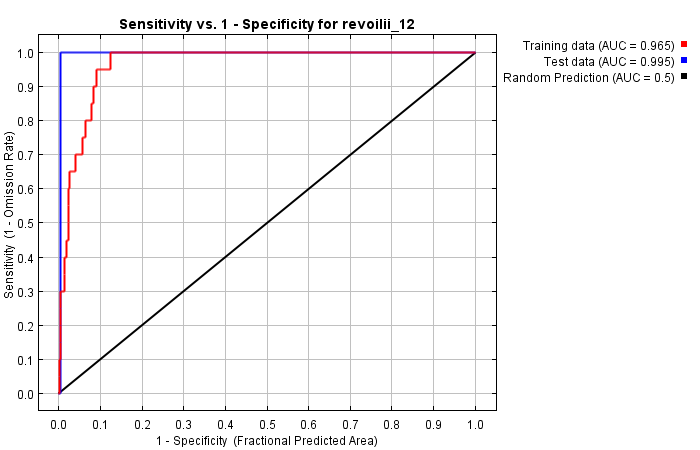

Supplement: Supplemental Information 6 [file peerj-08-9652-s006.zip › Data_S6_Ecological_Niche_Modeling/4_final_Maxent_analysis/output/plots/revoilii_12_roc.png]

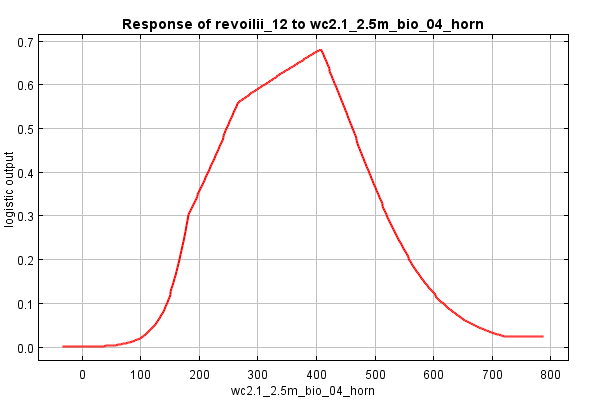

Supplement: Supplemental Information 6 [file peerj-08-9652-s006.zip › Data_S6_Ecological_Niche_Modeling/4_final_Maxent_analysis/output/plots/revoilii_12_wc2.1_2.5m_bio_04_horn.png]

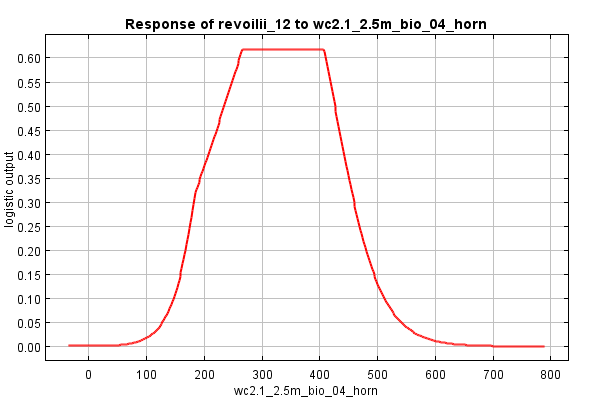

Supplement: Supplemental Information 6 [file peerj-08-9652-s006.zip › Data_S6_Ecological_Niche_Modeling/4_final_Maxent_analysis/output/plots/revoilii_12_wc2.1_2.5m_bio_04_horn_only.png]

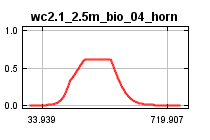

Supplement: Supplemental Information 6 [file peerj-08-9652-s006.zip › Data_S6_Ecological_Niche_Modeling/4_final_Maxent_analysis/output/plots/revoilii_12_wc2.1_2.5m_bio_04_horn_only_thumb.png]

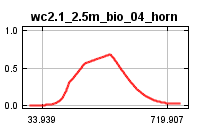

Supplement: Supplemental Information 6 [file peerj-08-9652-s006.zip › Data_S6_Ecological_Niche_Modeling/4_final_Maxent_analysis/output/plots/revoilii_12_wc2.1_2.5m_bio_04_horn_thumb.png]

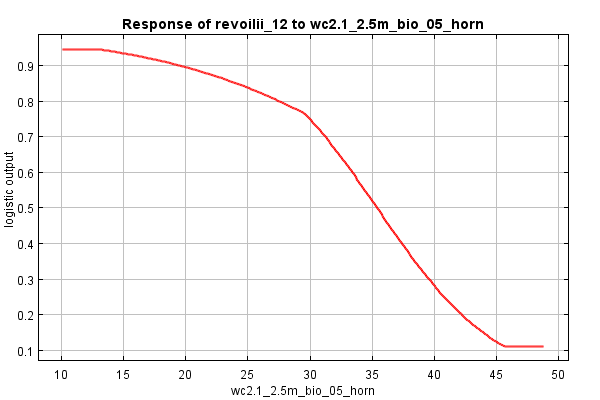

Supplement: Supplemental Information 6 [file peerj-08-9652-s006.zip › Data_S6_Ecological_Niche_Modeling/4_final_Maxent_analysis/output/plots/revoilii_12_wc2.1_2.5m_bio_05_horn.png]

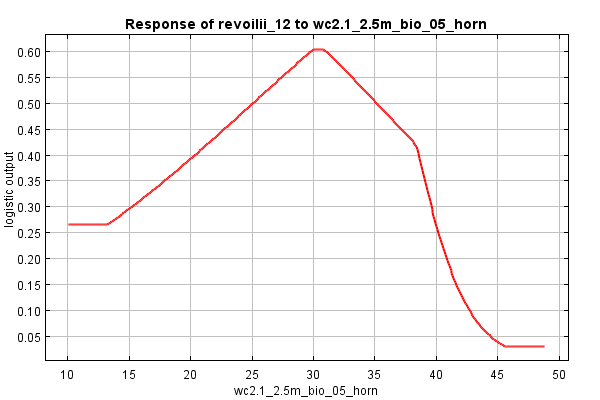

Supplement: Supplemental Information 6 [file peerj-08-9652-s006.zip › Data_S6_Ecological_Niche_Modeling/4_final_Maxent_analysis/output/plots/revoilii_12_wc2.1_2.5m_bio_05_horn_only.png]

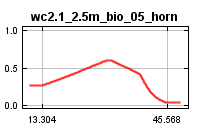

Supplement: Supplemental Information 6 [file peerj-08-9652-s006.zip › Data_S6_Ecological_Niche_Modeling/4_final_Maxent_analysis/output/plots/revoilii_12_wc2.1_2.5m_bio_05_horn_only_thumb.png]

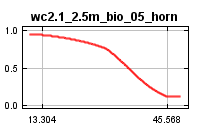

Supplement: Supplemental Information 6 [file peerj-08-9652-s006.zip › Data_S6_Ecological_Niche_Modeling/4_final_Maxent_analysis/output/plots/revoilii_12_wc2.1_2.5m_bio_05_horn_thumb.png]

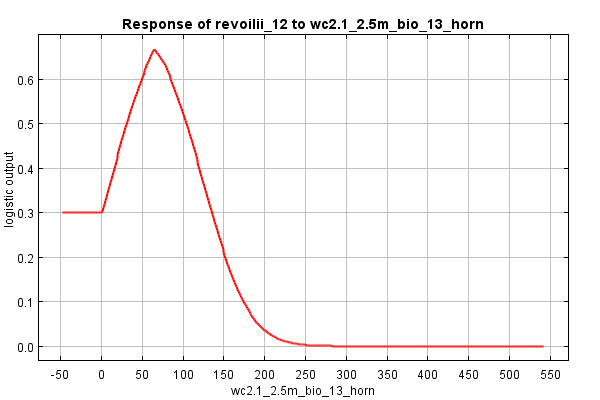

Supplement: Supplemental Information 6 [file peerj-08-9652-s006.zip › Data_S6_Ecological_Niche_Modeling/4_final_Maxent_analysis/output/plots/revoilii_12_wc2.1_2.5m_bio_13_horn.png]

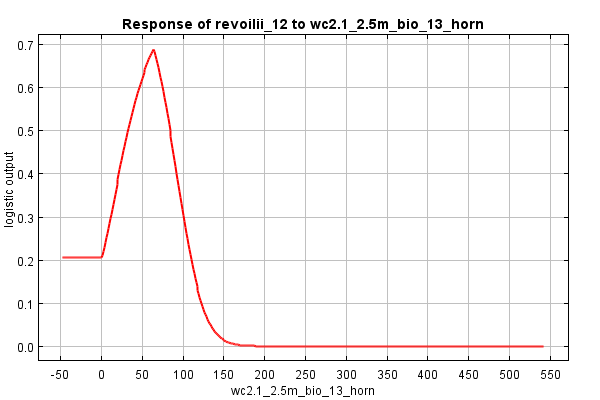

Supplement: Supplemental Information 6 [file peerj-08-9652-s006.zip › Data_S6_Ecological_Niche_Modeling/4_final_Maxent_analysis/output/plots/revoilii_12_wc2.1_2.5m_bio_13_horn_only.png]

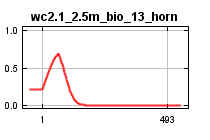

Supplement: Supplemental Information 6 [file peerj-08-9652-s006.zip › Data_S6_Ecological_Niche_Modeling/4_final_Maxent_analysis/output/plots/revoilii_12_wc2.1_2.5m_bio_13_horn_only_thumb.png]

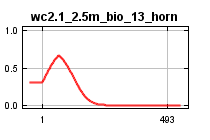

Supplement: Supplemental Information 6 [file peerj-08-9652-s006.zip › Data_S6_Ecological_Niche_Modeling/4_final_Maxent_analysis/output/plots/revoilii_12_wc2.1_2.5m_bio_13_horn_thumb.png]

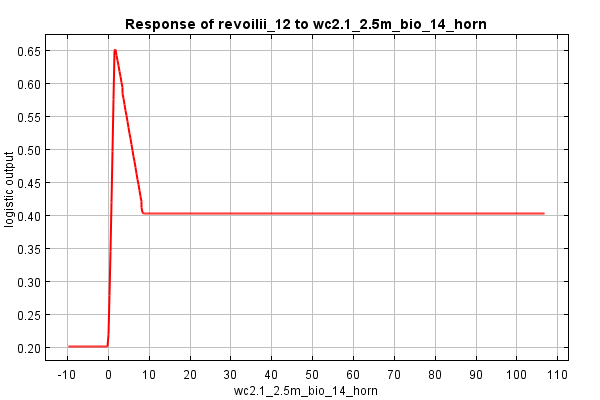

Supplement: Supplemental Information 6 [file peerj-08-9652-s006.zip › Data_S6_Ecological_Niche_Modeling/4_final_Maxent_analysis/output/plots/revoilii_12_wc2.1_2.5m_bio_14_horn.png]

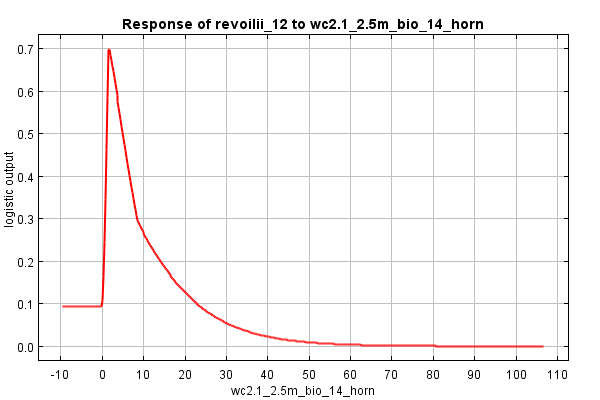

Supplement: Supplemental Information 6 [file peerj-08-9652-s006.zip › Data_S6_Ecological_Niche_Modeling/4_final_Maxent_analysis/output/plots/revoilii_12_wc2.1_2.5m_bio_14_horn_only.png]

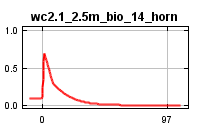

Supplement: Supplemental Information 6 [file peerj-08-9652-s006.zip › Data_S6_Ecological_Niche_Modeling/4_final_Maxent_analysis/output/plots/revoilii_12_wc2.1_2.5m_bio_14_horn_only_thumb.png]

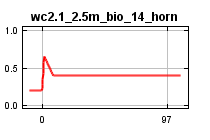

Supplement: Supplemental Information 6 [file peerj-08-9652-s006.zip › Data_S6_Ecological_Niche_Modeling/4_final_Maxent_analysis/output/plots/revoilii_12_wc2.1_2.5m_bio_14_horn_thumb.png]

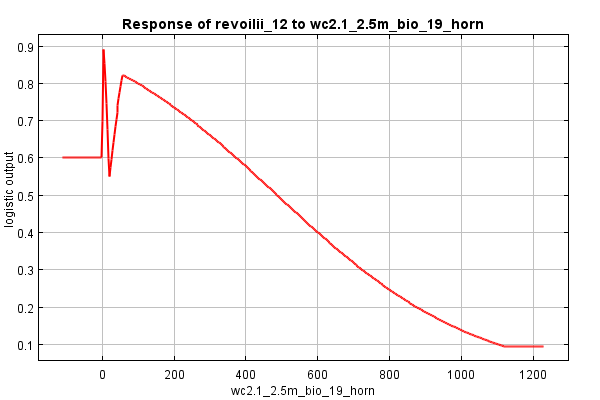

Supplement: Supplemental Information 6 [file peerj-08-9652-s006.zip › Data_S6_Ecological_Niche_Modeling/4_final_Maxent_analysis/output/plots/revoilii_12_wc2.1_2.5m_bio_19_horn.png]

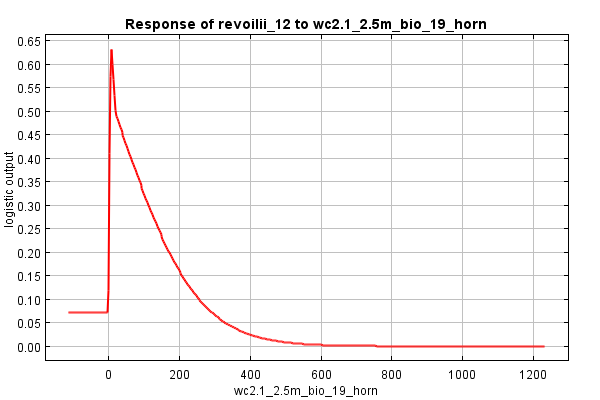

Supplement: Supplemental Information 6 [file peerj-08-9652-s006.zip › Data_S6_Ecological_Niche_Modeling/4_final_Maxent_analysis/output/plots/revoilii_12_wc2.1_2.5m_bio_19_horn_only.png]

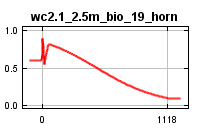

Supplement: Supplemental Information 6 [file peerj-08-9652-s006.zip › Data_S6_Ecological_Niche_Modeling/4_final_Maxent_analysis/output/plots/revoilii_12_wc2.1_2.5m_bio_19_horn_thumb.png]

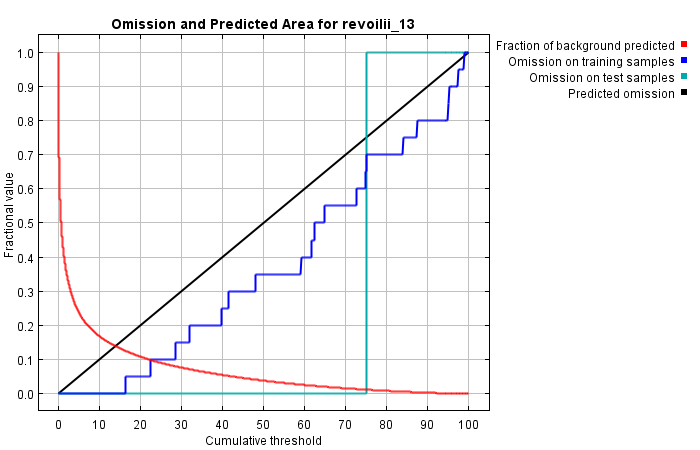

Supplement: Supplemental Information 6 [file peerj-08-9652-s006.zip › Data_S6_Ecological_Niche_Modeling/4_final_Maxent_analysis/output/plots/revoilii_13_omission.png]

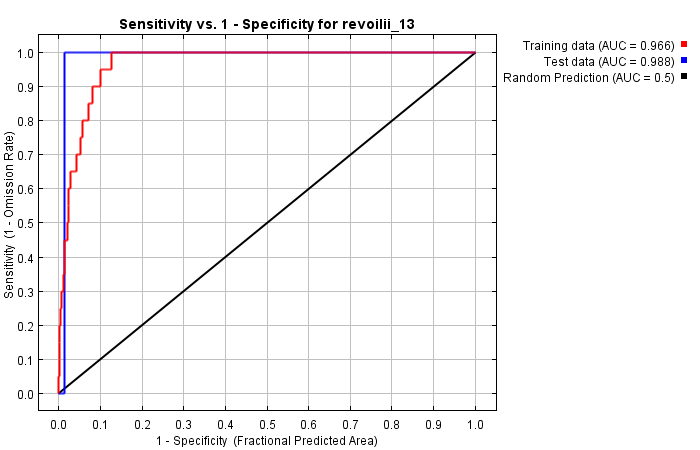

Supplement: Supplemental Information 6 [file peerj-08-9652-s006.zip › Data_S6_Ecological_Niche_Modeling/4_final_Maxent_analysis/output/plots/revoilii_13_roc.png]

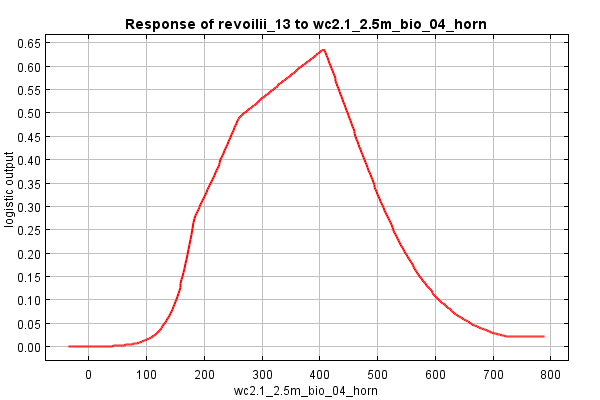

Supplement: Supplemental Information 6 [file peerj-08-9652-s006.zip › Data_S6_Ecological_Niche_Modeling/4_final_Maxent_analysis/output/plots/revoilii_13_wc2.1_2.5m_bio_04_horn.png]

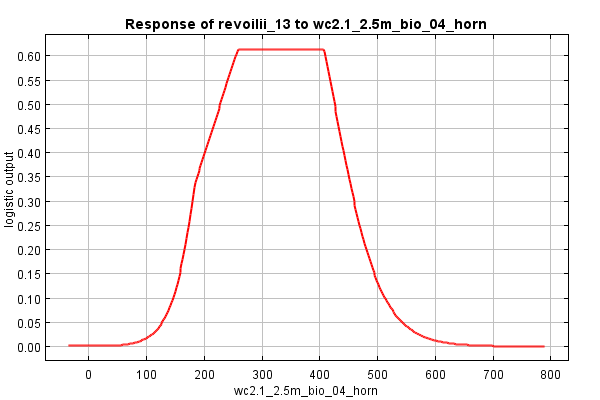

Supplement: Supplemental Information 6 [file peerj-08-9652-s006.zip › Data_S6_Ecological_Niche_Modeling/4_final_Maxent_analysis/output/plots/revoilii_13_wc2.1_2.5m_bio_04_horn_only.png]

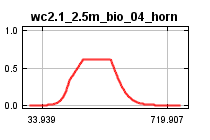

Supplement: Supplemental Information 6 [file peerj-08-9652-s006.zip › Data_S6_Ecological_Niche_Modeling/4_final_Maxent_analysis/output/plots/revoilii_13_wc2.1_2.5m_bio_04_horn_only_thumb.png]

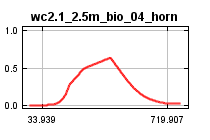

Supplement: Supplemental Information 6 [file peerj-08-9652-s006.zip › Data_S6_Ecological_Niche_Modeling/4_final_Maxent_analysis/output/plots/revoilii_13_wc2.1_2.5m_bio_04_horn_thumb.png]

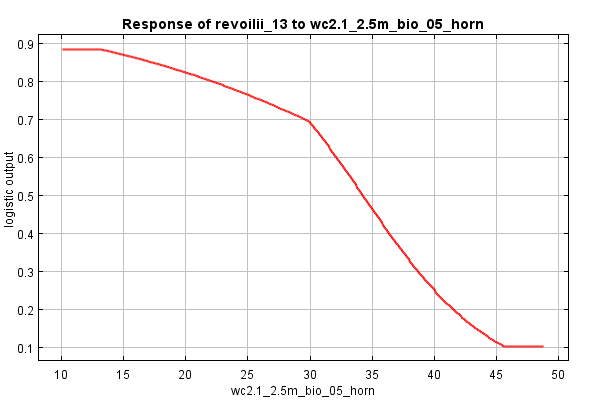

Supplement: Supplemental Information 6 [file peerj-08-9652-s006.zip › Data_S6_Ecological_Niche_Modeling/4_final_Maxent_analysis/output/plots/revoilii_13_wc2.1_2.5m_bio_05_horn.png]

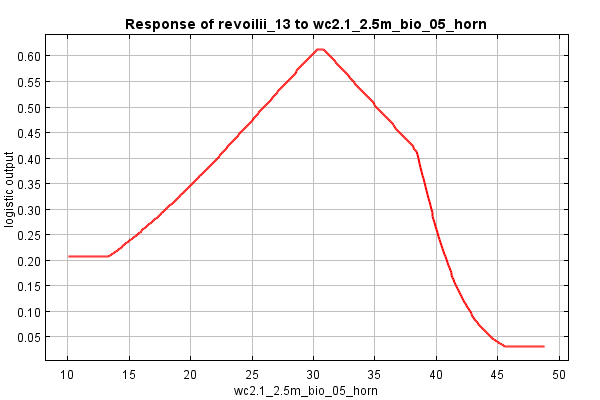

Supplement: Supplemental Information 6 [file peerj-08-9652-s006.zip › Data_S6_Ecological_Niche_Modeling/4_final_Maxent_analysis/output/plots/revoilii_13_wc2.1_2.5m_bio_05_horn_only.png]

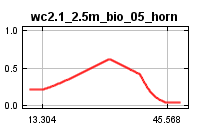

Supplement: Supplemental Information 6 [file peerj-08-9652-s006.zip › Data_S6_Ecological_Niche_Modeling/4_final_Maxent_analysis/output/plots/revoilii_13_wc2.1_2.5m_bio_05_horn_only_thumb.png]

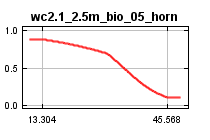

Supplement: Supplemental Information 6 [file peerj-08-9652-s006.zip › Data_S6_Ecological_Niche_Modeling/4_final_Maxent_analysis/output/plots/revoilii_13_wc2.1_2.5m_bio_05_horn_thumb.png]

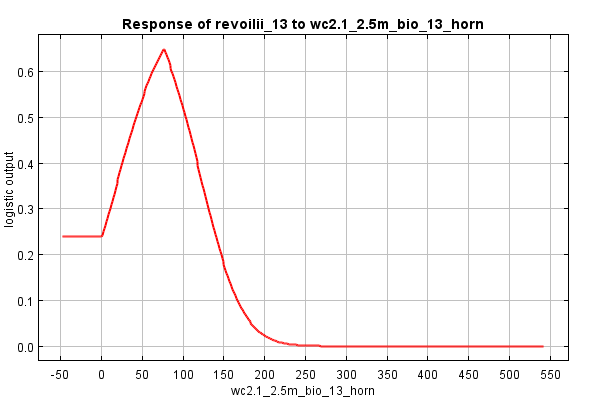

Supplement: Supplemental Information 6 [file peerj-08-9652-s006.zip › Data_S6_Ecological_Niche_Modeling/4_final_Maxent_analysis/output/plots/revoilii_13_wc2.1_2.5m_bio_13_horn.png]
