# Supplementary material for: New records of a lost species and a geographic range expansion for sengis in the Horn of Africa
Source: PeerJ. 2020 Aug 18;8:e9652. doi: 10.7717/peerj.9652 (PMC7441985; doi:10.7717/peerj.9652)

*New Records of a Lost Species and a Geographic Range Expansion for Sengis in the Horn of Africa*
*Heritage S, Rayaleh HA, Awaleh DG, Rathbun GB (2020)*

**Data S4. Phylogenetic Analyses (Bayesian-standard) of Individual Loci.**
*(this document has 15 pages)*

Locus: 12S (v1)
Genome: Mitochondrial
Partitions and Models: PartitionFinder Recommendation (see Data S3)
MrBayes Run Length: 2M generations
MrBayes Rel Burnin: 0.10
MrBayes ASDSF: 0.004889

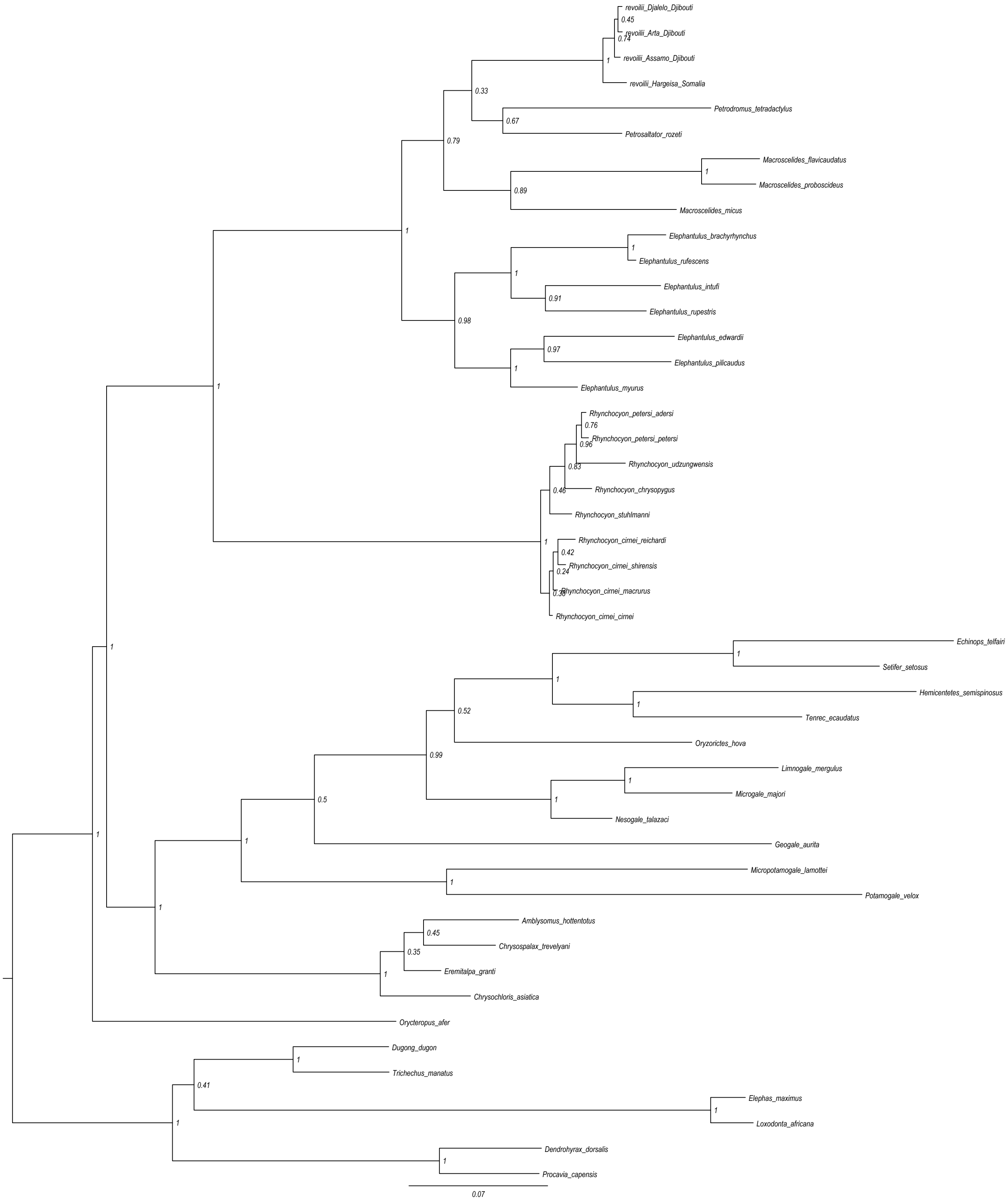

Locus: 12S (v2)
Genome: Mitochondrial
Partitions and Models: 1 partition, GTR+I+G
MrBayes Run Length: 2M generations
MrBayes Rel Burnin: 0.10
MrBayes ASDSF: 0.006080

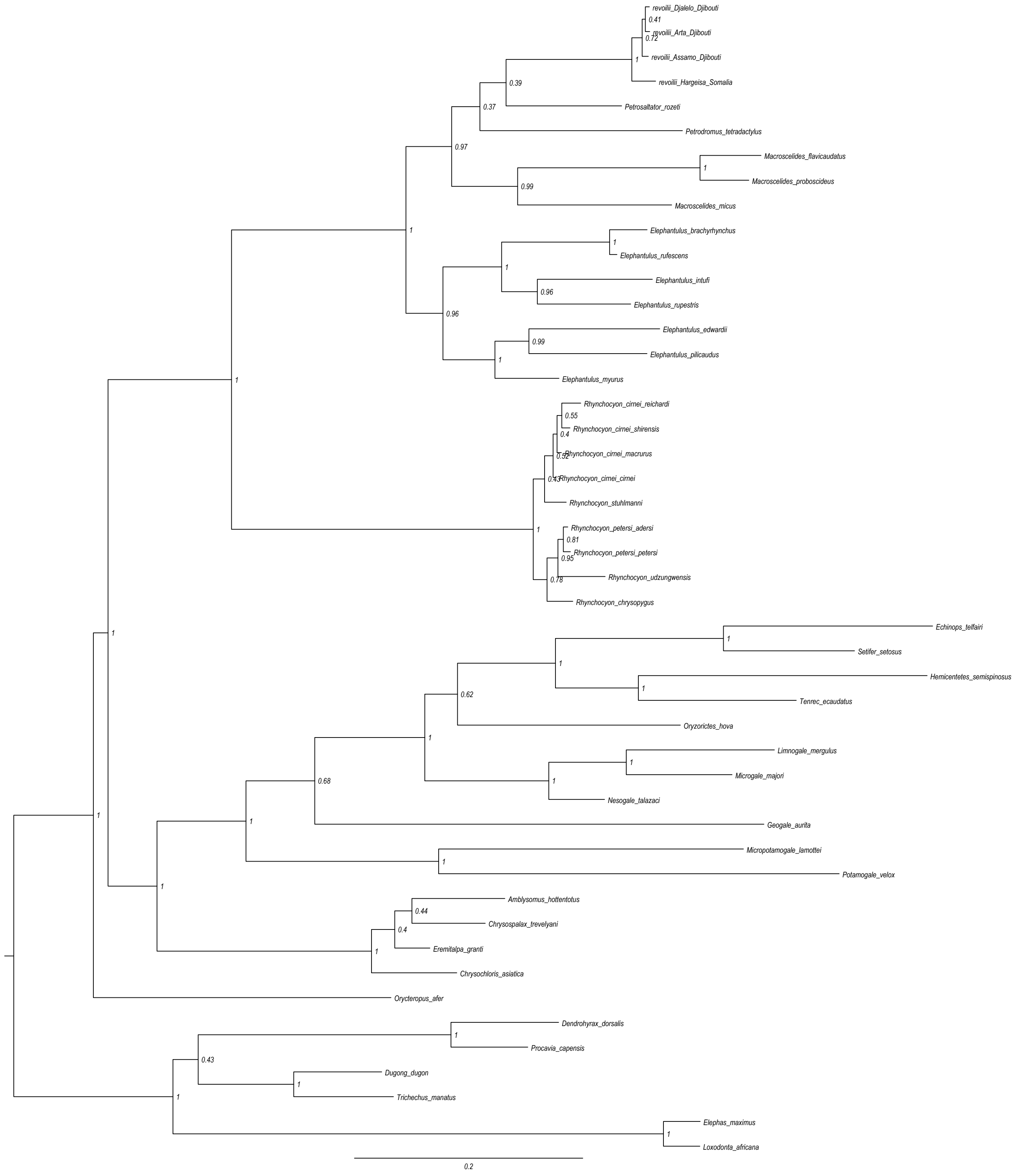

Locus: 16S (v1)
Genome: Mitochondrial
Partitions and Models: PartitionFinder Recommendation (see Data S3)
MrBayes Run Length: 2M generations
MrBayes Rel Burnin: 0.10
MrBayes ASDSF: 0.004978

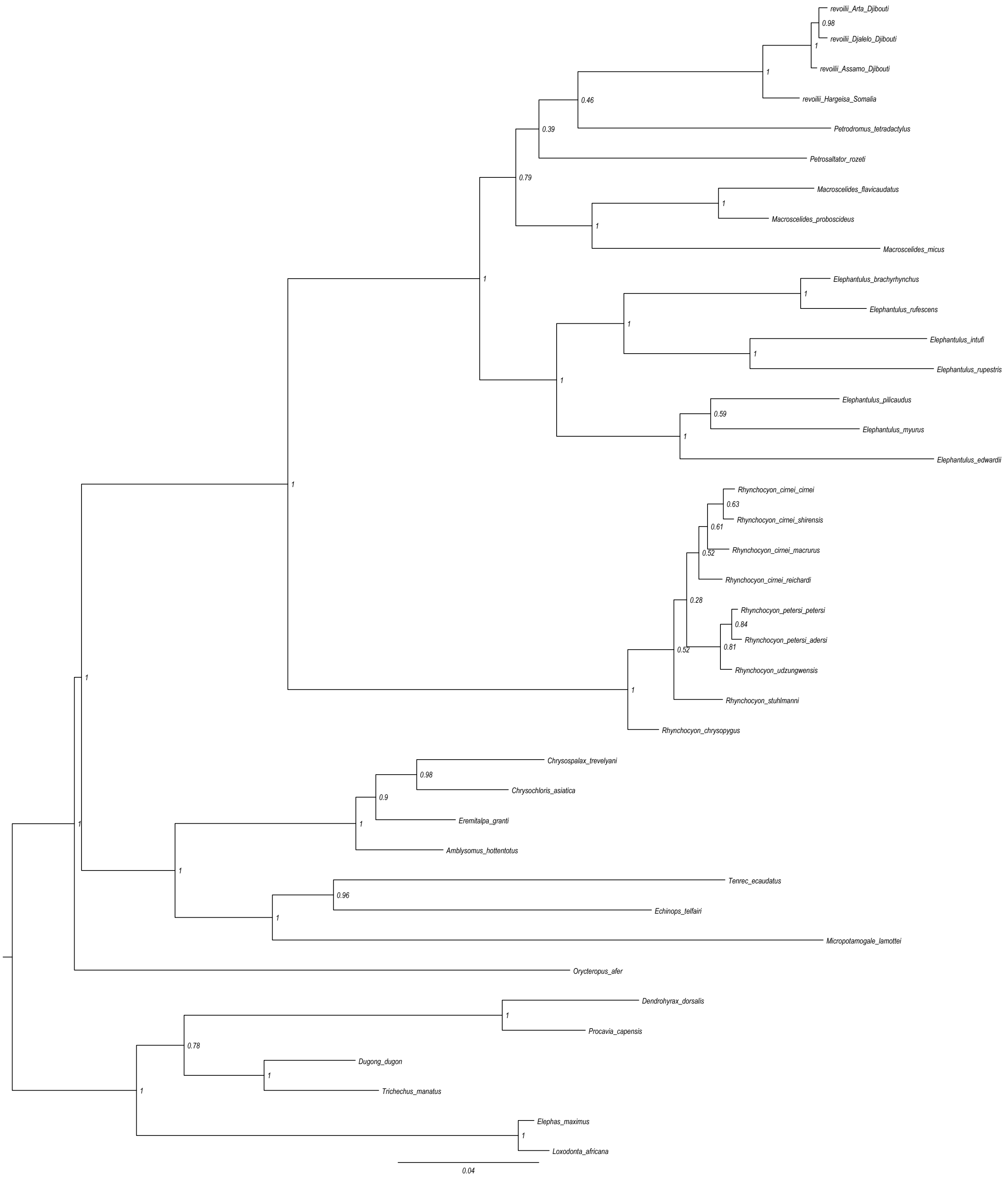

Locus: 16S (v2)
Genome: Mitochondrial
Partitions and Models: 1 partition, GTR+I+G
MrBayes Run Length: 2M generations
MrBayes Rel Burnin: 0.10
MrBayes ASDSF: 0.007772

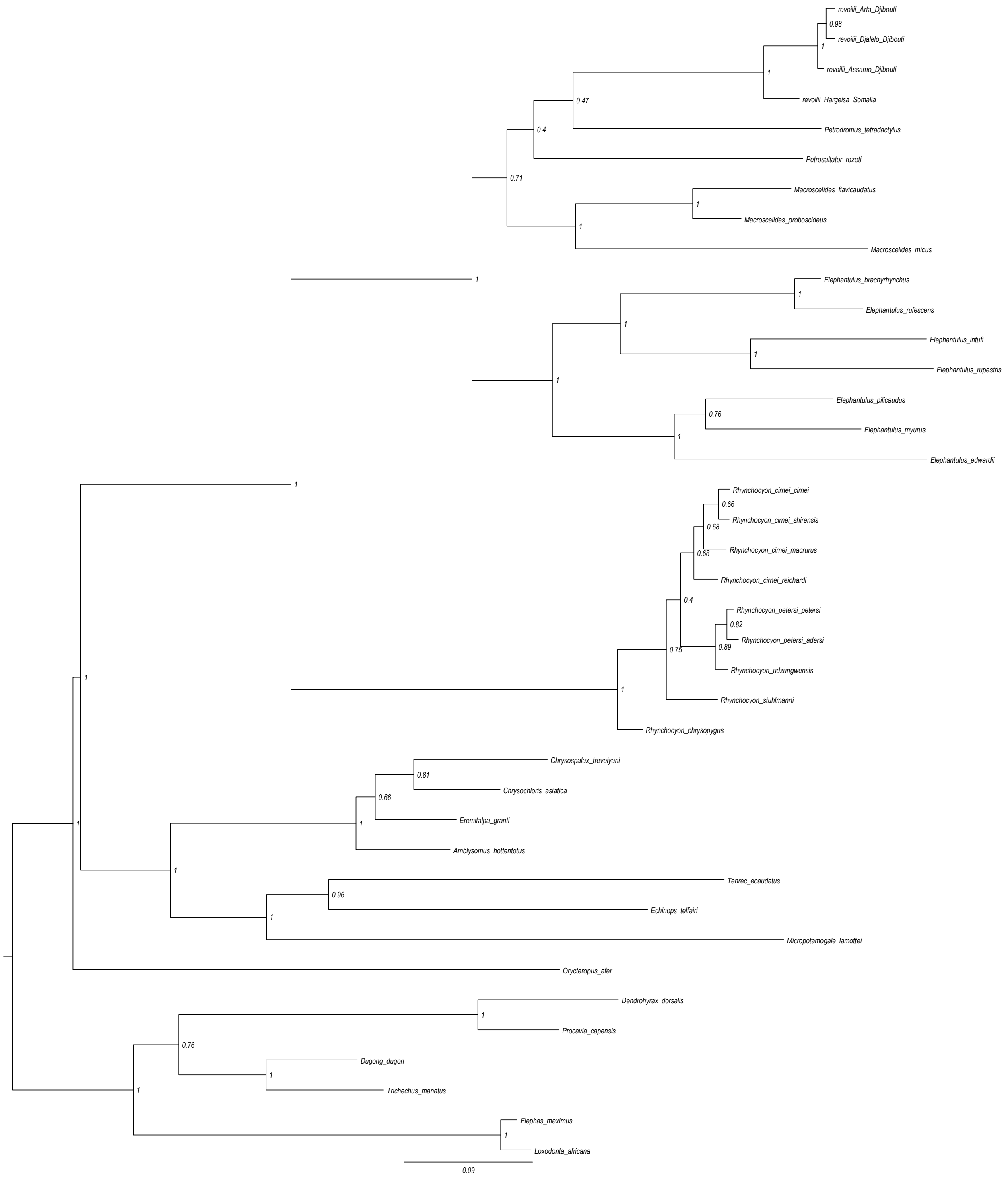

Locus: 12S&16S (v1)
Genome: Mitochondrial
Partitions and Models: PartitionFinder Recommendation (see Data S3)
MrBayes Run Length: 2M generations
MrBayes Rel Burnin: 0.10
MrBayes ASDSF: 0.003906

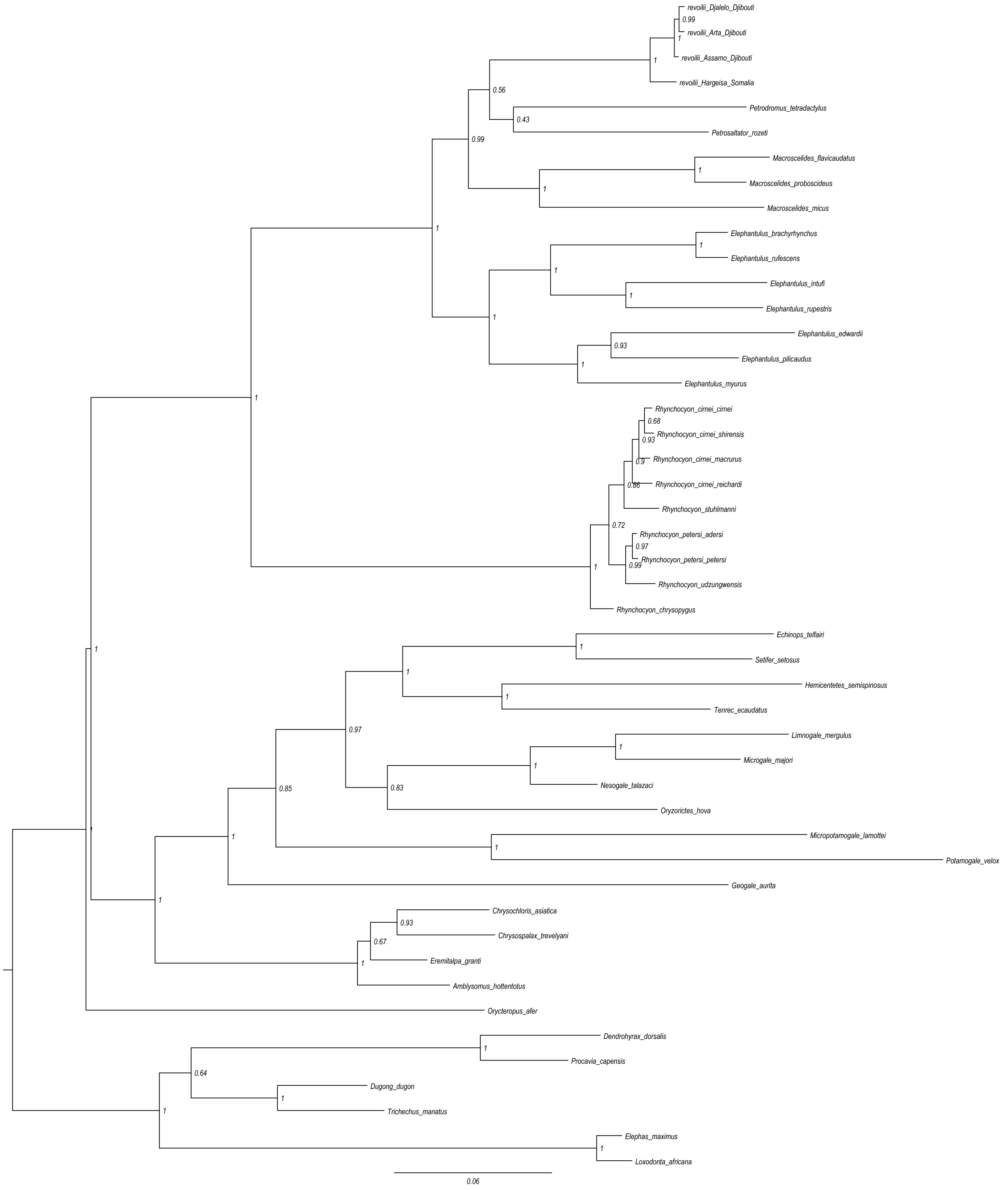

Locus: 12S&16S (v2)
Genome: Mitochondrial
Partitions and Models: 1 partition, GTR+I+G
MrBayes Run Length: 2M generations
MrBayes Rel Burnin: 0.10
MrBayes ASDSF: 0.003177

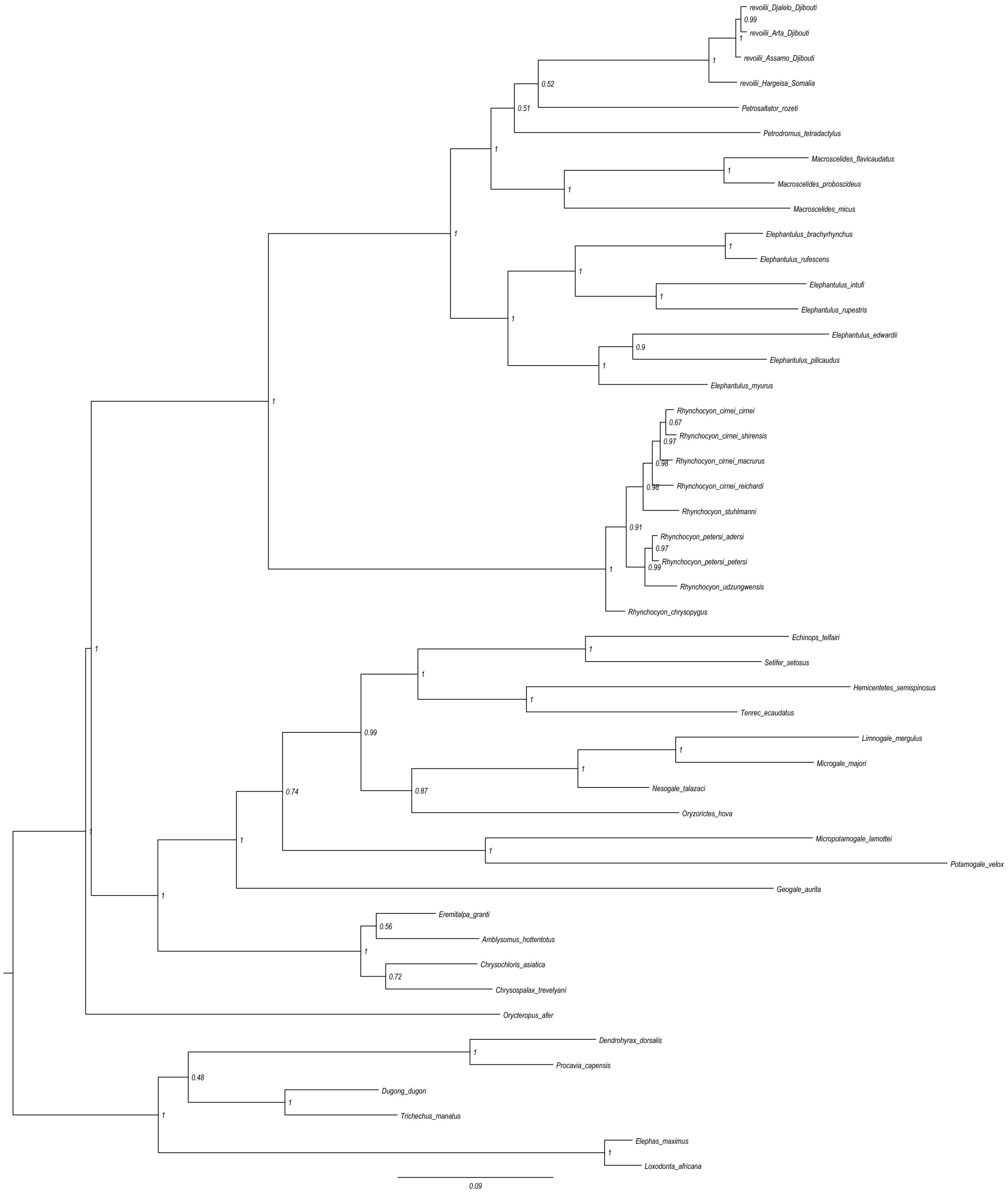

Locus: COX1 (v1)
Genome: Mitochondrial
Partitions and Models: PartitionFinder Recommendation (see Data S3)
MrBayes Run Length: 2M generations
MrBayes Rel Burnin: 0.10
MrBayes ASDSF: 0.001907

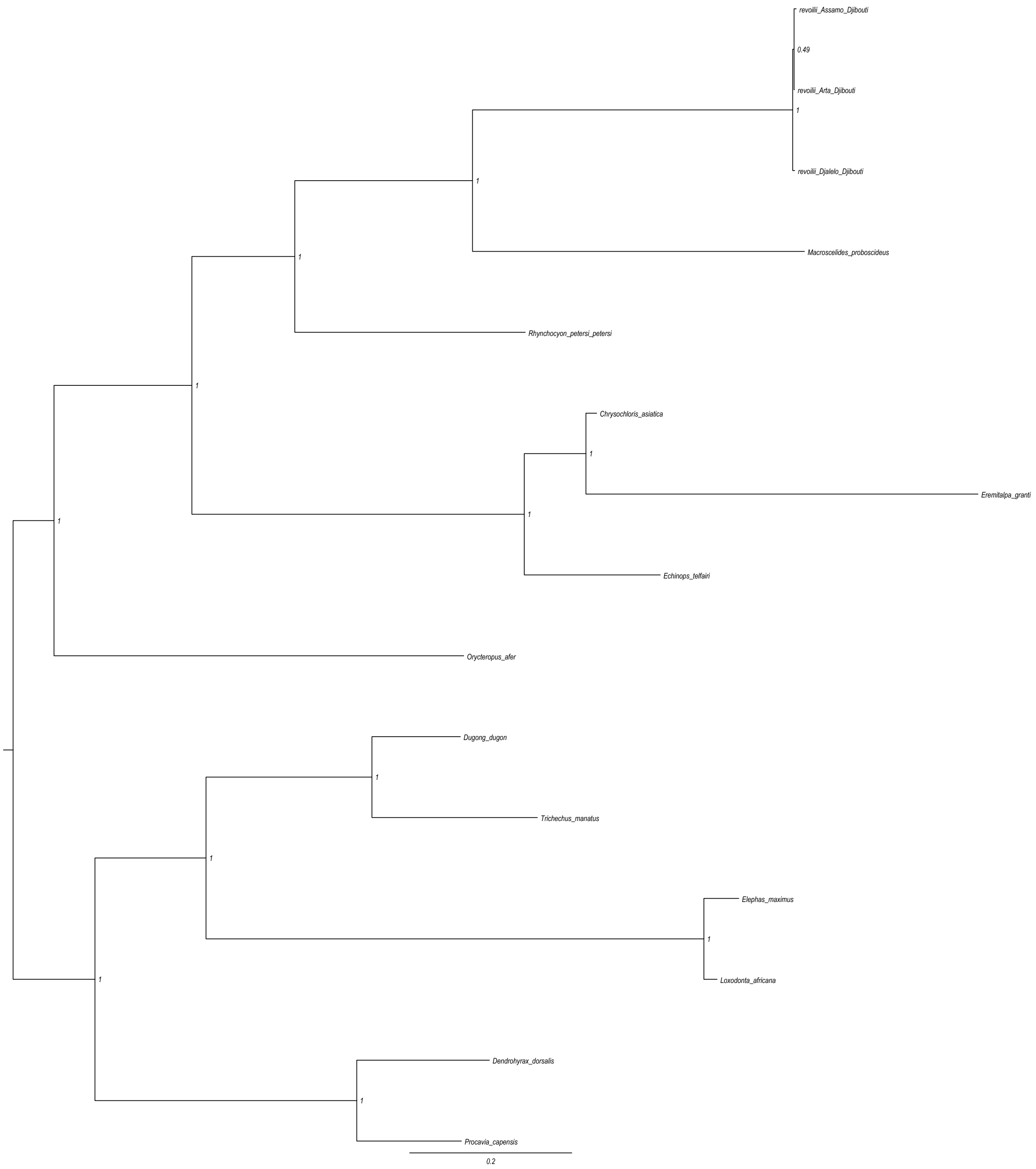

Locus: COX1 (v2)
Genome: Mitochondrial
Partitions and Models: 1 partition, GTR+I+G
MrBayes Run Length: 2M generations
MrBayes Rel Burnin: 0.10
MrBayes ASDSF: 0.002636

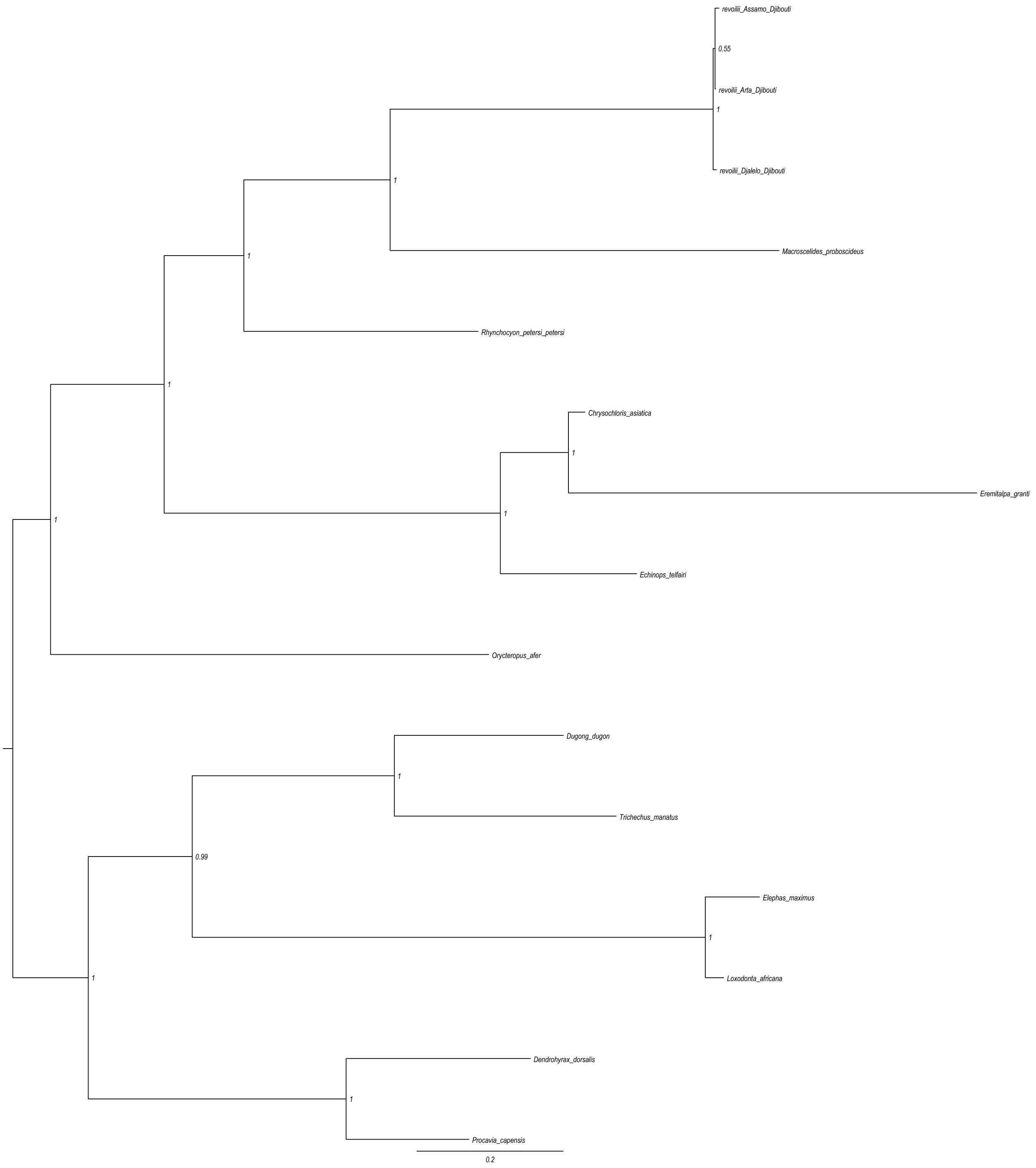

Locus: CYTB (v1)
Genome: Mitochondrial
Partitions and Models: PartitionFinder Recommendation (see Data S3)
MrBayes Run Length: 2M generations
MrBayes Rel Burnin: 0.10
MrBayes ASDSF: 0.004078

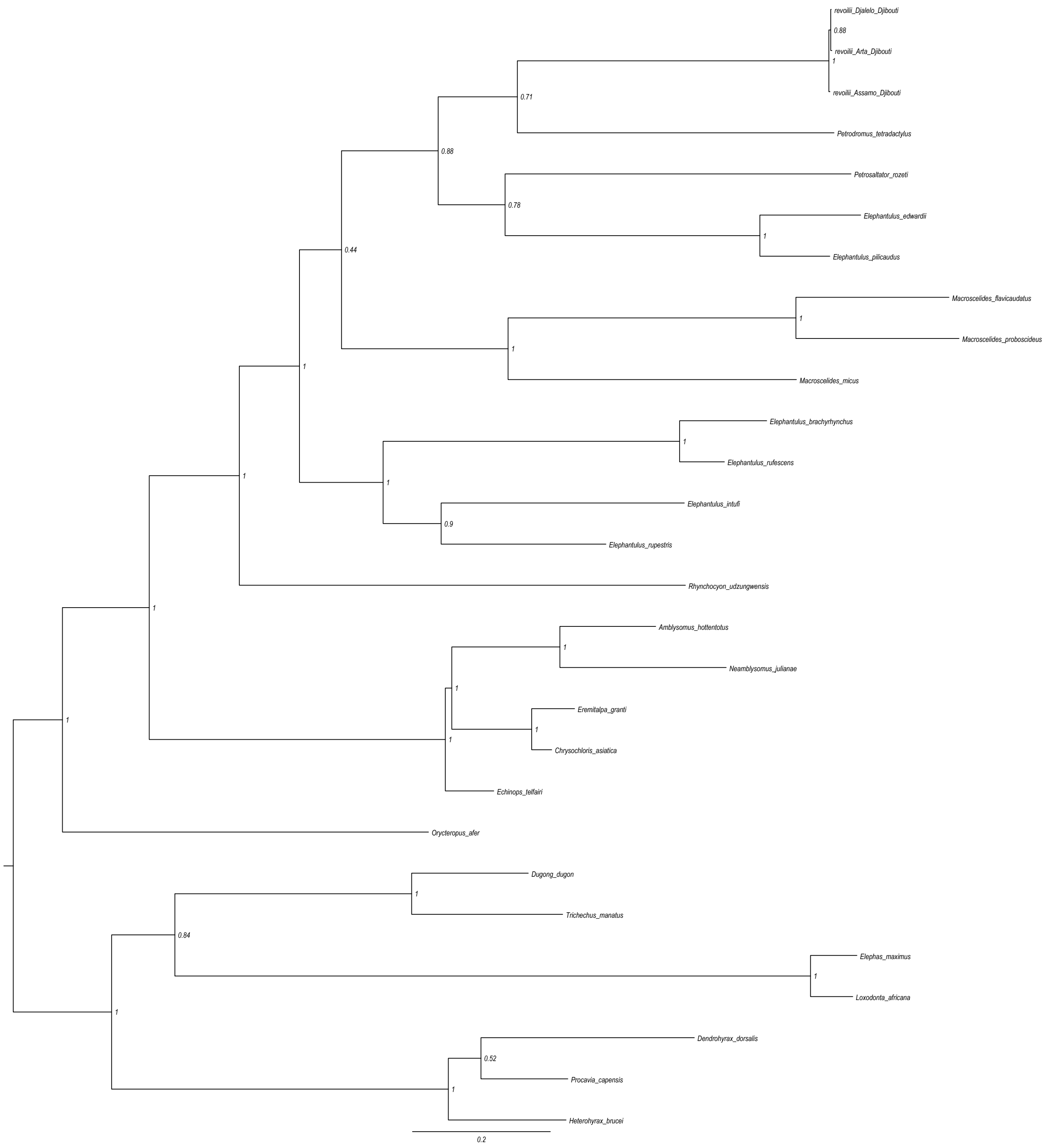

Locus: CYTB (v2)
Genome: Mitochondrial
Partitions and Models: 1 partition, GTR+I+G
MrBayes Run Length: 2M generations
MrBayes Rel Burnin: 0.10
MrBayes ASDSF: 0.004061

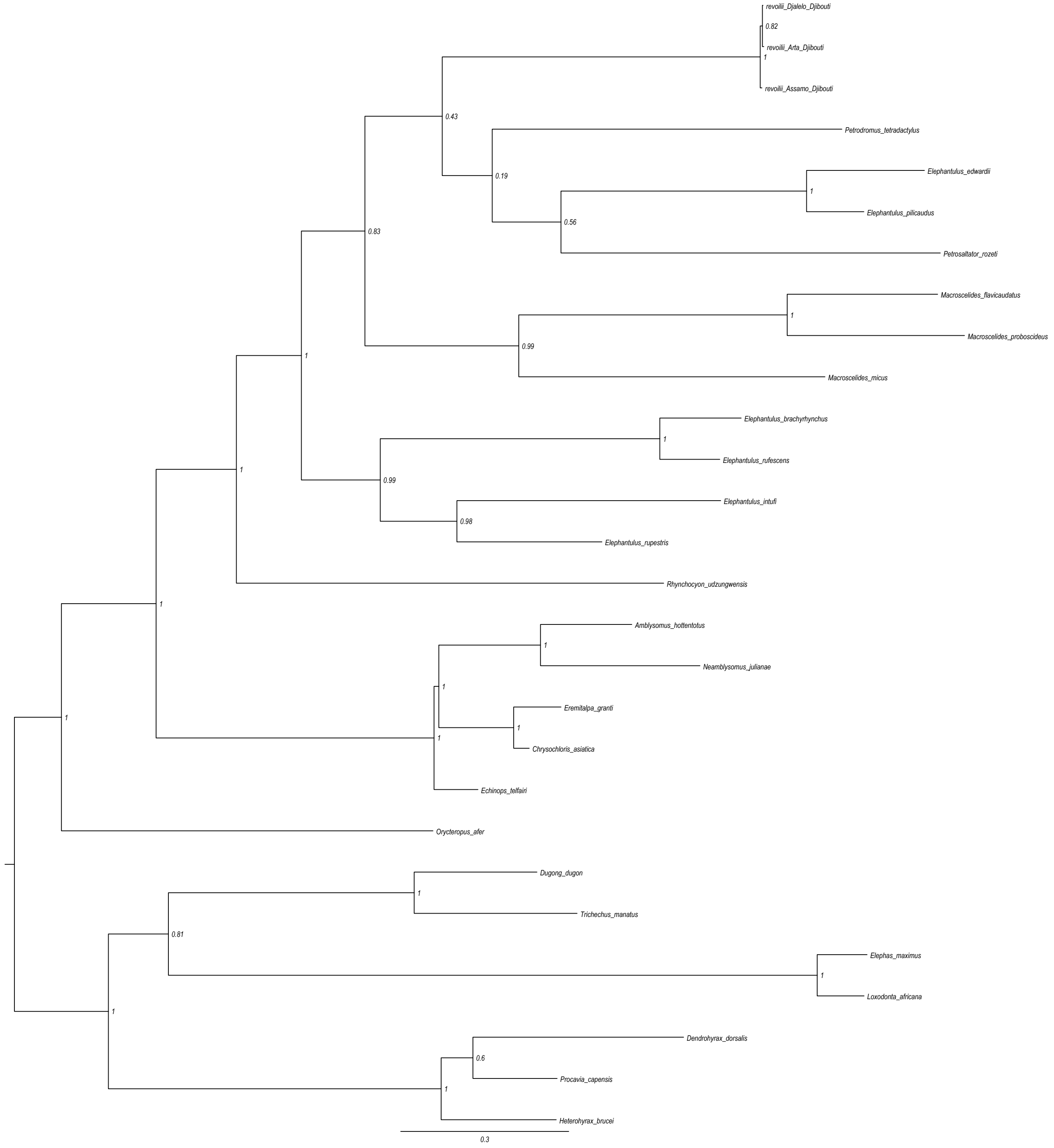

Locus: IRBP (v1)
Genome: Nuclear
Partitions and Models: PartitionFinder Recommendation (see Data S3)
MrBayes Run Length: 2M generations
MrBayes Rel Burnin: 0.10
MrBayes ASDSF: 0.005761

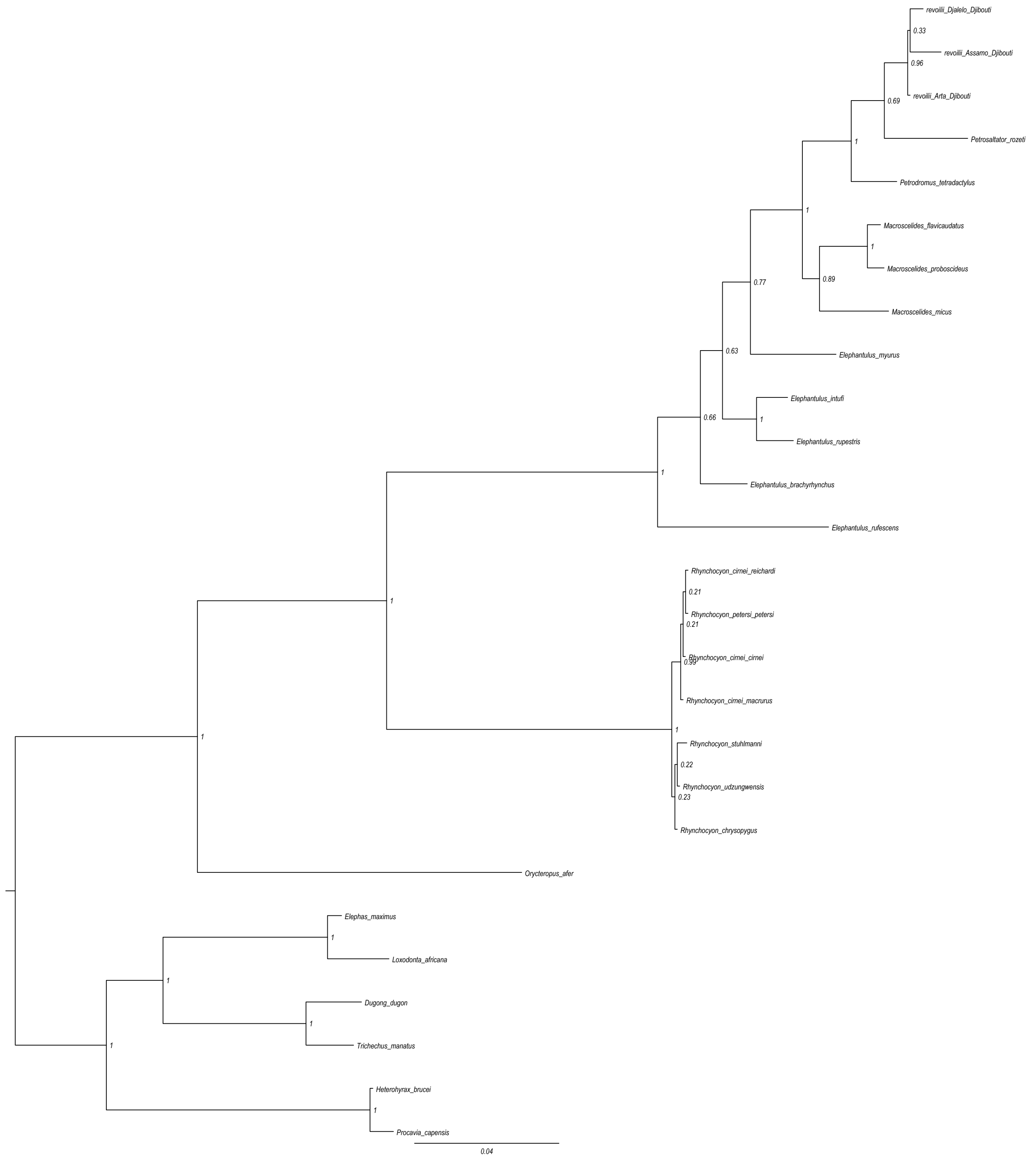

Locus: IRBP (v2)
Genome: Nuclear
Partitions and Models: 1 partition, GTR+I+G
MrBayes Run Length: 2M generations
MrBayes Rel Burnin: 0.10
MrBayes ASDSF: 0.007460

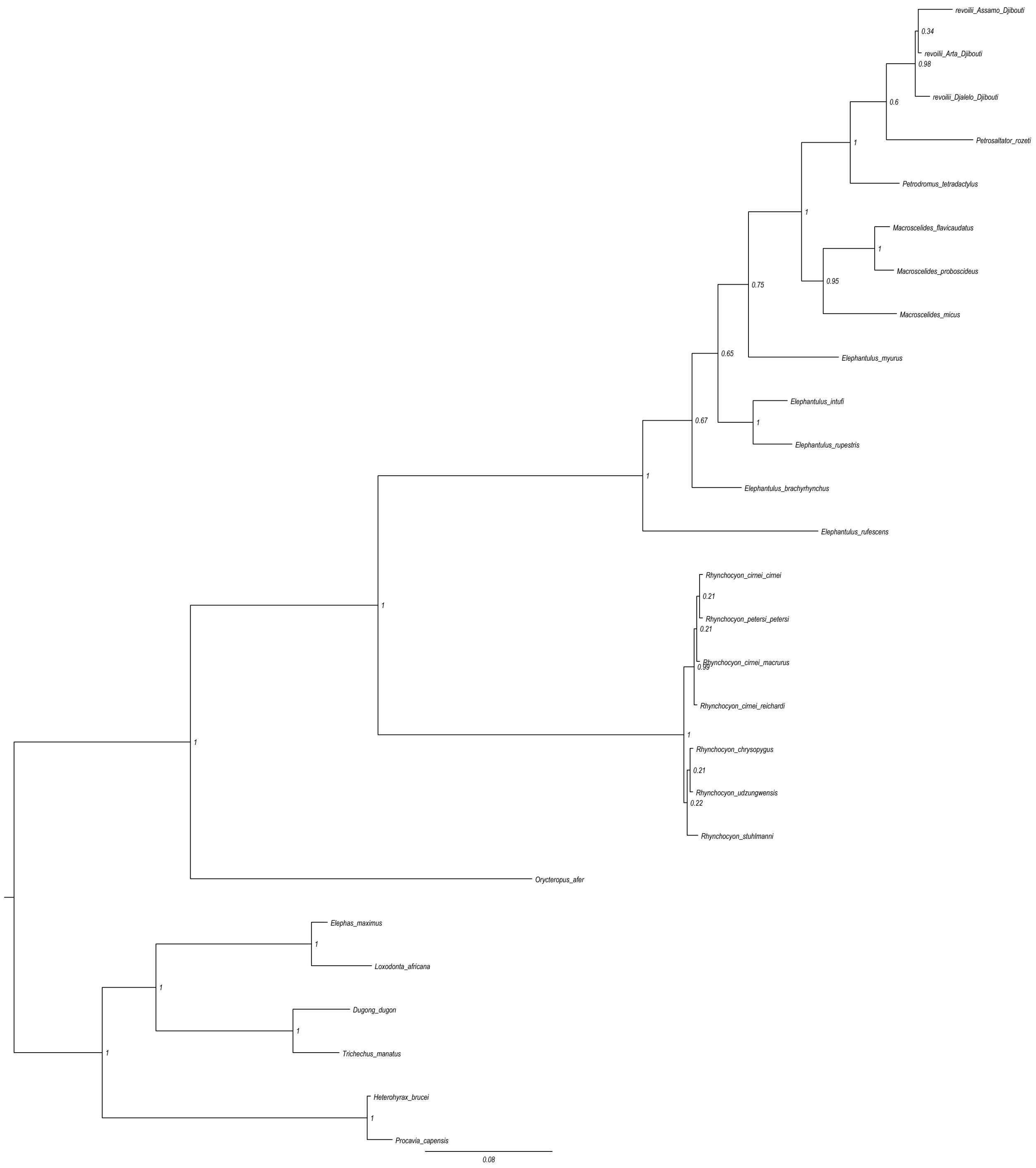

Locus: VWF (v1)
Genome: Nuclear
Partitions and Models: PartitionFinder Recommendation (see Data S3)
MrBayes Run Length: 2M generations
MrBayes Rel Burnin: 0.10
MrBayes ASDSF: 0.003759

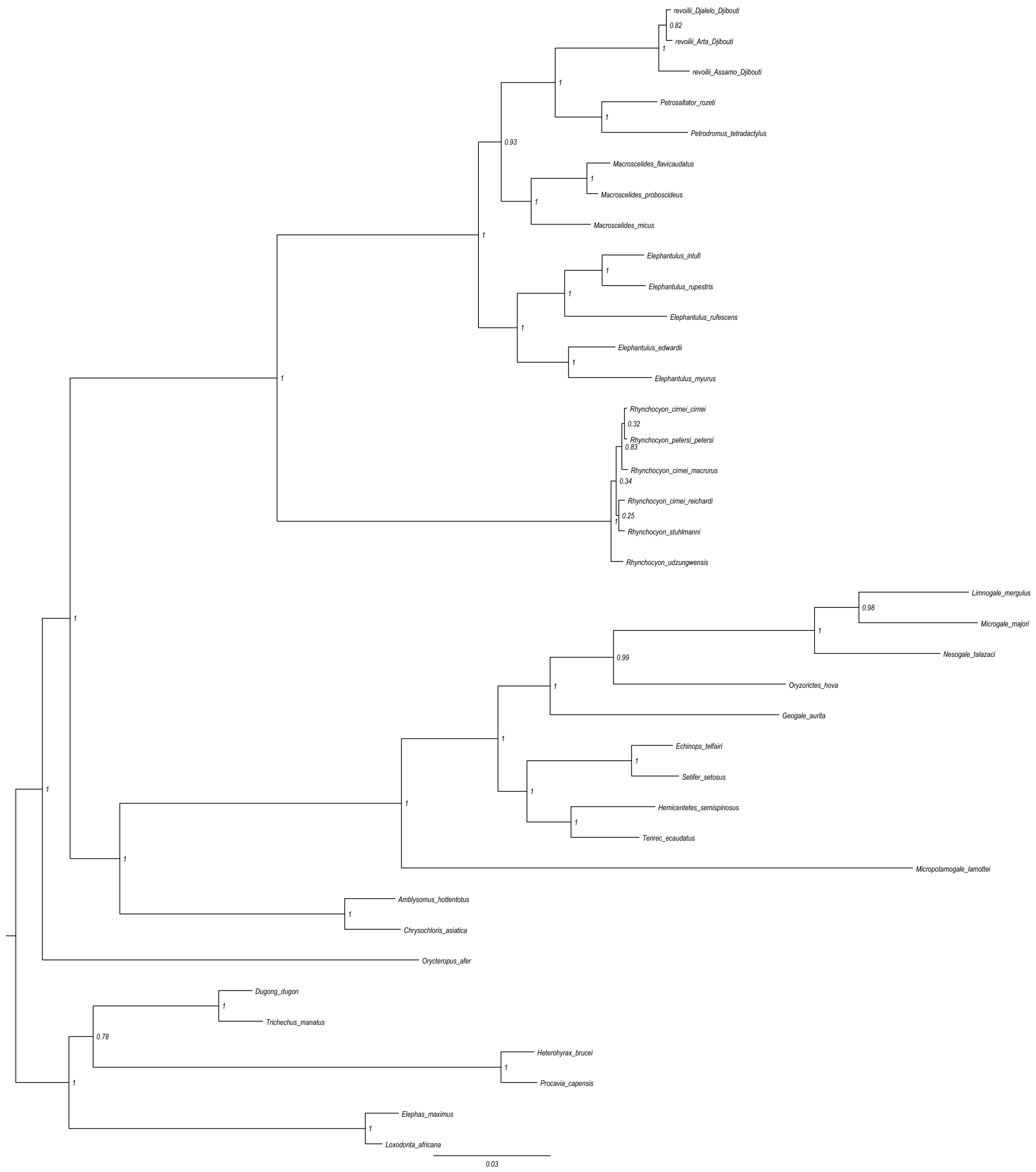

Locus: VWF (v2)
Genome: Nuclear
Partitions and Models: 1 partition, GTR+I+G
MrBayes Run Length: 2M generations
MrBayes Rel Burnin: 0.10
MrBayes ASDSF: 0.002890

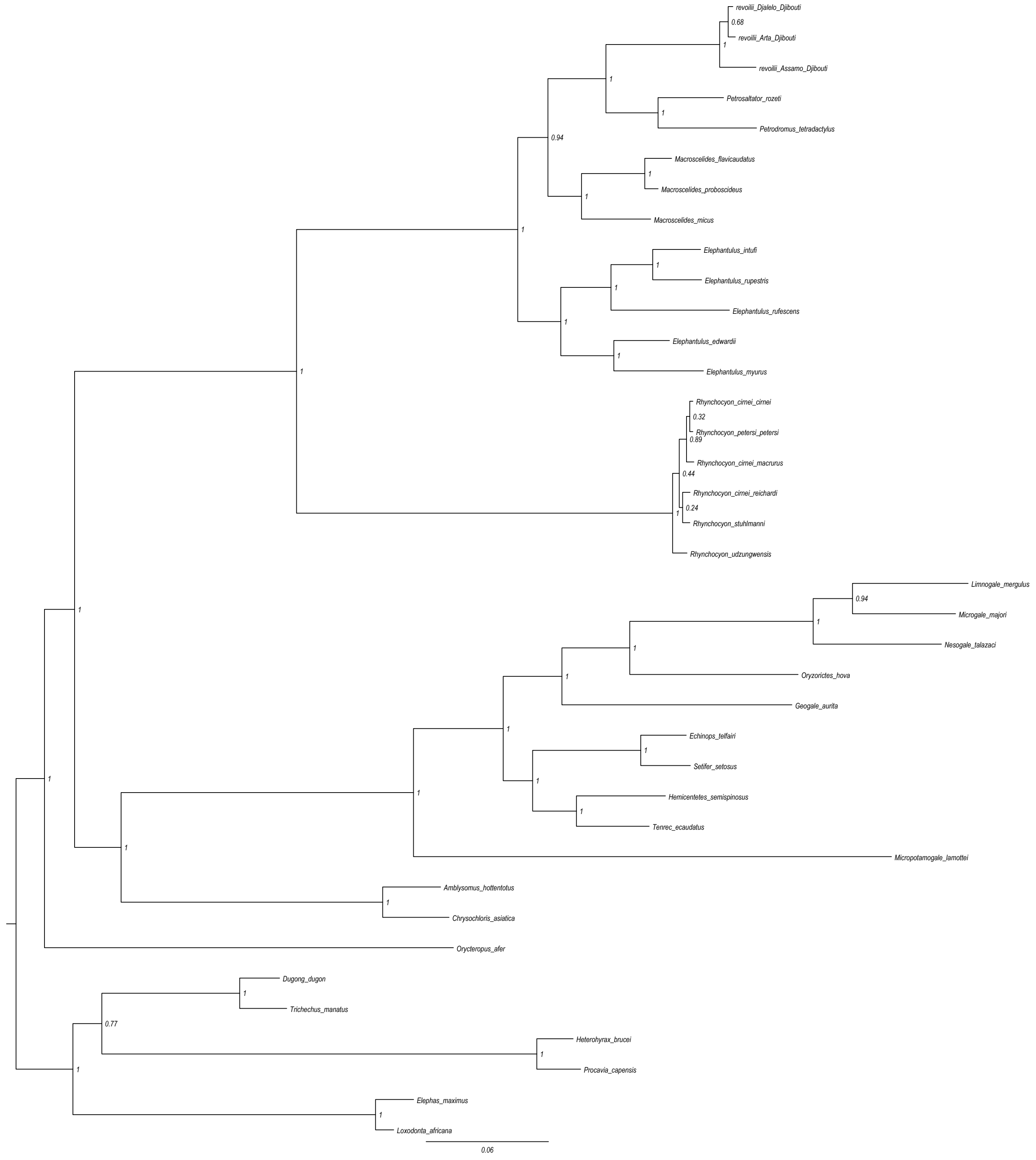

Supplement: Supplemental Information 4 [file peerj-08-9652-s004.zip › Data_S4_Phylogenetic_Analyses_of_Individual_Loci/Data_S4_Phylogenetic_Analyses_of_Individual_Loci.pdf]
