# Supplementary figures and images for: New records of a lost species and a geographic range expansion for sengis in the Horn of Africa (part 2 of 2)
Source: PeerJ. 2020 Aug 18;8:e9652. doi: 10.7717/peerj.9652 (PMC7441985; doi:10.7717/peerj.9652)

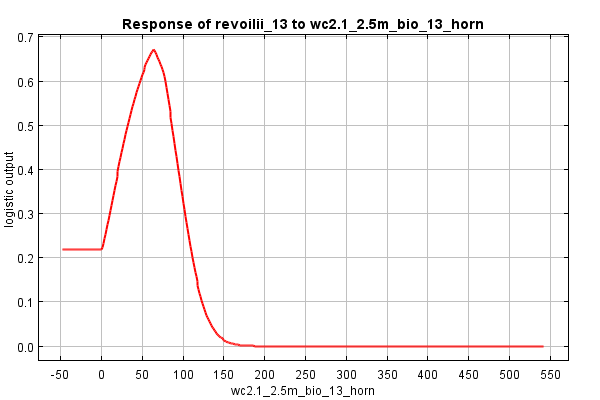

Supplement: Supplemental Information 6 [file peerj-08-9652-s006.zip › Data_S6_Ecological_Niche_Modeling/4_final_Maxent_analysis/output/plots/revoilii_13_wc2.1_2.5m_bio_13_horn_only.png]

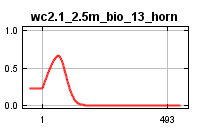

Supplement: Supplemental Information 6 [file peerj-08-9652-s006.zip › Data_S6_Ecological_Niche_Modeling/4_final_Maxent_analysis/output/plots/revoilii_13_wc2.1_2.5m_bio_13_horn_only_thumb.png]

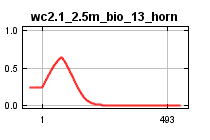

Supplement: Supplemental Information 6 [file peerj-08-9652-s006.zip › Data_S6_Ecological_Niche_Modeling/4_final_Maxent_analysis/output/plots/revoilii_13_wc2.1_2.5m_bio_13_horn_thumb.png]

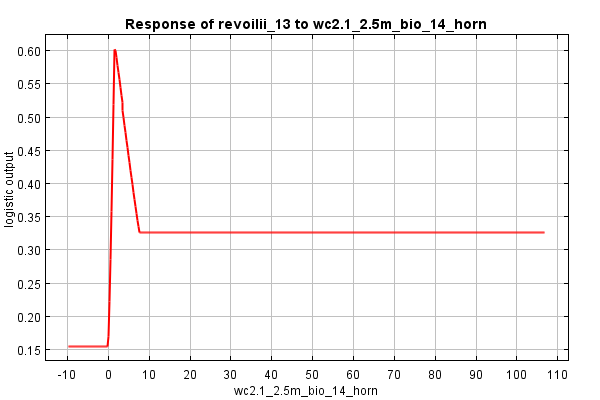

Supplement: Supplemental Information 6 [file peerj-08-9652-s006.zip › Data_S6_Ecological_Niche_Modeling/4_final_Maxent_analysis/output/plots/revoilii_13_wc2.1_2.5m_bio_14_horn.png]

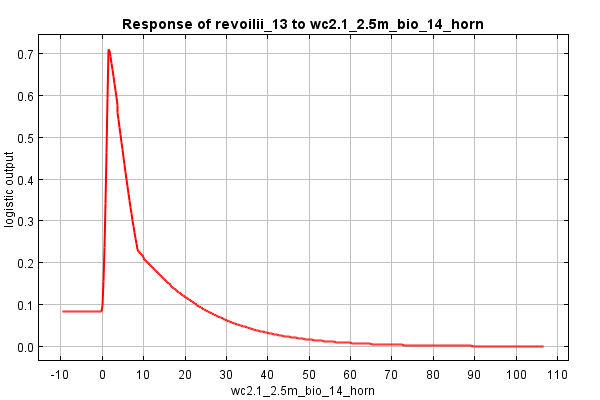

Supplement: Supplemental Information 6 [file peerj-08-9652-s006.zip › Data_S6_Ecological_Niche_Modeling/4_final_Maxent_analysis/output/plots/revoilii_13_wc2.1_2.5m_bio_14_horn_only.png]

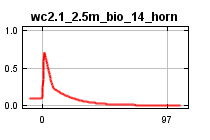

Supplement: Supplemental Information 6 [file peerj-08-9652-s006.zip › Data_S6_Ecological_Niche_Modeling/4_final_Maxent_analysis/output/plots/revoilii_13_wc2.1_2.5m_bio_14_horn_only_thumb.png]

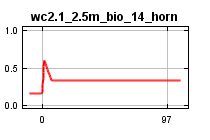

Supplement: Supplemental Information 6 [file peerj-08-9652-s006.zip › Data_S6_Ecological_Niche_Modeling/4_final_Maxent_analysis/output/plots/revoilii_13_wc2.1_2.5m_bio_14_horn_thumb.png]

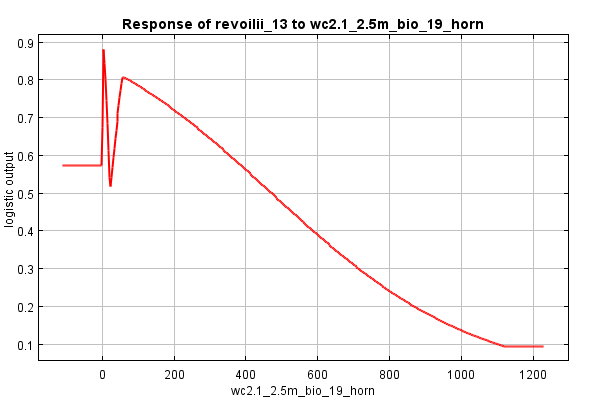

Supplement: Supplemental Information 6 [file peerj-08-9652-s006.zip › Data_S6_Ecological_Niche_Modeling/4_final_Maxent_analysis/output/plots/revoilii_13_wc2.1_2.5m_bio_19_horn.png]

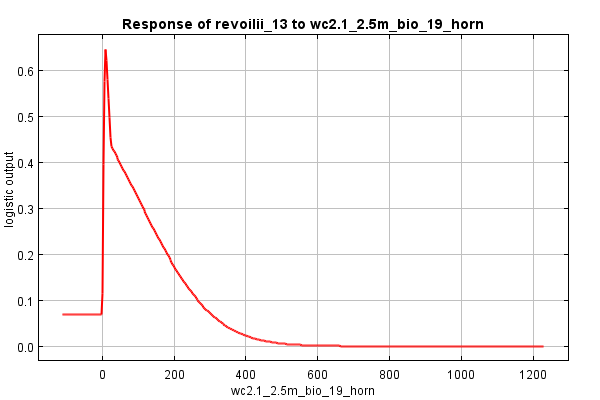

Supplement: Supplemental Information 6 [file peerj-08-9652-s006.zip › Data_S6_Ecological_Niche_Modeling/4_final_Maxent_analysis/output/plots/revoilii_13_wc2.1_2.5m_bio_19_horn_only.png]

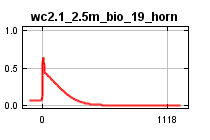

Supplement: Supplemental Information 6 [file peerj-08-9652-s006.zip › Data_S6_Ecological_Niche_Modeling/4_final_Maxent_analysis/output/plots/revoilii_13_wc2.1_2.5m_bio_19_horn_only_thumb.png]

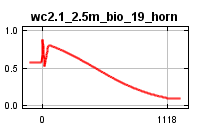

Supplement: Supplemental Information 6 [file peerj-08-9652-s006.zip › Data_S6_Ecological_Niche_Modeling/4_final_Maxent_analysis/output/plots/revoilii_13_wc2.1_2.5m_bio_19_horn_thumb.png]

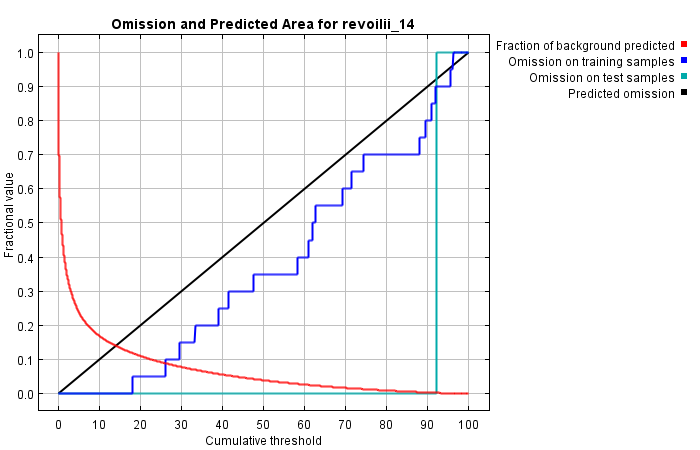

Supplement: Supplemental Information 6 [file peerj-08-9652-s006.zip › Data_S6_Ecological_Niche_Modeling/4_final_Maxent_analysis/output/plots/revoilii_14_omission.png]

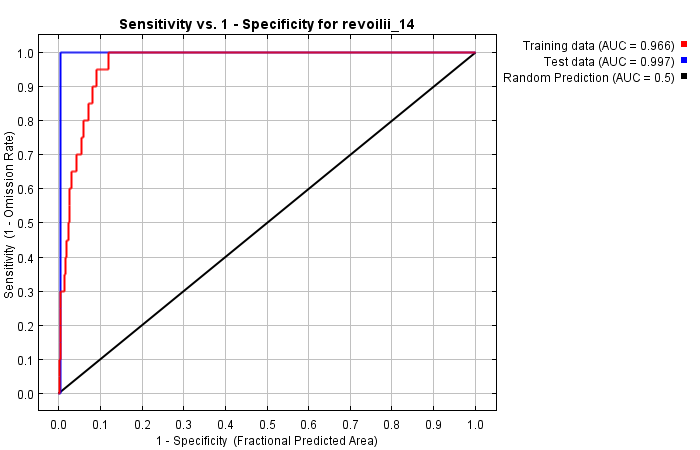

Supplement: Supplemental Information 6 [file peerj-08-9652-s006.zip › Data_S6_Ecological_Niche_Modeling/4_final_Maxent_analysis/output/plots/revoilii_14_roc.png]

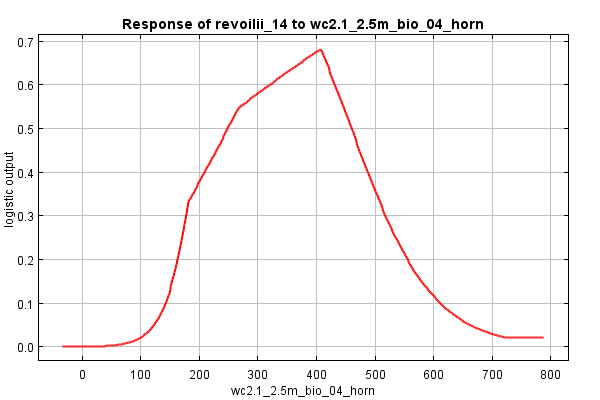

Supplement: Supplemental Information 6 [file peerj-08-9652-s006.zip › Data_S6_Ecological_Niche_Modeling/4_final_Maxent_analysis/output/plots/revoilii_14_wc2.1_2.5m_bio_04_horn.png]

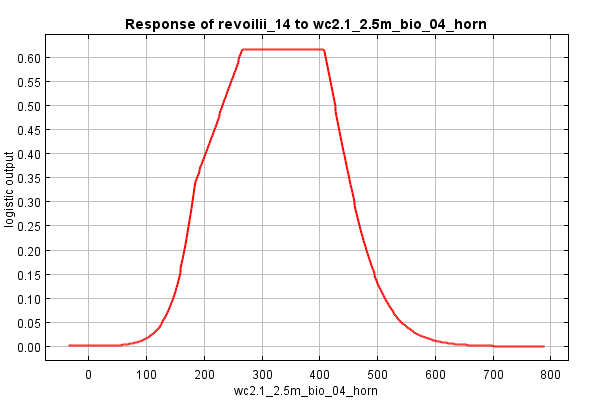

Supplement: Supplemental Information 6 [file peerj-08-9652-s006.zip › Data_S6_Ecological_Niche_Modeling/4_final_Maxent_analysis/output/plots/revoilii_14_wc2.1_2.5m_bio_04_horn_only.png]

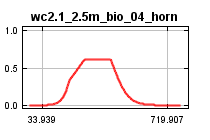

Supplement: Supplemental Information 6 [file peerj-08-9652-s006.zip › Data_S6_Ecological_Niche_Modeling/4_final_Maxent_analysis/output/plots/revoilii_14_wc2.1_2.5m_bio_04_horn_only_thumb.png]

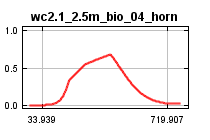

Supplement: Supplemental Information 6 [file peerj-08-9652-s006.zip › Data_S6_Ecological_Niche_Modeling/4_final_Maxent_analysis/output/plots/revoilii_14_wc2.1_2.5m_bio_04_horn_thumb.png]

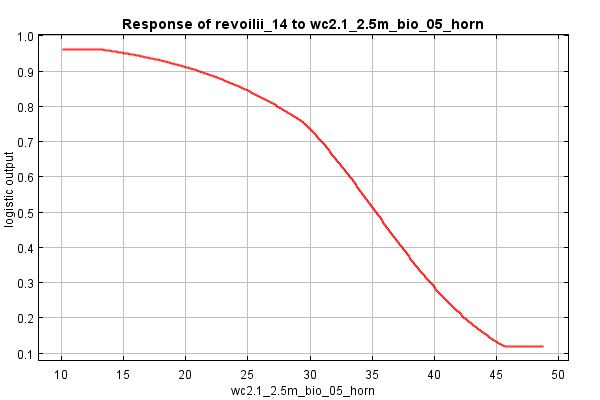

Supplement: Supplemental Information 6 [file peerj-08-9652-s006.zip › Data_S6_Ecological_Niche_Modeling/4_final_Maxent_analysis/output/plots/revoilii_14_wc2.1_2.5m_bio_05_horn.png]

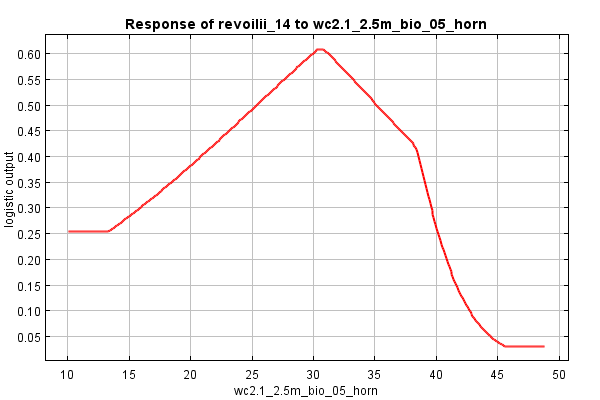

Supplement: Supplemental Information 6 [file peerj-08-9652-s006.zip › Data_S6_Ecological_Niche_Modeling/4_final_Maxent_analysis/output/plots/revoilii_14_wc2.1_2.5m_bio_05_horn_only.png]

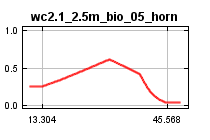

Supplement: Supplemental Information 6 [file peerj-08-9652-s006.zip › Data_S6_Ecological_Niche_Modeling/4_final_Maxent_analysis/output/plots/revoilii_14_wc2.1_2.5m_bio_05_horn_only_thumb.png]

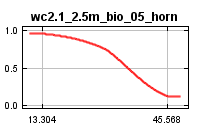

Supplement: Supplemental Information 6 [file peerj-08-9652-s006.zip › Data_S6_Ecological_Niche_Modeling/4_final_Maxent_analysis/output/plots/revoilii_14_wc2.1_2.5m_bio_05_horn_thumb.png]

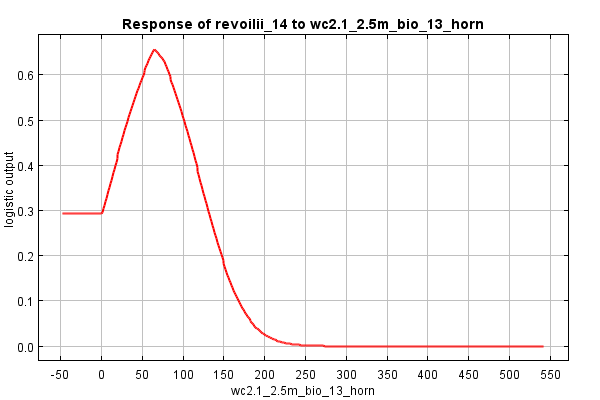

Supplement: Supplemental Information 6 [file peerj-08-9652-s006.zip › Data_S6_Ecological_Niche_Modeling/4_final_Maxent_analysis/output/plots/revoilii_14_wc2.1_2.5m_bio_13_horn.png]

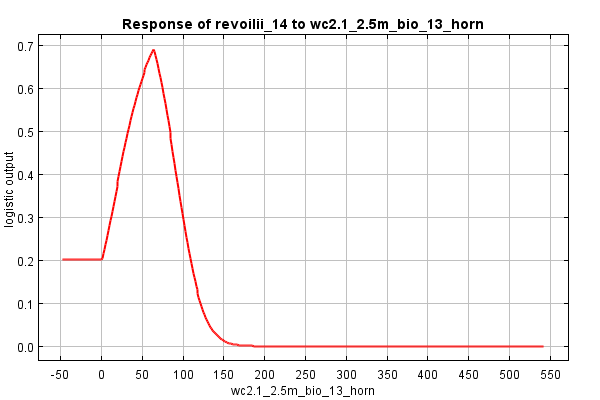

Supplement: Supplemental Information 6 [file peerj-08-9652-s006.zip › Data_S6_Ecological_Niche_Modeling/4_final_Maxent_analysis/output/plots/revoilii_14_wc2.1_2.5m_bio_13_horn_only.png]

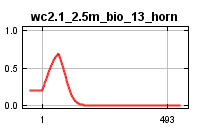

Supplement: Supplemental Information 6 [file peerj-08-9652-s006.zip › Data_S6_Ecological_Niche_Modeling/4_final_Maxent_analysis/output/plots/revoilii_14_wc2.1_2.5m_bio_13_horn_only_thumb.png]

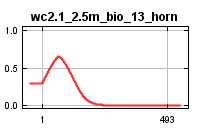

Supplement: Supplemental Information 6 [file peerj-08-9652-s006.zip › Data_S6_Ecological_Niche_Modeling/4_final_Maxent_analysis/output/plots/revoilii_14_wc2.1_2.5m_bio_13_horn_thumb.png]

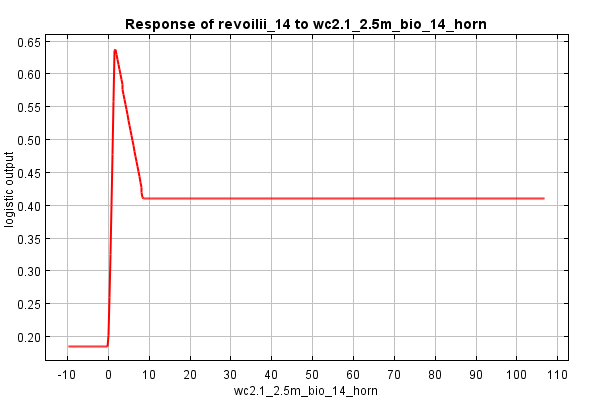

Supplement: Supplemental Information 6 [file peerj-08-9652-s006.zip › Data_S6_Ecological_Niche_Modeling/4_final_Maxent_analysis/output/plots/revoilii_14_wc2.1_2.5m_bio_14_horn.png]

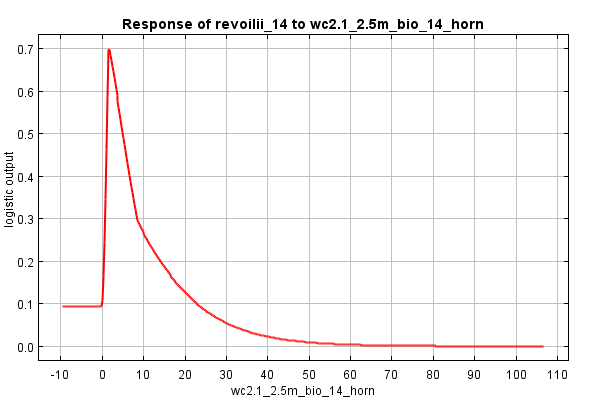

Supplement: Supplemental Information 6 [file peerj-08-9652-s006.zip › Data_S6_Ecological_Niche_Modeling/4_final_Maxent_analysis/output/plots/revoilii_14_wc2.1_2.5m_bio_14_horn_only.png]

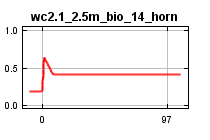

Supplement: Supplemental Information 6 [file peerj-08-9652-s006.zip › Data_S6_Ecological_Niche_Modeling/4_final_Maxent_analysis/output/plots/revoilii_14_wc2.1_2.5m_bio_14_horn_thumb.png]

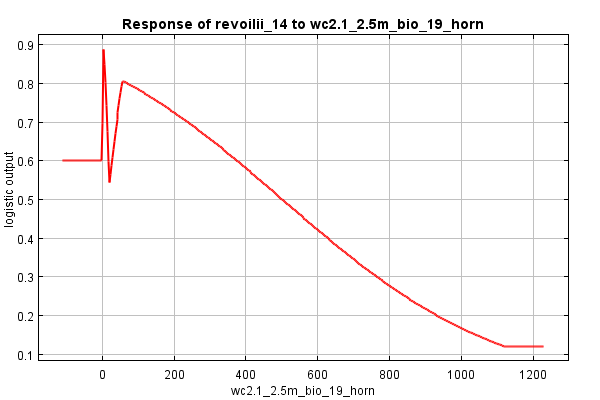

Supplement: Supplemental Information 6 [file peerj-08-9652-s006.zip › Data_S6_Ecological_Niche_Modeling/4_final_Maxent_analysis/output/plots/revoilii_14_wc2.1_2.5m_bio_19_horn.png]

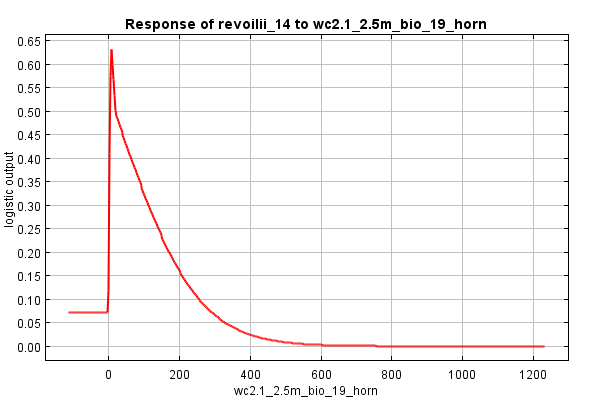

Supplement: Supplemental Information 6 [file peerj-08-9652-s006.zip › Data_S6_Ecological_Niche_Modeling/4_final_Maxent_analysis/output/plots/revoilii_14_wc2.1_2.5m_bio_19_horn_only.png]

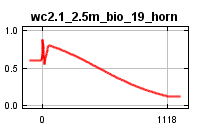

Supplement: Supplemental Information 6 [file peerj-08-9652-s006.zip › Data_S6_Ecological_Niche_Modeling/4_final_Maxent_analysis/output/plots/revoilii_14_wc2.1_2.5m_bio_19_horn_thumb.png]

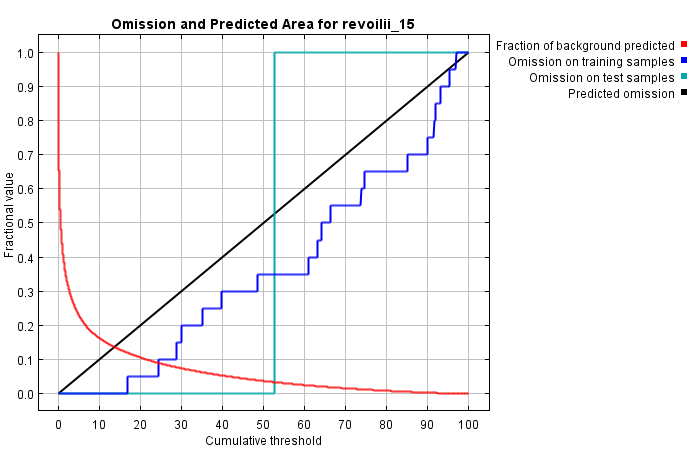

Supplement: Supplemental Information 6 [file peerj-08-9652-s006.zip › Data_S6_Ecological_Niche_Modeling/4_final_Maxent_analysis/output/plots/revoilii_15_omission.png]

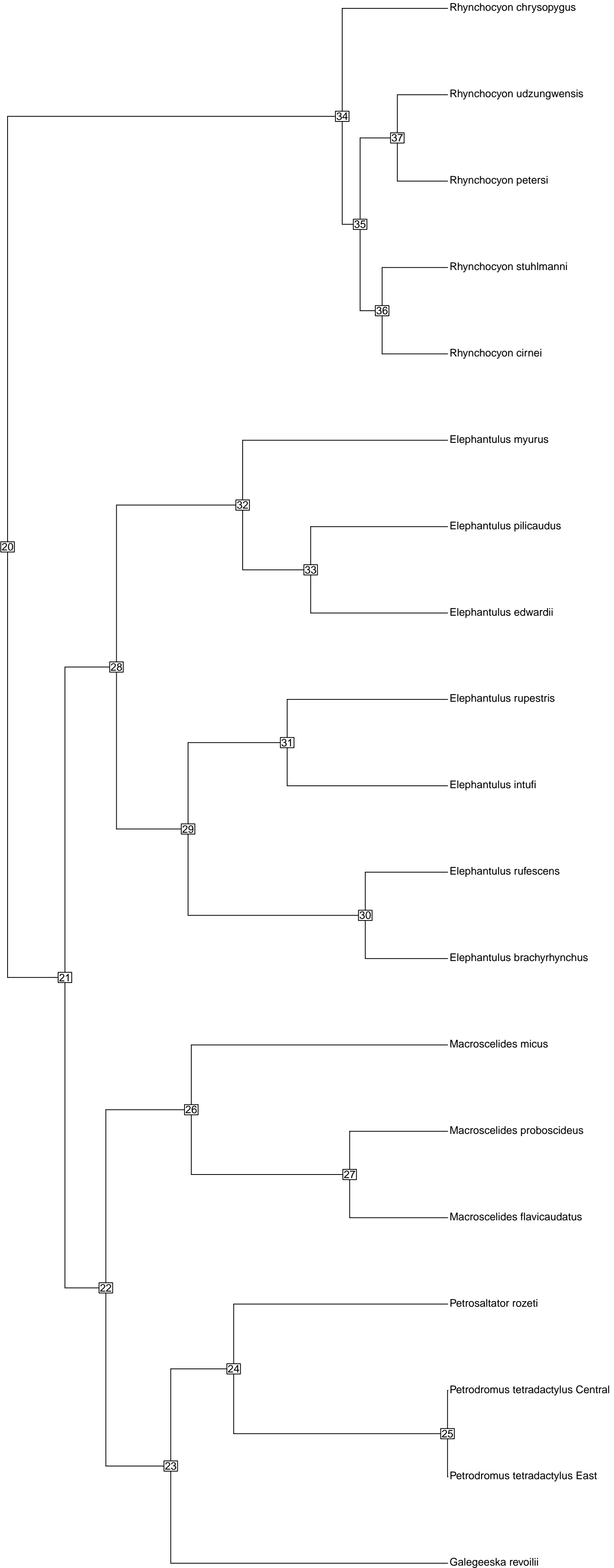

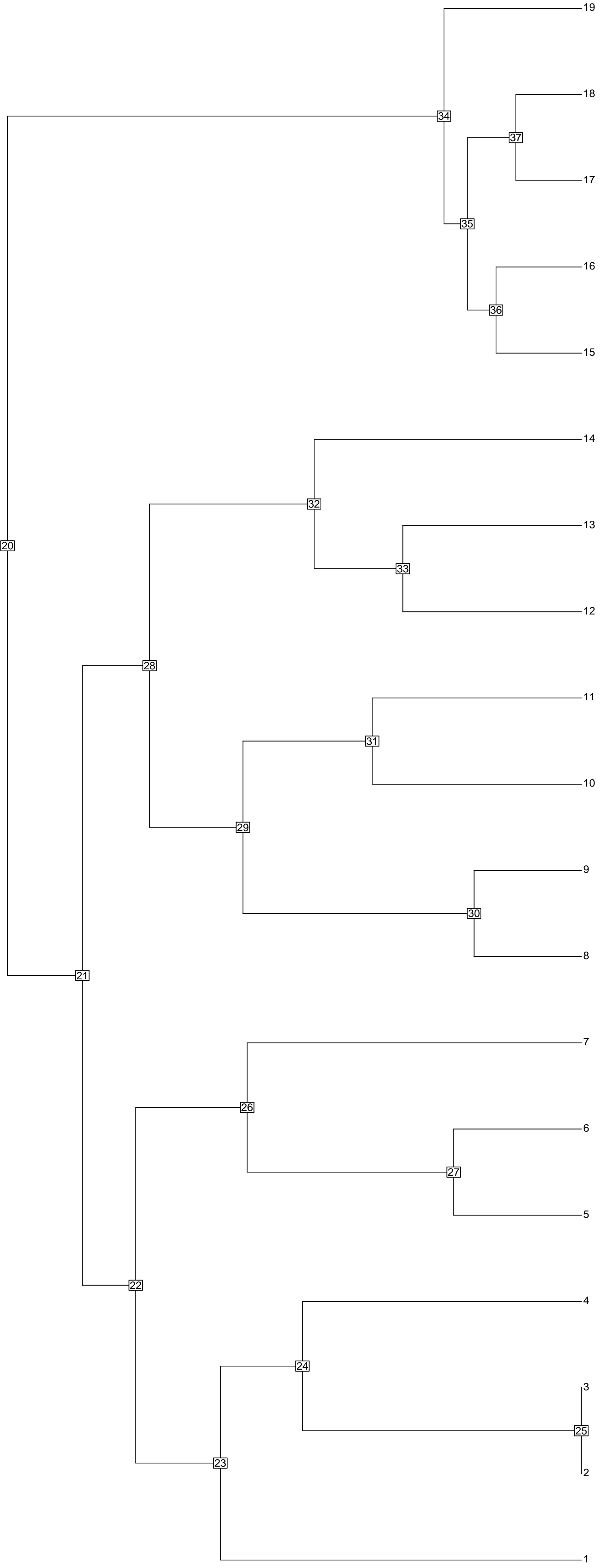

Supplement: Supplemental Information 7 [file peerj-08-9652-s007.zip › Data_S7_Biogeography_Analysis_Data_and_Settings/2_BayesTraits/BayesTraits_results/y_Tree_with_node_and_tip_numbers.pdf]

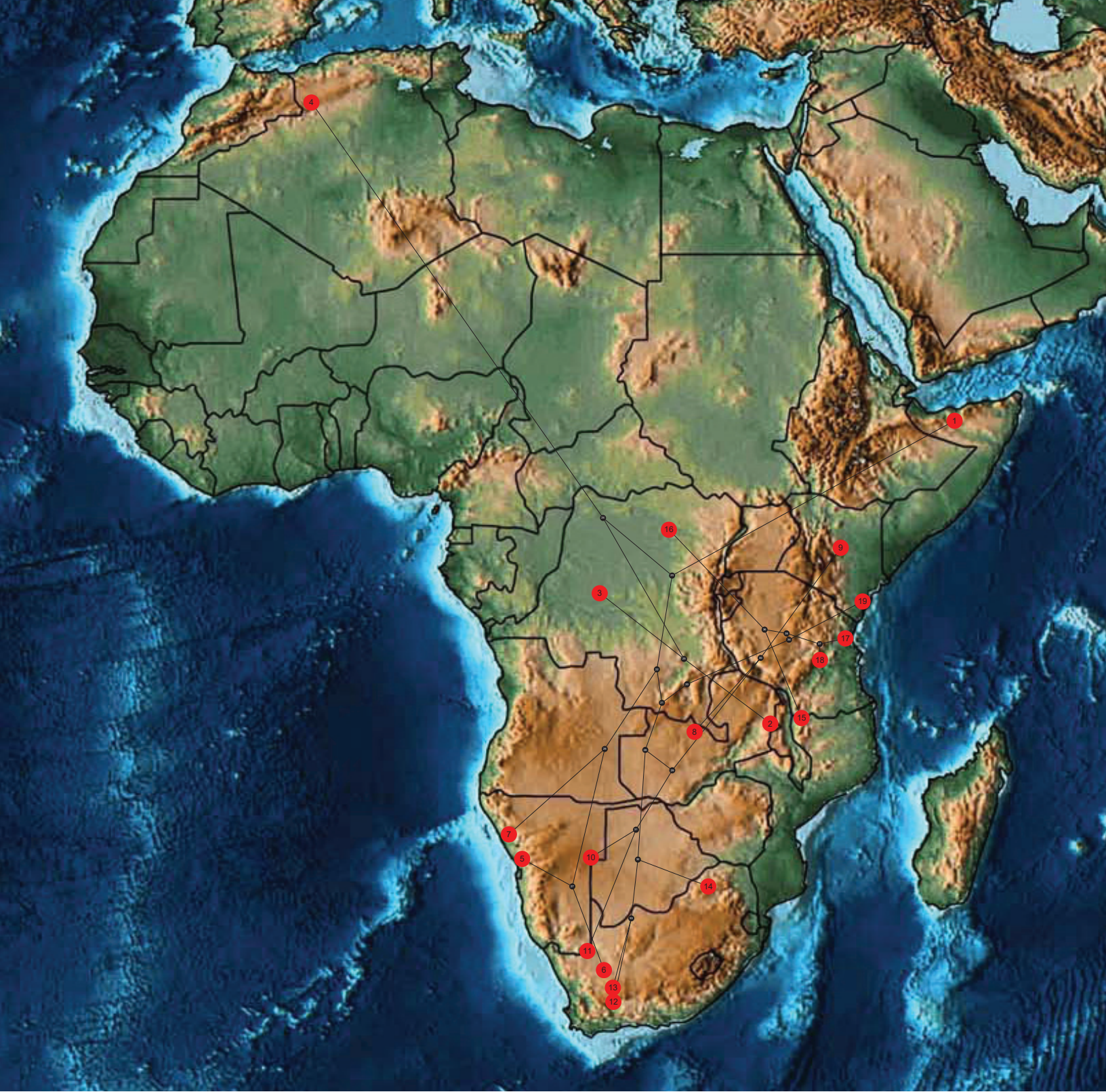

Supplement: Supplemental Information 7 [file peerj-08-9652-s007.zip › Data_S7_Biogeography_Analysis_Data_and_Settings/2_BayesTraits/BayesTraits_results/z_Results_plotted_to_map.pdf]
